# Supplementary material for: Health-related quality of life and estimation of the minimally important difference in the Functional Assessment of Cancer Therapy-Endocrine Symptom score in postmenopausal ER+/HER2- metastatic breast cancer with low sensitivity to endocrine therapy
Source: PLoS One. 2022 Nov 29;17(11):e0278344. doi: 10.1371/journal.pone.0278344 (PMC9707754; doi:10.1371/journal.pone.0278344)
Supplement: S1 File — (DOCX) [file pone.0278344.s002.docx]

All procedures were performed in accordance with the Helsinki declaration and the institutional review board at each study site approved the final protocol. Written informed consent was obtained from all study participants.

The names of the ethics committees/institutional review boards are listed here.

1. Ethics committee of Okayama university graduate school of medicine
2. Gunma cancer center review board
3. Kobe city medical center general hospital certified review board
4. Nagano city hospital review board
5. Naha-nishi clinic review board
6. National cancer center east ethics committee
7. Aomori-chuo prefectural hospital review board
8. Asahikawa koseiren asahikawa hospital review board
9. Ethics committee of Tokyo medical university
10. Ethics committee of Chiba university hospital
11. Osaka medical center review board
12. Osaka breast clinic review board
13. Hokkaido cancer center review board
14. Ethics committee of Tohoku university hospital
15. Ethics committee of the Cancer Institute Hospital of JFCR
16. Ethics committee of Hiroshima university hospital
17. Ehime-chuo prefectural hospital review board
18. Ethics committee of Nagoya city university hospital
19. Ethics committee of Asahi university hospital
20. Hyogo cancer center review board
21. Ethics committee of Kumamoto university hospital
22. Kumamoto Shinto General Hospital review board
23. Ota memorial hospital review board
24. Yao city hospital review board
25. Toranomon hospital review board
26. Kure-iryo center review board
27. Hiroshima city hospital review board
28. Kyushu cancer center review board
29. Yodogawa Christian Hospital review board
30. Ethics committee of Nagoya university hospital
