# Supplementary material for: Health-related quality of life and estimation of the minimally important difference in the Functional Assessment of Cancer Therapy-Endocrine Symptom score in postmenopausal ER+/HER2- metastatic breast cancer with low sensitivity to endocrine therapy
Source: PLoS One. 2022 Nov 29;17(11):e0278344. doi: 10.1371/journal.pone.0278344 (PMC9707754; doi:10.1371/journal.pone.0278344)
Supplement: S1 Protocol — (PDF) [file pone.0278344.s003.pdf]

HORSE-BC

HOrmonal therapy ReSistant Er positive
metastatic Breast Cancer cohort study

Clinical study support project of the General Incorporated Association of CSPOR-BC

PROTOCOL
Hormonal therapy resistant estrogen-receptor positive metastatic breast cancer cohort study

Research representatives responsible for the preparation of this protocol:

Naruto Taira — Department of Breast and Endocrine Surgery,
Okayama University Hospital
2-5-1 Shikatacho, Okayama Kita-ku, Okayama, 700-8558 Japan
PHONE: 086-235-7265
FAX: 086-235-7269
E-mail: ntaira@md.okayama-u.ac.jp

Tomomi Fujisawa — Department of Breast Oncology,
Gunma Prefectural Cancer Center
617-1 Takahayashinishicho, Ota, Gunma, 373-8550 Japan
PHONE: 0276-38-077
FAX: 0276-38-8386
E-mail: fujisawa@gunma-cc.jp

Research secretariat: General Incorporated Association of CSPOR-BC
Ochanomizu Kimura Building 4F, 2-19-3 Sotokanda,
Chiyoda-ku, Tokyo, 101-0021 Japan
PHONE: 03-5294-7288
FAX: 03-5294-7290
E-mail: trial-bc@cspor-bc.or.jp

Data Center (in the Japan Clinical Research Support Unit)

FAX: 03-5298-8536, PHONE: 03-3254-8029

Reception opening times: 10 am–5 pm, weekdays

(Closed on public holidays, Saturday, Sunday, and year-end and New Year holidays)

Preparation of original draft: March 25, 2015
Approval from CSPOR-BC Steering Committee: August 12, 2015
Preparation of Ver. 1.0: August 12, 2015

Contents

0 Outline 5
0.1 Study design 5
0.2 Objectives 5
0.3 Research hypothesis 6
0.4 Subjects 6
0.5 Methods 6
0.6 Outcome measures 6
0.7 Estimated enrollment and estimated study duration 6
1 Title of the study 7
2 Implementation structure of the study 8
2.1 Executive committee 8
2.2 Mission of the Executive Committee 8
2.3 General Incorporated Association of CSPOR-BC Steering Committee 8
2.4 Mission of the steering committee 9
2.5 CSPOR-BC Study Review Committee 9
2.6 Mission of the Study Review Committee 9
2.7 Independent Data Monitoring Committee of the General Incorporated Association of CSPOR-BC . 10
2.8 Data Management Committee of the General Incorporated Association of CSPOR-BC 10
2.9 CSPOR Data Center 10
2.10 General Incorporated Association of CSPOR-BC Secretariat 11
2.11 Collaborating research institutions 11
3 Definition of terms used in this study 12
3.1 Compliance with "Ethical Guidelines for Medical and Health Research Involving Human Subjects" 12
3.2 Breast cancer with low sensitivity to the primary endocrine therapy 12
3.3 Classification of clinical stages 13
3.4 Histological classification 15
3.5 Evaluation of the Performance Status (PS) 15
3.6 Determination of expression of hormone receptors 16
3.7 Determination of HER2 expression status 17
3.8 Response evaluation 18
3.9 Definition of outcome measures in this study 25
4 Objectives and significance of the study 28
4.1 Objectives of the study 28
4.2 Background inspiring this study 28
4.3 Study hypotheses 33
4.4 Significance of the study 33

5 Methods and duration of the study ... 34
5.1 Study design ... 34
5.2 Case registration ... 35
5.3 Treatment ... 36
5.4 Survey ... 40
5.5 Survey of health-related quality of life ... 48
5.6 Submission of data ... 50
5.7 Items described in reports ... 52
5.8 Handling of data ... 53
5.9 Estimated enrollment and estimated study duration ... 53
5.10 Method of analysis and assay ... 53
6 Subject selection policy ... 56
6.1 Definition of the subject of this study ... 56
6.2 Inclusion criteria ... 56
6.3 Exclusion criteria ... 58
7 Scientific rationale of the study ... 59
7.1 Choice of outcome measures ... 59
7.2 Scientific rationale and grounds for research hypothesis ... 59
7.3 Sample size determination ... 61
8 Protection of Patients ... 62
9 Procedures to obtain informed consent etc. ... 62
10 Handling of personal information ... 63
11 Burden and expected risks and benefits for study subjects ... 64
12 Methods of storage and disposal of specimens and information ... 64
12.1 Storage ... 64
12.2 Disposal ... 64
13 Contents and methods of report to Chief Executive of research implementing entity ... 64
13.1 Contents of report to Chief Executive of research implementing entity ... 64
13.2 Timing and methods of reporting ... 65
14 Research funding and conflicts of interest ... 65
14.1 Research funding ... 65
14.2 Disclosure of conflict of interest ... 65
15 Method of disclosure for information about the study ... 67
15.1 Registration of the outline and results of the study ... 67
15.2 Publication of study results ... 67
16 Response to consultation etc. from study subjects and involved persons ... 67
17 Informed consent by legally acceptable representative ... 67
18 Economic burden and rewards for study subjects ... 67

19 Evaluation and reporting of adverse events ... 68
19.1 Evaluation of adverse events ... 68
19.2 Reporting of adverse events ... 68
19.3 Responsibility of the secretariat ... 70
19.4 Examination by the independent data monitoring committee ... 70
20 Compensation for health hazards ... 71
21 Response to queries regarding the delivery of medical care after the completion of the study ... 71
22 Handling of information related to health conditions of study subjects and study results ... 71
23 Outsourcing of research works, the contents of outsourced works, and method of supervision of outsourcing contractors ... 73
23.1 Outsourcing of data management ... 73
23.2 Contents of works ... 73
23.3 Method of supervision ... 73
24 Future utilization of samples and information ... 73
25 Monitoring and audit ... 73
25.1 Monitoring ... 73
25.2 Audit ... 74
26 Approval by Ethical Review Boards ... 74
26.1 Approval at the start of participation in the study ... 74
26.2 Annual renewal of the approval by the ethical review board ... 74
27 Compliance and changes to the protocol ... 74
27.1 Completion, withdrawal, and interruption of the study ... 74
27.2 Compliance with the protocol ... 75
27.3 Deviation from the protocol ... 76
27.4 Changes to the protocol ... 76
28 Attribution of intellectual property rights ... 77
29 References ... 78
30 Appendices ... 81

0 Outline

0.1 Study design

Multicenter prospective observational study

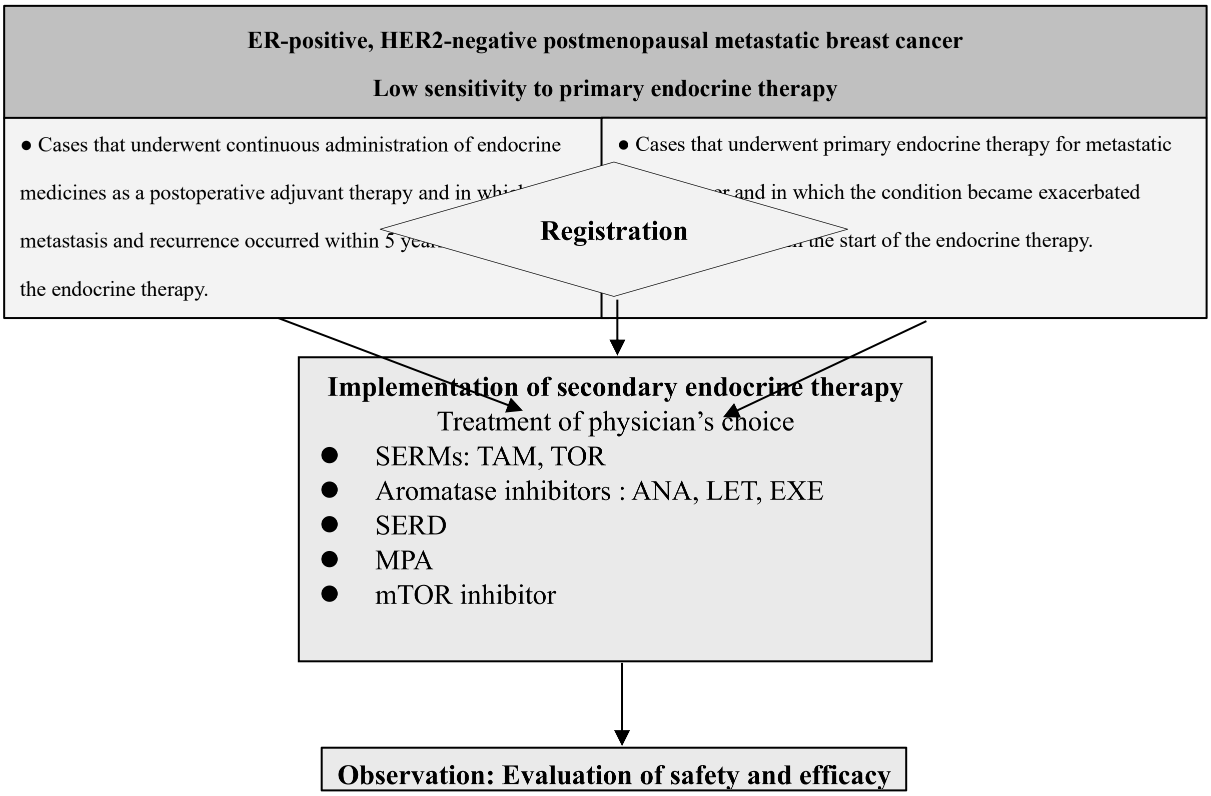

0.2 Objectives

(1) To demonstrate the efficacy and safety of secondary endocrine therapy in general as well as that of different drugs in estrogen receptor-positive, HER2-negative postmenopausal metastatic breast cancer in which the primary endocrine therapy did not have a favorable clinical effect (i.e., low sensitivity to primary endocrine therapy).

(2) To clarify the effects of reactivity to the previous endocrine therapy (the period from the start of postoperative endocrine therapy to recurrence) and the biological characteristics of each tumor (expression intensity of ER and presence or absence of PgR expression) on the results of the secondary endocrine therapy, and to obtain information to complement Hortobagyi's therapeutic algorithm.

0.3 Research hypothesis

(1) A clinical benefit rate of at least 30% can be expected for the latest endocrine therapeutic medicines for breast cancer with low sensitivity to the primary endocrine therapy.

(2) The effect of the secondary endocrine therapy can be predicted, because responsiveness to previous treatment and the biological characteristics of a given tumor influence the therapeutic effect of the secondary endocrine therapy on breast cancer with low sensitivity to the primary endocrine therapy.

0.4 Subjects

The subjects are estrogen receptor-positive, HER2-negative postmenopausal women with metastatic breast cancer with low sensitivity to the primary endocrine therapy. (See "6. Selection policy for subjects" for details.)

0.5 Methods

Treatment with current endocrine therapeutic medicines selected according to the preferences of medical professionals and the patients will be implemented to evaluate the efficacy and safety of the treatment through prospective observation.

0.6 Outcome measures

Primary outcome measures: Clinical benefit rate.

Secondary outcome measures: Progression free survival, overall survival, time to treatment failure, time to chemotherapy, response rate, health-related quality of life, adverse events.

0.7 Estimated enrollment and estimated study duration

Estimated enrollment: 200 or more.

Estimated study duration: Four years from November 2015 to October 2019

Of this period, the registration period will span 1 year from November 2015 to October 2016 and the observation period will take place from November 2016 to 3 years after registration of the last case.

1 Title of the study

(1) Japanese title of the study

Cohort study of secondary endocrine therapy for hormonal therapy resistant estrogen receptor positive metastatic breast cancer

(内分泌療法耐性エストロゲン受容体陽性転移乳がんに対する二次内分泌療法のコホート研究)

(2) Japanese brief title

Hormonal therapy resistant metastatic breast cancer cohort study

(内分泌療法耐性転移乳がんのコホート研究)

(3) English title of the study

Hormonal therapy resistant estrogen receptor positive metastatic breast cancer cohort study

(4) English brief title

HORSE-BC

2 Implementation structure of the study

This study will be conducted as a research support project of the General Incorporated Association of Comprehensive Support Project for Oncological Research of Breast Cancer (hereinafter referred to as "CSPOR-BC").

An executive committee has been established to implement this study. In addition, a steering committee, study review committee, independent data monitoring committee, and data management committee have been organized by the General Incorporated Association of CSPOR-BC to conduct review of the study protocol, examination and decision of study policies, and management and supervision of the study operation of this research organization.

2.1 Executive committee

Committee Chairperson (representative for the observational study)

Naruto Taira (Department of Breast and Endocrine Surgery, Okayama University Hospital), Taking charge of the Health Outcome Research (HOR)

Tomomi Fujisawa (Department of Breast Oncology, Gunma Prefectural Cancer)

Committee members (in order of the Japanese syllabary)

Kazuhiro Araki (Breast Medical Oncology Department, Breast Oncology Center, The Cancer Institute Hospital of JFCR)

Takayuki Iwamoto (Department of Breast and Endocrine Surgery, Okayama University Hospital)

Biostatistician (person in charge of statistical analyses)

Kentaro Sakamaki (Department of Biostatistics and Epidemiology, Yokohama City University Medical Center)

2.2 Mission of the Executive Committee

- Coordination of the implementation of the study conducted in collaboration with the Data Center and Secretariat.
- Reporting of the implementation status of the study to the Steering Committee.
- Matters required for the management and assurance of the quality of the study.
- Evaluation of the quality of facilities participating in the study.
- Support of the Data Center for data management.
- Support of the statisticians for statistical analyses.
- Preparation of reports.
- Other matters important for the smooth and effective implementation of the study.

2.3 General Incorporated Association of CSPOR-BC Steering Committee

Committee Chairperson

Hirofumi Mukai (Division of Breast and Medical Oncology, National Cancer Center Hospital East)

Committee members (in order of the Japanese syllabary)

Tomohiko Aihara	(Chief Director of Keimeikai Aihara Hospital)
Hiroji Iwata	(Department of Breast Oncology, Aichi Cancer Center Hospital)
Shozo Ohsumi	(Department of Breast Oncology, National Hospital Organization, Shikoku Cancer Center)
Masato Takahashi	(Department of Breast Surgery, National Hospital Organization, Hokkaido Cancer Center)
Yasuo Hozumi	(Department of Breast Oncology, Jichi Medical University Hospital)
Seiichiro Yamamoto	(Center for Cancer Control and Information Services, National Cancer Center)

2.4 Mission of the steering committee

- Development of the long-term plan and annual plans for the program, and reporting of study activities to the Foundation.
- Development of the budget to implement the program and report of the account settlement to the Foundation.
- Establishment, improvement, and elimination of subcommittees to implement the program, and appointment of subcommittee members.
- Supervision of the activities of the subcommittees.
- Other matters required to implement the objectives of this program.

2.5 CSPOR-BC Study Review Committee

Committee Chairperson

Tatsuya Toyama (Department of Breast and Endocrine Surgery, Nagoya City University Hospital)

Committee Vice-chairperson

Youngjin Park (Department of Breast Surgery, Tohoku Pharmaceutical University Hospital)

Committee members (statistician and others, in order of the Japanese syllabary)

Satoshi Teramukai	(Statistician; Department of Biostatistics, Kyoto Prefectural University of Medicine)
Toshimi Takano	(Department of Medical Oncology, Toranomon Hospital)
Kenji Tamura	(Division of Breast and Medical Oncology, National Cancer Center Hospital)
Nobuaki Matsubara	(Division of Breast and Medical Oncology, National Cancer Center Hospital East)

2.6 Mission of the Study Review Committee

- Decisions on the new commencement of clinical trials and accompanying studies, and decisions

on the application outline and adoption of studies at open recruitment of studies.

- Appointment of executive committee members of each clinical trial and accompanying study.
- Supervision of the implementation of clinical trials and accompanying studies.
- Approval of oral presentations and publications related to clinical trials and accompanying studies.
- Matters required for the smooth implementation of clinical trials and accompanying studies of other programs, and coordination among studies.

2.7 Independent Data Monitoring Committee of the General Incorporated Association of CSPOR-BC

Committee Chairperson

Seigo Nakamura (Department of Breast Surgical Oncology, Showa University Hospital)

Committee Vice-chairperson

Noriyuki Katsumata (Department of Oncology,
Nippon Medical School, Musashi Kosugi Hospital)

Committee members (in order of the Japanese syllabary)

Taro Shibata (Multi-institutional Clinical Trial Support Center,
National Cancer Center)

Akihiro Yanagisawa (Cancer Net Japan, NPO)

2.8 Data Management Committee of the General Incorporated Association of CSPOR-BC

Committee Chairperson

Shozo Ohsumi (Department of Breast Oncology,
National Hospital Organization, Shikoku Cancer Center)

Committee members (in order of the Japanese syllabary)

Masataka Sawaki (Department of Breast Oncology, Aichi Cancer Center Hospital)

Naruto Taira (Department of Breast and Endocrine Surgery, Okayama University Hospital)

2.9 CSPOR Data Center

Provides services including case registration, progress management, and data management.

Located in the Japan Clinical Research Support Unit (J-CRSU), NPO

J-CRSU Data Center

Representative (Director of the Data Center)

Yasuo Ohashi (Department of Integrated Science and Engineering for Sustainable Society,
Faculty of Science and Engineering, Chuo University)

Yushima D&A building 1F, 1-10-5 Yushima, Bunkyo-ku, Tokyo, 113-0034 Japan

TEL: 03-3254-8029

FAX: 03-5298-8536

E-mail: trial-bc@cspor-bc.or.jp

2.10 General Incorporated Association of CSPOR-BC Secretariat

Director-general of the Secretariat

Tomohiko Aihara (Chief Director of Keimeikai Aihara Hospital)

Division Manager of the Secretariat

Yoshiteru Ishikawa

Ochanomizu Kimura Building 4F, 2-19-3 Sotokanda, Chiyoda-ku, Tokyo, 101-0021 Japan

General Incorporated Association of CSPOR-BC

TEL: 03-5294-7288

FAX: 03-5294-7290

E-mail: trial-bc@cspor-bc.or.jp

2.11 Collaborating research institutions

See http://cspor-bc.or.jp/index.html for the latest list of collaborating research institutions and principal investigators in the institutions.

3 Definition of terms used in this study

3.1 Compliance with "Ethical Guidelines for Medical and Health Research Involving Human Subjects"

Terms used in this study comply with the "Glossary" described in Chapter 1, Part 2 of the "Ethical Guidelines for Medical and Health Research Involving Human Subjects" (Appendix A), which was issued by the Ministry of Education, Culture, Sports, Science and Technology and the Ministry of Health, Labour and Welfare of Japan on December 22, 2014.

Invasiveness; intervention; information utilized in research; specimen and/or information; existing specimen and/or information; research subject; research implementing entity; collaborative research implementing entity; organization collecting and providing specimens or information; investigator, etc.; principal investigator; chief executive of research implementing entity; ethical review committee; informed consent; legally acceptable representative; legally acceptable representative, etc.; personal information; personal information, etc.; anonymization; linkable anonymization; unlinkable anonymization; adverse event; serious adverse event; unexpected serious adverse event; monitoring; audit

3.2 Breast cancer with low sensitivity to the primary endocrine therapy

Cases of patients who continue post-operative adjuvant endocrine therapy and experience recurrence within 5 years from the initiation of the endocrine therapy or cases of metastatic breast cancer receiving primary endocrine therapy who showed progression of the disease within 9 months from the initiation of the endocrine therapy are called <u>breast cancer with low sensitivity to the primary endocrine therapy</u> in this study.

3.3 Classification of clinical stages

"General rules for clinical and pathological recording of breast cancer (17th ed., 2012)" is used [1). Compliant with the UICC-TNM classification (7th ed., 2009).

(1) T: Primary tumor [Note 1]

		Size (cm)	Chest wall involvement [Note 2]	Skin edema/ulceration, satellite skin nodules
TX		Tumor cannot be evaluated		
Tis		Invasive carcinoma or Paget's disease		
T0		No primary tumor is observed [Notes 3 and 4]		
T1 [Note 5]		≤ 2.0	-	-
T2		> 2.0<br>≤ 5.0	-	-
T3		> 5.0	-	-
T4	a	Regardless of the size	+	-
	b		-	+
	c		+	+
	d	Inflammatory breast cancer [Note 6]		

Note 1: The envisioned size of T is the maximum diameter of infiltration of the primary tumor and is holistically determined by visual inspection, palpation, and imaging. When the tumor contains high levels of intraductal components and there is a large difference between the diameter of infiltration obtained by palpation and that obtained by imaging, the result obtained by imaging will be prioritized. When many tumors are present in the mammary gland, the size will be evaluated using the largest T.

Note 2: The chest wall means the rib, intercostal muscle, and serratus anterior muscle, and does not include the pectoral muscle.

Note 3: The location of the primary tumor cannot be confirmed by visual inspection, palpation, and imaging.

Note 4: Cases with abnormal nipple discharge and cases with calcification observed by mammography are not classified as T0, and the determination of these cases will be suspended. These cases will be definitively classified into Tis, T1mic, or others by final pathological diagnosis.

Note 5: T1 cases are subclassified into a (≤ 0.5), b (> 0.5 and ≤ 1.0), and c (> 1.0 and ≤ 2.0). However, tumors with a histological diameter of infiltration of ≤ 0.1 cm will be described as T1mic.

Note 6: Usually the term inflammatory breast cancer is used to describe a condition that shows no mass but diffuse redness, edema, and induration of the skin. Local redness and edema occurring together with detectable growth(s) and progress of mass is not included in this category.

(2) N: Regional lymph node [Note 1]

		Ipsilateral axillary nodes, Level I, II: Movable axillary nodes	Ipsilateral axillary nodes, Level I, II: Fixation to surrounding tissue or adhesion of lymph nodes	Parasternal lymph nodes	Ipsilateral axillary nodes, Level III [Note 2]	Ipsilateral supraclavicular lymph nodes
NX		Lymph nodes cannot be evaluated				
N0		-	-	-	-	-
N1		+	-	-	-	-
N2	a	-	+	-	-	-
	b	-	-	+	-	-
N3	a	+/-	OR +/-	+/-	+	-
	b	+	OR +	+	-	-
	c	+/-	OR +/-	+/-	+/-	+

Note 1: Diagnosis of lymph node metastasis is done by palpation and imaging.

Note 2: This corresponds to the infraclavicular lymph nodes described in the UICC-TNM classification (7th ed.).

(3) M: Distant metastasis

M0: No distant metastasis

M1: Metastasis to distant organs

(4) TNM classification

		T0	T1	T2	T3	T4
M0	N0		I	IIA	IIB	IIIB
	N1	IIA	IIA	IIB	IIIA	IIIB
	N2	IIIA	IIIA	IIIA	IIIA	IIIB
	N3	IIIC	IIIC	IIIC	IIIC	IIIC
M1		IV	IV	IV	IV	IV

3.4 Histological classification

"General rules for clinical and pathological recording of breast cancer (17th ed., 2012)" will be used [1].

1 Non-invasive carcinoma

- 1a. Non-invasive ductal carcinoma
- 1b. Non-invasive lobular carcinoma

2 Invasive carcinoma

- 2a. Invasive ductal carcinoma
- 2a1. Papillotubular carcinoma
- 2a2. Solid-tubular carcinoma
- 2a3. Scirrhous carcinoma
- 2b. Special types
- 2b1. Mucinous carcinoma
- 2b2. Medullary carcinoma
- 2b3. Invasive lobular carcinoma
- 2b4. Adenoid cystic carcinoma
- 2b5. Squamous cell carcinoma
- 2b6. Spindle cell carcinoma
- 2b7. Apocrine carcinoma
- 2b8. Carcinoma with cartilaginous and/or metaplasia
- 2b9. Tubular carcinoma
- 2b10. Secretory carcinoma (juvenile carcinoma)
- 2b11. Invasive micropapillary carcinoma
- 2b12. Matrix producing carcinoma
- 2b13. Other

3 Paget's disease

3.5 Evaluation of the Performance Status (PS)

The following Japanese version of the ECOG scale is used.

Grade	Performance Status
0	Fully active, able to carry on all pre-disease performance without restriction.
1	Restricted in physically strenuous activity but ambulatory and able to carry out work of a light or sedentary nature, e.g., light house work, office work.
2	Ambulatory and capable of all self-care but unable to carry out any work activities. Mobile during more than 50% of waking hours.
3	Capable of only limited self-care, confined to bed or chair during more than 50% of waking hours.
4	Completely disabled. Cannot carry out any self-care. Totally confined to bed or chair.

3.6 Determination of expression of hormone receptors

(1) Expression of estrogen receptor (ER)

Expression of estrogen receptor α (ERα) is evaluated by an immunohistochemical (IHC) method. Evaluation will be conducted at each facility, and ER positivity will be determined based on the following criteria in this study.

J-score classification: A tissue area of ER-staining-positive cells of 1% or higher is determined as positive. The Allred score classification is calculated by conversion of the proportion score.

(2) Expression of progesterone receptor (PgR)

Expression of progesterone receptor (PgR) is evaluated by an IHC method. Evaluation will be conducted at each facility, and PgR positivity will be determined based on the following criteria in this study.

J-score classification: A tissue area of PgR-staining-positive cells of 1% or higher is determined as positive. The Allred score classification is calculated by conversion of the proportion score.

3.7 Determination of HER2 expression status

For HER2 expression status, "Guidelines for HER2 testing in breast cancer, 4th ed." developed by the Breast Cancer HER2 Testing and Pathology Section, [2)] will be applied to the determination criteria of IHC and *in situ* hybridization (ISH) methods.

(1) Determination criteria for IHC method

Based on the determination criteria below, scores of 0 and 1+ are defined as HER2 negative, and a score of ≥ 3 is defined as HER2 positive.

When a case is determined as equivocal (score = 2), a reflex test (using ISH method with the same specimen) or a new test (using IHC or ISH methods and with a new specimen if possible) must be conducted.

Determination	Score	Staining pattern
Positive	3+	The rate of cancer cells with intense and complete positive staining of the cell membrane is > 10%.
Equivocal	2+	① The rate of cancer cells with mild to moderate and complete positive staining of the cell membrane is > 10%.<br>② The rate of cancer cells with intense and complete positive staining of the cell membrane is ≤ 10%.
Negative	1+	The rate of cancer cells with almost indistinguishable faint staining of cell membrane is > 10%.
	0	No positive staining of cell membrane, or the rate of cancer cells with positive staining of cell membrane is ≤ 10%.

(2) Determination criteria for ISH method

HER2 positivity is defined according to the determination criteria below:

Determination	Criteria
Positive	Single probe:<br>Mean HER2 copy number ≥ 6.0<br>Dual probe:<br>HER2/CEP17 ratio ≥ 2.0; Mean HER2 copy number ≥ 4.0,<br>HER2/CEP17 ratio ≥ 2.0; Mean HER2 copy number < 4.0,<br>HER2/CEP17 ratio < 2.0; Mean HER2 copy number ≥ 6.0
Equivocal	Single probe:<br>Mean HER2 copy number ≥ 4.0, < 6.0<br>Dual probe:<br>ER2/CEP17 ratio < 2.0; Mean HER2 copy number ≥ 4.0, < 6.0
Negative	Single probe:<br>Mean HER2 copy number < 4.0<br>Dual probe:<br>ER2/CEP17 ratio < 2.0; Mean HER2 copy number < 4.0

3.8 Response evaluation

The tumor reduction effect will be evaluated in cases that have lesions measurable by RECIST guidelines (version 1.1) to calculate the response rate. The rate of subjects for whom the best overall response is complete response (CR) or partial response (PR), is defined as the response rate [3)].

(1) Definition of measurable lesion

Lesions that correspond to at least 1 of the following terms are referred to as "measurable lesions".

1） Lesions other than lymph node lesion (non lymph node lesions) that satisfy at least one of the following criteria:

① The maximum diameter observed by CT using a slice thickness of ≤ 5 mm is ≥ 10 mm.

② The maximum diameter observed by CT using a slice thickness of > 5 mm is at least 2 times thicker than the slice thickness.

③ Osteolytic bone metastasis lesions with soft tissue lesion(s) satisfying ① or ②.

④ Cystic metastasis lesions satisfying ① or ② when there are no other measurable non-cystic lesions.

2） Lymph node lesions with a short diameter of ≥ 15 mm observed by CT using a slice thickness of ≤ 5 mm. Lymph node lesions with a short diameter of ≥ 10 mm and < 15 mm are non-measurable lesions, and lymph node lesions with a short diameter of < 10 mm are not lesions.

3） The maximum diameter observed by chest simple radiographic imaging is ≥ 20 mm, and the lesion is surrounded by lung tissue (i.e., the lesion has no contact with the mediastinum or chest wall).

4） Superficial lesions with a maximum diameter of ≥ 10 mm or more that can be photographed in color with a measuring ruler (cutaneous metastasis, etc.).

All lesions that do not satisfy the above terms will be referred to as "non-measurable lesions". The following lesions, etc. are referred to "non-measurable lesions" regardless of the test methods used and the size of the lesions:

- Bone lesions (except for osteolytic lesions with a measurable soft tissue component).
- Cystic lesions (except for those meeting the above criterion given in {1)-④}).
- Lesions with a history of local treatment such as radiotherapy.
- Meningeal lesions.
- Ascites, pleural effusion, and cardiac effusion.
- Inflammatory breast cancer.

- Skin/lung lymphangitis.
- Palpable abdominal tumors and abdominal organ enlargement that cannot be confirmed by imaging.

(2) Selection of target lesion and baseline evaluation

Of the measurable lesions observed at the registration of this study (baseline), the top 5 largest lesions in diameter (the long diameter for non-lymph node lesions and the short diameter for lymph node lesions) will be selected as target lesions, with at most 2 lesions selected from each organ. The lesions will be selected with the consideration that the selected lesions should be present in organs with measurable lesions spread evenly as much as possible and should show good reproducibility of measurement on repeated examination (i.e., ease of measurement). The site, testing method, date of test, long diameter of non-lymph node lesions, short diameter of lymph node lesions, and sum of the long and short diameters of the selected target lesions will be recorded in the order of cranial to caudal localization.

(3) Selection of non-target lesions and baseline evaluation

For all lesions that are not selected as target lesions, the site, testing method, and date of test will be recorded as "non-target lesions" regardless of whether the lesions are measurable or not. When a number of similar lesions are observed in the same region of the same organ, such lesions may be recorded as a single non-target lesion.
(Examples: multiple pelvic lymphadenopathy and multiple hepatic metastasis.)

(4) Determination of tumor reduction effect

Therapeutic response evaluation will be conducted at the discretions of primary physicians because this study is observational in nature. However, exacerbation of the condition may be observed in the early period after the initiation of the treatment because the study is on breast cancer with low sensitivity to endocrine therapy. Response evaluations will be conducted at about 3 and 6 months to ensure the safety of research subjects and avoid missing the optimum timing for determining treatment policy.

Evaluation of target and non-target lesions will be conducted using the same test methods as those used before registration, including the same photographing conditions such as contrast agent and slice thickness to measure the major axis of the target lesion (the short diameter for lymph node) and to record the disappearance of non-target lesions, the presence or absence of progression, and the presence or absence of new lesions.

Response evaluation for target lesions

CR (Complete response)	When all non-lymph node lesions disappear and the short diameters of all lymph node target lesions become < 10 mm.
PR (Partial response)	When the sum of diameters of the target lesions becomes at least 30% smaller than their corresponding sum of diameters at baseline.
SD (Stable disease)	When no tumor regression corresponding to PR or tumor enlargement corresponding to PD is observed.
PD (Progressive disease)	When a sum of diameters of a target lesion becomes at least 20% larger than the smallest sum of diameters measured previously and the absolute value of the sum of diameters increases by at least 5 mm (including the incidence of new lesions).
NE (Not evaluable)	When tests cannot be done for any reason or when a lesion cannot be determined to be any of CR, PR, PD, or SD.

Reduction rate of the longest sum of diameters = (sum of diameters before treatment - sum of diameters at evaluation) / (sum of diameters before treatment) × 100%

Increase rate of the longest sum of diameters = (sum of diameters at evaluation - the smallest sum of diameters) / (the smallest sum of diameters) × 100%

- Actual measurement values of the diameters of target lesions will be recorded as long as they are measurable (for example, even < 5 mm). However, when the diameter of a target lesion is determined as "non-measurable because it is too small", the diameter of the lesion will be recorded as 0 mm if it is determined that none of the tumor lesion remains, or as 5 mm if it is determined that some tumor lesion remains regardless of the slice thickness used for CT.
- When the reduction rate satisfies the condition for PR and the increase rate satisfies the condition for PD at the same time, the lesion will be considered to be PD.
- When lesions separate from a single lesion during treatment, these lesions will be added to each sum of diameters.
- When > 1 lesion fuses together and their boundaries become identifiable during treatment, the diameter of the fused lesion will be added to the sum of diameters. If the boundaries of the lesions are identifiable, the diameter of each lesion will be added to the sum of diameters even if the lesions share a margin.

Response evaluation for non-target lesions

CR<br>(Complete response)	When all non-lymph node lesions disappear, all tumor markers fall within the upper limit of the facility's normal reference values, and the short diameters of all lymph node target lesions become < 10 mm.
Non-CR/non-PD	When ≥ 1 non-lymph node lesions do not disappear, or the short diameters of ≥ 1 lymph node non-target lesions are ≥ 10 mm, or ≥ 1 tumor marker exceeds the upper limit of the facility's normal reference values.
PD<br>(Progressive disease)	"Obvious progression" of non-target lesions (including incidence of new lesions) (see the comments below the table for the definition of obvious progression).
NE<br>(Not evaluable)	When tests cannot be done for any reason, or when a lesion cannot be determined to be any of CR, non-CR/non-PD, or PD.

Obvious progression

- Cases with non-measurable lesions will be considered to show "obvious progression" when it is determined that the benefits of discontinuing the treatment are greater than those of continuing the treatment because of a notable exacerbation of non-target lesions, even if the target lesions are PR or SD.
- In cases with only non-measurable lesions, for example, when lesion volume is used as a guide, an increase in the volume of non-measurable lesions of 73% will be considered to be "obvious progression".

Incidence of new lesion(s)

- When a lesion that did not exist at baseline is observed after the initiation of treatment, this will be considered a "new lesion". However, it is also necessary to confirm that the “new lesion” is not observed due to a difference in the scan method or imaging modality compared with that used for baseline evaluation or a clinical condition other than a tumor. For example, a cystic lesion occurring in a focus caused by necrosis of liver metastasis will not be considered to be a "new lesion". Lesions that are newly found on examination of regions that were not regarded to be essential for examination at baseline will be considered to be new lesions.When a lesion that existed at baseline disappears and then appears again, such a lesion will be considered to be "PD" if this reappearance occurred after the overall response became CR; however, if other lesions are remaining, the lesion will not be considered to be a "new lesion" or "PD" because of the reappearance alone, but its tumor diameter will be added to the sum of diameters if the lesion is a target lesion. If the lesion is a non-target lesion, it will be considered to be "non-CR/non-PD" unless the lesion corresponds to "obvious progression".
- When a lesion may a new lesion but this cannot be confirmed, the lesion will not immediately be recorded as a new lesion, but imaging examination will be conducted after a clinically appropriate

interval. If the lesion is determined to be a new lesion by the imaging re-examination, the date of imaging examination when a new lesion was suspected shall be used as the date of incidence for the new lesion.

(5) Time point response

The time point response will be determined at each image evaluation time point in accordance with the criteria specified in the table below based on a combination of the response of target lesions, response of non-target lesion, and incidence of new lesions.

When there are no non-target lesions at baseline, the overall response will be determined based on the response of the target lesion and incidence of new lesions, and when there are no target lesions at baseline, the overall response will be determined based on the response of non-target lesions and incidence of new lesions in accordance with the criteria specified in the table below.

Determination criteria for time point response (when target lesions are present).

Response of target lesion	Response of non-target lesion	Incidence of new lesion	Overall response
CR	CR	No	CR
CR	Non-CR/Non-PD	No	PR
CR	Not evaluated	No	PR
PR	Non-PD or not fully evaluated	No	PR
SD	Non-PD or not fully evaluated	No	SD
Not fully evaluated	Non-PD	No	NE
PD	Any	Yes or No	PD
Any	PD	Yes or No	PD
Any	Any	Yes	PD

Determination criteria of time point response (when target lesions are absent).

Response of non-target lesions	Incidence of new lesions	Overall response
CR	No	CR
Non-CR/Non-PD	No	Non-CR/non-PD
Not fully evaluated	No	NE
Unequivocal PD	Yes or No	PD
Any	Yes	PD

(6) Best overall response

The best overall response will be considered in the order of CR > PR > SD > PD > NE. The overall evaluation at the best time point from the initiation of treatment through to the initiation of post-treatment will be considered to be the best overall response.

Meanwhile, determination of CR or PR in 4-week periods is not necessary in this study. When an overall response corresponds to the definition given for multiple categories, it will be categorized into the best-fitted category in the order of CR > PR > SD > PD > NE.

SD determination shall satisfy the SD standards after 6 weeks from the start of treatment. When an obvious deleterious change (exacerbation) of the disease is observed before the first effect determination, it will be considered to be PD when no imaging determination is implemented and NE when the determination was not conducted because of discontinuation of treatment due to adverse events or rejection by a subject.

(7) Clinical progression

Progression will be determined in accordance with the following criteria when using a method other than those defined in {3.8 (4) Determination of tumor reduction effect} or when progression in unmeasurable lesions is determined. However, progression will not immediately be determined even when a case corresponds to the following criteria but the judgment of a clinical investigator is prioritized. When image assessment is available, the image assessment will be prioritized.

① Confirmation of progression by ultrasound: When obvious progression such as emergence of new lesions, increased pleural effusion, etc. is observed by ultrasound.

② Confirmation of progression in bone lesion: When increased accumulation of bone is observed by scintigram or PET, or emergence of new lesions is observed in a bone lesion.

③ Exacerbation of subjective symptoms: When a clinical investigator determines there to have been progression, such as obvious exacerbation of bone pain in bone metastasis, obvious exacerbation of a sensation of dyspnea in lung metastasis, etc.

3.9 Definition of outcome measures in this study

(1) Clinical benefit rate (CBR)

The clinical benefit rate is defined as the rate of patients who are not determined to have experienced disease "progression" for 6 months from the initiation of treatment.

"Patients who are not determined as 'progression' for 6 months" means patients whose time point response {3.8 (5) Determination criteria of time point response} based on diagnostic imaging implemented at 3 and 6 months from the initiation of treatment is CR, PR, or SD at any time point, and for whom no clinical progression was observed for 6 months from the initiation of treatment {3.8 (7) Clinical progression}.

(2) Progression-free survival (PFS)

A period of time from the registration day to either of the day when progress was determined or the day of death from all causes (whichever is earlier). The following criteria are specified for the determination of PFS.

① "Progression" includes both progression in the determination of the total effect {3.8 (5) Determination criteria of time point response} and progression of the original disease based on a method other than diagnostic imaging {3.8 (7) Clinical progression}.
When the progression is determined based on diagnostic imaging, the date for which progression is recorded will be the day of the examination. In the case of clinical progression, the date of clinical determination will be recorded as the date of progression.

② In survival cases that are not determined to have experienced progression, the most recent day on which no progression is confirmed will be the recorded date for PFS.

③ When the diagnosis of progression is based on diagnostic imaging, the date recorded will be the "examination day" of the diagnostic imaging that provided "certain diagnosis" but not the day of examination where the progression was suspected as "suspicious on image". When the progression is determined clinically but is not based on diagnostic imaging, the date recorded for progression will be the day when the progression was determined.

④ The development of secondary cancer(s), e.g. heterochronic double cancer or heterochronic multiple cancer, will not be considered an event and will not be recorded as such, but will be considered to be PFS until another event is observed.

⑤ When treatment is discontinued due to the characteristics of or rejection by the patient and other treatment is implemented as an after-treatment, such cases will be handled in the same manner as the above for recording events and the endpoint, and will not be recorded at the time of discontinuation of treatment or the date of the initiation of after-treatment.

(3) Overall survival (OS)

The period of time from the registration day to the day of death from all causes. The final confirmation day of survival in survival cases will be the recorded date.

For cases that are lost to follow-up, the last date when survival of the patient was confirmed before becoming lost to follow-up will be the recorded date.

(4) Time to treatment failure (TTF)

A period of time from the registration day to either of the day when progress was determined, the day of death from all causes, or the day of discontinuation of the protocol treatment (whichever is earlier).

① The day of discontinuation of the protocol treatment is the day when the discontinuation will be determined.

② "Progression" includes both progression in determination of the total effect {3.8 (5) Determination criteria of time point response} and progression of the original disease based on methods other than diagnostic imaging {3.8 (7) Clinical progression}.
When the progression is determined based on diagnostic imaging, the date recorded for progression will be the day of the examination. In the case of clinical progression, the date recorded for progression will be the day of clinical determination.

③ When the diagnosis of progression is based on diagnostic imaging, the date recorded for the event will be the examination day when the diagnostic imaging was performed that provided certain diagnosis, but not the day of examination where progression was suspected as "suspicious on image". When the progression is determined clinically but not based on diagnostic imaging, the date recorded for progression will be the day when the progression was determined.

④ When the protocol treatment is continuously implemented and there is no progression, the treatment will be discontinued on the final confirmed day of survival (the final confirmed day of PFS).

(5) Time to chemotherapy (TTC)

The period of time from the registration day to the day of first administration of chemotherapeutic drug

① For cases where mortality occurred before the initiation of chemotherapy, the date of death will be the recorded date.

② For cases that are lost to follow-up and in which implementation of chemotherapy is not confirmable, the last date when survival of the patient was confirmed before becoming lost to follow-up will be the recorded date.

(6) Response rate (RR)

The response rate is the rate of patients whose best overall response {3.8(6) Best overall response} in a target population with measurable lesions is CR or PR as a proportion of all the cases that underwent treatment.

(7) Health-related quality of life (HRQoL)

See "5.5 Survey of health-related quality of life".

(8) Adverse events (toxicity)

Adverse events that are observed from registration to the discontinuation of the protocol treatment will be evaluated in all treated cases (see "19 Evaluation and reporting of adverse events").

4 Objectives and significance of the study

4.1 Objectives of the study

The objectives of this study are:

(1) To evaluate the efficacy and safety of secondary endocrine therapy in general as well as that of specific drugs in estrogen receptor-positive, HER2-negative postmenopausal metastatic breast cancer for which primary endocrine therapy had no favorable clinical effect (low sensitivity to primary endocrine therapy).

(2) To clarify the effect of reactivity to the previous endocrine therapy (the period from the start of postoperative endocrine therapy to recurrence) and the biological characteristics of any given tumor (expression intensity of ER and presence or absence of PgR expression) on the results of the secondary endocrine therapy, and to obtain information to complement Hortobagyi's therapeutic algorithm.

4.2 Background inspiring this study

(1) Target disease

According to the National Cancer Center, Center for Cancer Control and Information Services, the estimated annual number of Japanese females affected with breast cancer in 2011 was about 72,500, and the rate per 100,000 population was 110.5, showing that the breast was the organ most frequently affected with malignancy in females. The annual number of deaths due to breast cancer in Japan was 13,148 in 2013, which is the 5th leading cause of mortality following stomach, colorectal, lung, and pancreatic cancers.
The incidence of breast cancer in Japanese females is constantly increasing and ranks closely with colorectal cancer and lung cancer [4)].

About 90% of patients who are diagnosed with breast cancer receive surgical excision of the primary focus; however, the remaining 10% of patients have obvious distant metastasis at the first visit.

The survival rate of breast cancer is good if the tumor is detected early and removed surgically. It is considered that the presence or absence of micrometastasis, which is clinically undetectable at the initial visit, has a decisive influence on the patient's prognosis. About 60% of patients who received surgery were cured, on the other hand, about 40% experienced a relapse [5)].

The median prognosis for the life expectancy of patients whose breast cancer has developed metastasis and recurrence is 28 months, and achieving a cure of metastatic or recurrent breast cancer is difficult even with current treatment techniques.

(2) Treatment of target population

① Goal of treatment for metastatic breast cancer

It is very rare to achieve a complete cure for breast cancer that is already associated with

inoperable distant metastasis at the first visit (Stage IV) and recurrent breast cancer caused by distant metastasis; therefore, the major purposes of treatment are alleviation of symptoms, life extension, and maintenance and improvement of quality of life (QoL) in such cases [6, 7]. Metastatic breast cancer is mainly treated by drug therapy. Radiotherapy, surgical therapy, etc. are also used in combination as needed. These treatments are intended to provide alleviation of symptoms and maintenance of daily life.

② Endocrine therapy for metastatic breast cancer

The selection of therapies based on the biological characteristics of individual cancers has been emphasized in the medical treatment of breast cancer in recent years. Breast cancer has several subtypes, which are determined based on the expression of estrogen receptor (ER) and human epidermal growth factor receptor type2 (HER2) by the tumor, which are important prognosis predictors as well as effect predictors for endocrine therapy, chemotherapy, and molecularly-targeted agents; therefore, selection of therapy based on the subtype classification is implemented.

Furthermore, an algorithm proposed by Hortobagyi is implemented in clinical practice as a treatment strategy. For ER-positive metastatic breast cancer, treatment usually begins with an endocrine therapy and moves to different treatments as soon as the first therapy loses its efficacy, if there is no distant metastasis threatening the life prognosis. For cases responsive to the primary endocrine therapy, it is fundamental to continue endocrine therapy because the antitumor effect of the secondary endocrine therapy can be still expected even if the primary endocrine therapy loses its efficacy [8].

③ Recent outlooks regarding sensitivity and tolerance to endocrine therapy

Recently, several classification models regarding sensitivity and tolerance to endocrine therapies have been proposed as the basis of the clinical course associated with initial endocrine therapy.

The 2nd International Consensus Guidelines for Advanced Breast Cancer (ABC2), which was held as an international consensus conference for recurrent breast cancer, proposed classifications of resistance to endocrine therapies for ER-positive metastatic breast cancer according to the period from the start of initial endocrine therapy to recurrence or progression as below [9]:

[Primary endocrine therapy resistant breast cancer]

- Cases that showed recurrence within 2 years after the initiation of postoperative adjunct endocrine therapy.
- Cases that showed progression of the disease condition within 6 months after the start of initial endocrine therapy for metastatic breast cancer.

[Secondary endocrine therapy resistant breast cancer]

- Cases that showed recurrence within 2 years after the initiation of postoperative adjunct endocrine therapy or within 12 months after the completion of the endocrine therapy.
- Cases that showed progression of the disease condition within 6 months after the start of initial endocrine therapy for metastatic breast cancer.

In addition, a classification scheme based on drug sensitivity that categorizes cases that show recurrence within 2 years after the initiation of postoperative adjunct endocrine therapy and cases that show progression within 3 months after the start of initial endocrine therapy as "very low" drug sensitivity, and cases that show recurrence after 2 years from the initiation of postoperative adjunct endocrine therapy as "low" drug sensitivity has also been proposed (Fig. 1) [9].

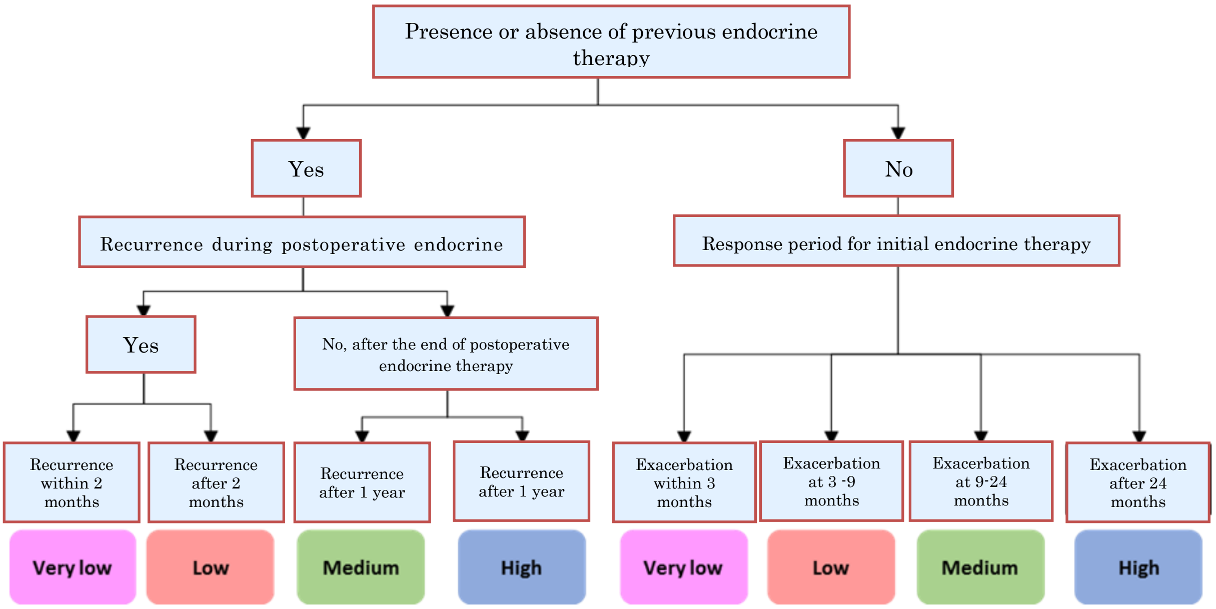

Figure 1: Classification of recurrent breast cancer according to its sensitivity to endocrine drug treatment as proposed during the ABC2 consensus conference

④ Report related to the efficacy of recent endocrine therapies

Recently, drug treatments for breast cancer that have a mechanism of action different from that of existing drugs became available.

Fulvestrant has no partial agonistic effect on ER such as that observed for tamoxifen, but instead down regulates ER expression in breast cancer cells; therefore, it is classified as a selective estrogen receptor down regulator (SERD) [10-12].

Based on the results of a Phase II comparative study that compared anastrozole and

fulvestrant as primary endocrine therapies in metastatic breast cancer (First-Line Study Comparing Endocrine Treatments [FIRST] trial), fulvestrant has clinical efficacy equivalent to anastrozole against metastatic breast cancer [13)].

In addition, everolimus is a mammalian target of rapamycin (mTOR) inhibitor. Inhibition of mTOR, which is situated downstream of the PI3K/AKT pathway, a key signaling pathway controlling cell proliferation, reveals a cancer cell proliferation effect, and it was shown that PFS was extended by the combined administration of other endocrine therapies in breast cancer that became endocrine therapy resistant [14-16)].

It is expected that a clinically significant treatment effect can be obtained by secondary endocrine therapy, even for cases that have poor responsiveness to primary endocrine therapy, by the use of these drugs.

(3) Study plan

Validation of a randomized controlled study implemented in cases that showed poor responsiveness to the primary endocrine therapy is required to establish the standard treatment for such cases in the future. However, no previous clinical trials have evaluated the efficacy and safety of secondary endocrine therapies in cases that showed poor responsiveness to the primary endocrine therapy. Furthermore, currently there is a wide range of medicines available for use as secondary endocrine therapeutic medicines for breast cancer.

Fundamental information, such as the selection of the target population indicated for treatment with secondary endocrine therapy and the basis underlying the selection of the target population, and the treatment effect expected for the overall secondary endocrine therapy and for each individual medicine are required to plan a comparative clinical trial involving these medicines in the future.

Based on the above background, this observational study was planned.

This study aims to clarify the efficacy and safety of secondary endocrine therapy using endocrine therapeutic medicines in estrogen receptor-positive, HER2-negative postmenopausal metastatic breast cancer for which the primary endocrine therapy had no favorable clinical effect through prospective observation using outcome indices including the clinical benefit rate, progression free survival, overall survival, time to treatment failure, time to chemotherapy, response rate, health-related quality of life, and adverse events.

Secondary aims include to categorize the wide variety of endocrine therapeutic medicines that are currently usable in Japan based on their action mechanisms and to evaluate their efficacy and safety.

Cases that showed low sensitivity to previous endocrine therapy are the target population in this study, and it is expected that the response of these cases to secondary endocrine therapy will not

be uniform. As mentioned above, a very important issue is whether each case should be selected for secondary endocrine therapy or chemotherapy, in addition to the selection of the specific secondary endocrine medicine. The examination of efficacy predictors that may have an impact on responsiveness to the secondary endocrine therapy is also an objective of this study. Efficacy predictors examined in this study include ① responsiveness to previous endocrine therapy (time from the initiation of postoperative endocrine therapy to recurrence, or PFS of the primary endocrine therapy for metastatic breast cancer), and ② the biological characteristics of the tumor (intensity of ER expression and presence or absence of PgR expression).

4.3 Study hypotheses

To achieve the objectives, the following study hypotheses will be tested in this study.

(1) A clinical benefit rate of at least 30% can be expected for the latest endocrine therapeutic medicines for breast cancer with low sensitivity to the primary endocrine therapy.

(2) The effect of the secondary endocrine therapy can be predicted because responsiveness to previous treatment and the biological characteristics of tumor influence the therapeutic effect of the secondary endocrine therapy on breast cancer with low sensitivity to the primary endocrine therapy.

4.4 Significance of the study

About 70% of breast cancer cases are ER-positive. Accordingly, the proportion of ER-positive breast cancer is also high among metastatic breast cancers. Endocrine therapy chosen by following Hortobagyi's algorithm is implemented on a daily basis. However, there is no existing evidence base to allow the determination of the optimum secondary therapies for breast cancer with low sensitivity to the primary endocrine therapy. Clarification of the efficacy and safety of secondary endocrine therapy for breast cancer with low sensitivity to primary endocrine therapy in this study can provide valuable information to enable the evidence-based selection of appropriate secondary endocrine therapies in the future. Furthermore, this study clarifies clinical issues to be resolved and provides valuable information that can form a foundation for planning future clinical research. To date, no clinical studies have been conducted in breast cancer with low sensitivity to the primary endocrine therapy. Hence, this study has great significance in terms of its originality.

5 Methods and duration of the study

5.1 Study design

The planning and implementation of this study comply with "Ethical Guidelines for Medical and Health Research Involving Human Subjects" (Appendix A), which was published by the Ministry of Education, Culture, Sports, Science and Technology and the Ministry of Health, Labour and Welfare on December 22, 2014. According to our interpretations of the terms "intervention" and "invasion" described in those guidelines, this study corresponds to "non-intervention study involving mild invasiveness", and the study design is regarded as an observational study.

(1) Invasiveness

Invasiveness is defined as "To cause injuries or distress to a research subject's body and/or mind by conducting a procedure for investigational purpose, such as puncture, incision, administration of drugs, irradiation and questions related to the subject's mental trauma, etc. Of various types of invasiveness, one causing minor injury and/or distress on the research subject's body and/or mind is called 'minor invasiveness'." in Chapter 1, Part 2 "Glossary" (2) of the guidelines.

The treatment implemented in this study will be conducted as regular clinical practice based on the preferences of medical staff and patients using drugs covered by insurance. Therefore, this study does not correspond to the treatment defined in the "Objectives of the study".

According to the survey plan implemented in this study, evaluation of Quality of life (QoL) by questionnaire will be conducted. Investigation of QoL in medical studies is implemented using scales validated for relevance and reliability, and is generalized as an evaluation approach for medical research. Considerations of the mental or physical burdens to patients are included in the process of the validation of the scales (content validity). Therefore, it is considered that the evaluation of QoL produces no mental damage to the subjects. The amount of time required to complete a QoL evaluation is about 15 minutes each time, and a mild burden is produced due to implementing the evaluation a total of 3 times (at the time of registration and at 1 and 3 months). Based on the above, it is considered that the burden for the subjects participating in the study is small; therefore, the level of invasion produced for the subjects participating in this study corresponds to "minor invasiveness".

(2) Intervention

Intervention is defined as "A practice for investigational purpose to control the presence or absence of factors that can affect a variety of events related to human health (including activities to maintain and promote good health and medical practices such as medication and examinations for prevention, diagnosis, and treatment of the patients), or the degree of such factors. The above-defined intervention also includes medical techniques beyond usual medical practice that are conducted for investigational purposes." in Chapter 1, Part 2 "Glossary" (3) of the guidelines.

The treatment implemented in this study will be conducted as regular clinical practice based on

the preferences of medical staff and patients using drugs covered by insurance. Therefore, this study does not correspond to the treatment defined in the "Objectives of the study". A total of 3 imaging evaluations conducted at the time of registration and at 3 and 6 months are specified as investigations in this study. However, these evaluations do not correspond to medical practice exceeding the range of regular medical examination as an evaluation approach of drug therapy for metastatic breast cancer. In addition, evaluation of QoL does not correspond to medical practice. As mentioned above, this study is a medical research study involving no intervention and is therefore categorized as an observational study.

5.2 Case registration

(1) Completion of "Case registration card"

Clinical investigators will confirm that study subjects satisfy the inclusion criteria (6.2) and complete all question items of the Case registration card (Appendix B). Then, the cards will be sent to the data center by fax.

CSPOR Data Center, Japan Clinical Research Support Unit, NPO<br>FAX: 03-5298-8536, Phone: 03-3254-8029<br>Reception opening times: 10 am–5 pm, weekdays<br>(Closed on public holidays, Saturday, Sunday, and year-end and New Year holidays)

(2) Confirmation of registration

The data center will confirm the eligibility of the study subjects based on the received Case registration cards and will then register the subjects.

① When the description given on the Case registration cards is inadequate, the subject will not be registered.

② The date when a sequence of registration procedures is completed will be used as the registration date, and described in "Confirmation note for case registration" (Appendix C). The case will not be "registered" at the time of submission of the Case registration card to the data center by fax.

③ Registration of study subjects will not be canceled (i.e., deleted from the database) except when utilization of their data for research is denied. In cases of duplicated registration, the first registration information (earliest registry number) is adopted.

④ When a registration error or duplicated registration is found, a clinical investigator must immediately contact the datacenter.

(3) Sending of "Confirmation note for case registration"

The data center sends a "Confirmation note for case registration" via fax to the clinical investigators described in the Case registration card.

5.3 Treatment

(1) Selection of treatment

The objective of the study is to evaluate the efficacy and safety of general secondary endocrine therapies that are implemented in regular clinical practice for the target population. However, there are a broad range of different endocrine therapeutic medicines available for use in the target population selected in this study, and the optimum medicines for this population are not established. The treatment choices available for the target population include various medicines covered by insurance for use as endocrine therapeutic medicines for postmenopausal breast cancer in Japan, except for those endocrine therapeutic medicines used in a patient's previous treatment {see the table in section 5.3 (2)}.

Treatment will be selected based on discussion between medical staff and patients in this study (treatment of physician's choice: TPC). In addition, prohibition of concomitant therapy is not specified in this study because this study is an observational study. However, it is recommended to use medical agents with an antitumor effect (chemotherapeutic agent, concomitant use of molecular target drug, and other endocrine therapy) and to combine drug treatment with other treatment (surgery, radiotherapy) as appropriate in accordance with published guidelines [8). Furthermore, when these therapies are implemented in combination with the endocrine therapy, the details will be described in the patient's Progress report. Planned treatment shall be clearly indicated on the Case registration card when a subject participates in this study.

Hereinafter, preventive treatments used and evaluated for their efficacy and safety in the study populations are referred to as "protocol treatment". Protocol treatment shall be started within 2 weeks after registration. If treatment cannot be started within 2 weeks, the reasons for the delay are recorded in the "Progress report" (Appendix D).

See the package inserts of drugs used for information about the protocol treatment. The latest package inserts are available at the website "Pharmaceuticals and Medical Devices Information Service Home Page" (http://www.info.pmda.go.jp/).

(2) Endocrine therapeutic medicines

The drug name as well as the dosage and administration used, which are approved as endocrine therapeutic medicines for postmenopausal breast cancer in Japan, are shown in a table below.

Classification	Drug name	Dosage and administration
Selective estrogen receptor modulators (SERMs)	Tamoxifen	Usually, 20 mg daily is orally administered in divided doses of 1–2 doses. Dosage may be appropriately increased depending on the symptoms, but the daily maximum dosage of tamoxifen is 40 mg.
	Toremifene	Usually orally administered as 40 mg toremifene once daily in adults. Meanwhile, usually, orally administered as 120 mg toremifene once daily in adults for previously treated cases (cases that showed no response to medicinal treatment, radiotherapy, etc.). Dosage is appropriately increased depending on the symptoms.
Aromatase inhibitor (AI)	Anastrozole	Usually orally administered as 1 mg anastrozole once daily in adults.
	Letrozole	Usually orally administered as 2.5 mg letrozole once daily in adults.
	Exemestane	Usually orally administered after meals as 25 mg exemestane once daily in adults.
Selective estrogen receptor down regulator (SERD)	Fulvestrant	Usually, a gluteal injection of 500 mg fulvestrant is intramuscularly administered into each side once at the first administration, 2 weeks and 4 weeks later, and every 4 weeks after that in adults.
Progestational hormone agent	Medroxyprogesterone acetate	Usually, 600–1200 mg daily is orally administered as medroxyprogesterone acetate in divided doses of 3 doses in adults.

(3) Combination use of mammalian target of rapamycin (mTOR) inhibitor

Significant extension of PFS was reported in the everolimus + exemestane group as compared with the placebo + exemestane group in a Phase III global clinical trial conducted in ER-positive, HER2-negative, nonsteroidal aromatase inhibitors (letrozole or anastrozole) resistant, locally-advanced or metastatic, postmenopausal breast cancer patients (a double-blinded comparative study conducted in 24 countries throughout the world including Japan) [15, 16].

In addition, extensions of the time to significant progression and overall survival were observed in the everolimus + tamoxifen group as compared with the tamoxifen group in a Phase II randomized trial conducted in aromatase inhibitors resistant, locally advanced, or metastatic, postmenopausal breast cancer patients [17].

Administration of everolimus in combination with an endocrine therapeutic medicine for inoperable or recurrent breast cancer is allowed in Japan. Accordingly, combination use of the endocrine therapeutic medicines listed in 5.3 (2) with everolimus is considered as a therapeutic option in this study. Combination use of everolimus and endocrine therapeutic medicines is considered as a factor that may affect efficacy and adverse events; therefore, this is described as a

concomitant drug in the Progress report.

(4) Categorization of drug types used for the analysis

Currently, the first-line drugs used for postoperative endocrine therapy and primary endocrine therapy for metastatic breast cancer are aromatase inhibitors. It is predicted that most of the cases registered in this study received administration of aromatase inhibitor as a pretreatment. There are a number of aromatase inhibitors that are available for use as secondary therapies. These therapies can be categorized based on their principal mechanism of action as below. Their efficacy and safety will be evaluated using the following categories including the general secondary endocrine therapy in this study. In addition, the subject group categorized into "Others" below will not be subjected to the analysis because the interpretation of the analysis results would be too difficult.

- Cohort SERMs

  Selective estrogen receptor modulators (SERMs): When tamoxifen or toremifene, which are SERMs, is selected.

- Cohort AIs

  Aromatase inhibitor: When anastrozole, letrozole, or exemestane, which are AIs, is selected.

- Cohort SERD

  Selective estrogen receptor down regulator (SERD): When fulvestrant, which is a SERD, is selected.

- Cohort with mTORi

  When the combined use of an endocrine therapeutic medicine and everolimus, which is an mTOR inhibitor, is selected. Cases that used everolimus concomitantly are categorized into this cohort regardless of the type of endocrine therapeutic medicine concomitantly used.

- Others

  When the selected therapy cannot be categorized into any of the above cohorts.

(5) Concomitant treatments and medicines, and terms to be described in the Progress report

① Treatment in accordance with guidelines

Prohibition of concomitant treatments and medicines associated with participation in this study is not specified, but it is recommended to implement standard treatment in accordance with the published guidelines [8)].

When the following treatments or medicines are concomitantly used with the protocol

treatment, which was preliminarily reported after the registration, the reasons for the implementation (the presence or absence of withdrawal due to exacerbation of the disease or adverse events caused by the protocol treatment) must be described in the Progress report.

A) Surgical therapy for breast cancer focus
B) Radiotherapy for breast cancer focus
C) Molecularly-targeted drugs other than mTOR inhibitors
D) Chemotherapy
E) Immunotherapy
F) Other use of medicines with known antitumor activity against breast cancer

② Other treatments and medicines requiring description in the Progress report

The presence or absence of combination therapy must be described for the following medicines that are often concomitantly used as therapeutic medicines for metastatic breast cancer. A description of the reasons for implementation is not required.

G) Use of bisphosphonate formulation aimed at treatment of bony metastasis
H) Use of anti-RANKL antibody aimed at treatment of bony metastasis

③ Treatments and medicines not requiring description in the Progress report

The following concomitant medicines and treatments do not require description in the Progress report.

I) Therapeutic medicines for any preexisting disorder
J) Therapeutic medicines aimed at alleviation of symptoms and treatment of adverse events

(6) Selection of treatment after the end of protocol treatment and terms to be described in the Progress report

Selection of treatment after the end of protocol treatment is not specified. Since an event occurring before the date of the initiation of chemotherapy for metastatic breast cancer is determined as an outcome measures in this study, the starting date of administration and type of chemotherapy administered will be inquired and described in the Progress report when chemotherapy is implemented after the end of the protocol treatment.

5.4 Survey

(1) Survey items

The following survey and examination will be implemented to evaluate the efficacy and safety of the protocol treatment for subjects in this study.

① Medical history

② Physical findings

③ Diagnostic imaging for evaluation of breast cancer focus

④ Survey of adverse events

⑤ Treatment survey: the name of the selected medicine and the reasons for the selection, compliance, combined medicine, and treatment

⑥ Survey of health-related quality of life (HRQoL)

⑦ Survey of aftertreatment

⑧ Prognosis survey

The following section, 5.4 (2), specifies the guidelines for conducting surveys ①–⑤ within the range of regular clinical practice while paying careful attention to the burden imposed on patients in this study. In addition, survey of HRQoL will be implemented with attention to the burden imposed on the study subjects. Usually, the time for each survey is limited to 15 minutes and the number of times the survey is carried out is limited to the requisite minimum (3 times in total including at the time of registration, and 1 and 3 months after the initiation of protocol treatment). Surveys at 6 months after the initiation of protocol treatment and later will not be implemented except for the surveys of aftertreatment and prognosis.

(2) Survey schedule

The surveys will be implemented on the schedule shown in the table below

Survey term	Before registration	1 month after the initiation of protocol treatment	3 months after the initiation of protocol treatment	6 months after the initiation of protocol treatment
Medical history	○			
Physical findings Note 1<br>Height, weight, performance status	○		○	○
Image assessment Note 2	○Note 3		○Note 4	○Note 4
Adverse event	○		○	○
Treatment survey	○		○	○
HRQoL	○	○ Note 4	○Note 4	
Survey of aftertreatment		Survey is implemented after discontinuation of the protocol treatment Note 5		
Prognosis survey				

Note 1: Measurement of body weight will be performed only at the time of registration.

Note 2: In principle, image assessment should be implemented by the same technique as that implemented before registration.

Note 3: Evaluation will be implemented within 28 days (within 4 weeks) before registration

Note 4: Evaluation within 2 weeks before or after the specified day is recommended.

Note 5: Every year after the end of the protocol treatment.

(3) Survey required before the registration

Clinical investigators implement the following survey before registration of the case.

① Medical history

A) Terms related to the primary lesion

- Presence or absence of surgical history and date of operation
- Classification of clinical stages (see 3.3 Classification of clinical stages)
- Histological type (see 3.4 Histological classification)
- Number of histological lymph node metastases
- Expression of hormonal receptors (ER and PgR)
- Expression status of HER2

B) History of pretreatment

- Presence/absence of implementation, type, and date of final administration of preoperative/postoperative chemotherapy
- Type, date of initiation, and date of final administration of postoperative endocrine therapy; or type, date of initiation, and date of final administration of the primary endocrine therapy
- Date of confirmed recurrence or date of confirmed progression
- Presence/absence of a history of radiotherapy after recurrence and date of final radiation treatment
- Presence/absence of a history of chemotherapy after recurrence (confirmation of absence)
- Organs confirmed the presence of recurrence or metastasis

C) Past medical history

② Physical findings

A) Height

B) Weight

C) Performance Status

③ Diagnostic imaging for evaluation of breast cancer lesions

The following terms will be evaluated within 28 days (within 4 weeks) before registration.

A) Chest CT, MRI, or chest X-ray (essential)

B) Abdominal CT, MRI, or abdominal ultrasound (essential)

C) Bone scintigraphy when bone metastasis is clinically suspected

* When bone metastasis is suspected by bone scintigraphy, bone radiography, CT or MRI, etc. is conducted to evaluate the lesion.

D) Brain CT or MRI when clinically brain metastasis is clinically suspected.

E) Evaluation and recording of lymph node and local skin recurrent focus

* Inspection: It is favorable to observe progress using photos with indicators attached.

* Palpation: The size of foci such as superficial lymph nodes and cutaneous metastases are directly measured from outside the body if the size is measurable. It is desirable to evaluate with CT and/or ultrasound when the measurement method is applicable.

④ Survey of adverse events

Adverse events of Grade 3 or higher will be confirmed in accordance with the Common Terminology Criteria for Adverse Events v4.0 Japanese translation JCOG edition, and recorded.

Especially, the presence or absence of the following adverse events, which are common symptoms in breast cancer patients and associated with endocrine therapy, will be confirmed and recorded.

When adverse events of Grade 3 or higher are confirmed for symptoms other than the followings, the symptoms as recorded as "Other adverse events".

- Constipation
- Diarrhea
- Oral mucositis
- Nausea
- Vomiting
- Malaise
- Pain
- Joint pain
- Insomnia
- Vaginal discharge
- Vaginal dryness
- Hot flash

⑤ Survey of treatment

Presence or absence of concomitant medications (use of bisphosphonate formulation or anti-

RANKL antibody), endocrine therapeutic medicines planned to be administered, scheduled day of initiation of treatment, and reasons for the selection.

⑥ Survey of HRQoL (See details for 5.5 Survey of health related quality of life)
The following questionnaire (scale) will be used to evaluate HRQoL.
It is desirable that evaluation of HRQoL is completed by the date of registration. However, if it is not completed because of a lack of time or other reasons, the questionnaire should be completed by the patient the day before the initiation of the protocol treatment.

A) Social background: Academic background, situation regarding employment, household income, presence or absence of partner
B) Expectancy of HRQoL
C) FACT (Functional Assessment of Cancer Therapy): ES (endocrine symptoms, 18 items) and B (breast cancer, 9 items) as additional QOL scales and FACT-G (General, 29 items, Japanese version), which is generally used for clinical studies of cancer.

(4) **Survey plan at 1 month after the initiation of protocol treatment**

The following survey will be implemented at 1 month after the initiation day of protocol treatment:

① HRQoL survey (See details for 5.5 Survey of health related quality of life)
The following survey will be implemented at 1 month after the initiation of protocol treatment:
<u>* It is acceptable to implement the survey within 2 weeks before or after the specified day.</u>

A) FACT (ES and B)
B) Questions about changes to identify minimally important differences

(5) **Survey planned at 3 months after the initiation of protocol treatment**

The following survey will be implemented at 3 months after the initiation date of protocol treatment:

① Physical findings

* It is acceptable to implement the survey within 2 weeks before or after the specified day.

A) Weight

B) Performance status

② Diagnostic imaging for evaluation of breast cancer lesions

* It is desirable to use the same techniques as those used for the examination implemented before registration.

* It is recommended to implement the examination within 2 weeks before or after the specified day.

A) Chest CT, MRI, or chest X-ray

B) Abdominal CT, MRI, or abdominal ultrasound

C) Bone scintigraphy when bone metastasis is clinically suspected

* When bone metastasis is suspected by bone scintigraphy, bone radiography, CT, or MRI, etc. is conducted to further evaluate the lesion.

D) Brain CT or MRI when brain metastasis is clinically suspected

E) Evaluation and recording of lymph nodes and locally recurrent skin foci

* Inspection: It is favorable to record progress using photographs with indicators attached.

* Palpation: The size of foci such as superficial lymph nodes and cutaneous metastases will be directly measured from outside the body if their sizes are measurable. It is desirable to use CT and/or ultrasound for evaluations where applicable.

③ Survey of adverse events

The presence or absence of adverse events of Grade 3 or higher occurring within the past 3 months will be confirmed and recorded.

* It is acceptable to implement the survey within 2 weeks before or after the specified day.

The contents of survey are the same as specified in ④ of 5.4 (3). The survey must be completed before registration.

④ Survey of treatment state

A) Presence or absence of discontinuation of the protocol treatment and the reasons and date of discontinuation if discontinued

B) Compliance with the protocol treatment for the past 3 months

C) Presence or absence of use of bisphosphonate formulation or anti-RANKL antibody for the past 3 months

D) Presence or absence and reasons for implementation of concomitantly used therapies (surgical therapy for breast cancer focus, radiotherapy for breast cancer focus, any molecularly-targeted drugs other than mTOR inhibitors, chemotherapy, immunotherapy, other drugs recognized to have antitumor activity against breast cancer) for the past 3 months

⑤ Survey of HRQoL {the same as 5.4 (4) ①}

(6) Survey plan at 6 months after the initiation of protocol treatment

The following survey will be implemented at 6 months after the initiation day of protocol treatment:

* However, the survey at 6 months after the initiation is not required when discontinuation of the protocol treatment or progression is reported in the survey at 3 months after the initiation. Move to the survey detailed in (7) for such cases.

① Physical findings

* It is acceptable to implement the survey within 2 weeks before or after the specified day.

A) Weight

B) Performance status

② Diagnostic imaging for evaluation of breast cancer lesions

* It is desirable to use the same techniques as those used for the examination implemented before registration.

* It is recommended to implement the examination within 2 weeks before or after the specified day.

A) Chest CT, MRI, or chest X-ray

B) Abdominal CT, MRI, or abdominal ultrasound

C) Bone scintigraphy when bone metastasis is clinically suspected

* When bone metastasis is suspected by bone scintigraphy, bone radiography, CT, or MRI, etc. will be conducted to further evaluate the lesion.

D) Brain CT or MRI when brain metastasis is clinically suspected

E) Evaluation and recording of lymph nodes and locally recurrent skin foci

* Inspection: It is favorable to record progress using photographs with indicators attached.

* Palpation: The size of foci such as superficial lymph nodes and cutaneous metastases will be directly measured from outside the body if their sizes are measurable. It is

desirable to use CT and/or ultrasound for evaluations where applicable.

③ Survey of adverse events

The presence or absence of adverse events of Grade 3 or higher occurring between the previous survey and the day of the current survey is confirmed and recorded.

* It is acceptable to implement the survey within 2 weeks before or after the specified day.

The contents of survey are the same as specified in ④ of 5.4 (3). The survey must be completed before registration.

④ Survey of treatment state

A) Presence or absence of discontinuation of the protocol treatment and the reasons and date of discontinuation if discontinued

B) Compliance with the protocol treatment for the past 3 months

C) Presence or absence of use of bisphosphonate formulation or anti-RANKL antibody for the past 3 months

D) Presence or absence and reasons for implementation of concomitantly used therapies (surgical therapy for breast cancer focus, radiotherapy for breast cancer focus, molecularly-targeted drugs other than mTOR inhibitors, chemotherapy, immunotherapy, other drugs recognized to have antitumor activity against breast cancer) for the past 3 months

(7) Survey plan at 6 months or later after the initiation of protocol treatment or after discontinuation of the protocol treatment

① Timing of survey

A) Every year from 6 months after the initiation of the protocol treatment when the protocol treatment is ongoing at the time of 6 months after the initiation of the protocol treatment

B) Every year from the day of discontinuation of the protocol treatment when the protocol treatment is discontinued at the time of 6 months after the initiation of the protocol treatment

② Contents of survey

A) Continuation state of protocol treatment: Presence or absence of continuation of the protocol treatment. If it is discontinued, the date and reasons for the discontinuation {only for subjects corresponding to the criteria specified in 5.4 (7) ①-A)}.

B) Survey of progression: Presence or absence of progression defined in 3.9 (2). When progression was observed, the date when the progression occurred was determined (not required for a subject whose progression is already reported.)

C) Survey of after-treatment: Presence or absence of implementation of chemotherapy. If chemotherapy is implemented, the date and type of chemotherapy.

D) Survey of prognosis: The final confirmed day of survival. In case of death, the date and cause of death.

(8) Discontinuation of survey

When a case meets one of the following criteria, the survey will be discontinued.

① When it is determined that continuation of the rest of the survey is not possible due to changing hospital or other reasons

② Withdrawal of consent

③ Death

④ When it becomes clear that the eligibility criteria are not satisfied

⑤ Other reasons, when it is determined that continuation of the study as a subject is not possible

5.5 Survey of health-related quality of life

(1) Background of HRQoL evaluation

Health-related quality of life (HRQoL) is generally defined as the effect of diseases and treatment on health and the entirety of life [18]. Usually HRQoL is a multidisciplinary concept consisting of basic domains including functional, physical, mental, and social aspects and emphasizes the subjective experience of the patient as the central point of evaluation. Recently, it has become clear that there can be a great dissociation between subjective evaluation by medical staff and that by patients even for single areas such as individual symptoms or signs. As a result, HRQoL emphasizes the subjective experience of patients even for evaluations of single areas [19]. In the context of that background, the concept of patient-reported outcomes (PROs) as a more comprehensive subjective evaluation framework including HRQoL and symptoms in the area of health was established. Furthermore, various scales that have been validated for their reliability and validity for measuring PROs have been developed following the establishment of the concept of PROs. In 2012, the American Society of Clinical Oncology (ASCO) published "Recommendations for incorporating patient-reported outcomes into clinical comparative effectiveness research in adult oncology", and stated that the evaluation of PROs is essential to obtain information useful for making decisions about a patient in clinical comparative studies of adult cancer, especially those of intractable metastatic cancer [20]. Metastatic breast cancer, which is the subject of this study, is intractable even using current medical treatment, and it is considered that the main purposes of treatment in such cases are the extension of survival time, maintenance and improvement of HRQoL, and alleviation of symptoms [21].

(2) Objectives of HRQoL evaluation

The major objective of this study is to evaluate the efficacy and safety of recent endocrine therapies for breast cancer with low sensitivity to the primary endocrine therapy. The maintenance and improvement of QoL is a key objective in the treatment of metastatic breast cancer, and evaluation of QoL is essential for the evaluation of the efficacy of treatments. HRQoL is often discussed as differences and changes in scores obtained by the statistical analysis of questionnaire data. However, it is critical to understand the clinical significance of any differences and changes in such scores. Recently, the concept of minimally important difference (MID) has been emphasized as an index to understand HRQoL scores. MID represents the clinically meaningful minimum difference in the HRQoL scores, and validation of MID is indispensable for the meaningful evaluation of HRQoL in clinical cancer research.

As mentioned above, objectives of the HRQoL evaluation in this study are as below:

① To clarify the effect of secondary endocrine therapy on estrogen receptor-positive, HER2-negative postmenopausal metastatic breast cancer for which the primary endocrine therapy had no favorable clinical effect (low sensitivity to primary endocrine therapy) on HRQoL using HRQoL maintenance rate, which is a clinically meaningful index.

② To clarify the clinical significance inherent in HRQoL score values as the minimally important differences (MIDs) associated with endocrine therapy for metastatic breast cancer by focusing attention on variations in HRQoL scores obtained for subjects of this study over time.

(3) HRQoL scales

The following scales are used (Appendix F).

In addition, a questionnaire survey related to the social background and expectancy of QoL of patients, which may have an impact on subsequent HRQoL scores, will be conducted at the time of registration.

① (Functional Assessment of Cancer Therapy) ES (Endocrine Symptoms, 19 items) and B (Breast, 10 items) [22-24] as additional QOL scales, FACT-G (General, 27 items, Japanese version), which is a scale generally used for clinical studies of cancer.

② Questions about changes: Used as subjective anchoring information required for identification of MIDs (minimally important differences).

(4) Survey schedule

Survey term	At registration Note 1	1 month after the initiation of protocol treatment Note 2	3 months after the initiation of protocol treatment Note 2
Social background	○		
Expectancy of QoL	○		
FACT-G, -B, -ES	○	○	○
Questions about changes		○	○
Survey of medical staff about the reasons for missing data	When the questionnaire is not sent back from the subjected patient after the scheduled survey period.		

Note 1: It is desirable to complete the survey at the time of registration between the acquisition of consent and the completion of registration.

When there is insufficient time, the survey should be completed before the initiation of the protocol treatment.

Note 2: It is acceptable to implement the survey within 2 weeks before or after the specified day.

(5) HRQoL survey for patients who discontinued the protocol treatment

Evaluation of HRQoL will be discontinued when the protocol treatment is discontinued due to disease progress, development or exacerbation of complications, or adverse events.

(6) Survey method

- ECOG PS will be determined by a clinical investigator at the same time as the QoL survey is conducted using specified survey terms and the results will be described in the Progress report.
- When the patient cannot complete the questionnaire due to an exacerbation of the disease condition of the patient, it is acceptable to implement the survey by reading the questionnaire by the Clinical Research Coordinator (CRC).

(7) Survey of reasons for missing data

When the questionnaire is not sent back for a patient after the scheduled survey period (5.5 (4)), the data center surveys the reasons for the missing data, including the situation of questionnaire distribution and the health condition of the subject by asking the responsible clinical investigator. The reasons for missing data are classified as below, and described as profiles of the study subject in the statistical analysis report and publications of the study results (conference presentations and paper publications).

① Exacerbation of disease condition of the subject
② Death of the subject
③ Rejection of the survey by the subject
④ Forgetfulness of the subject in completing and returning the questionnaire or loss of the questionnaire
⑤ Reasons associated with factors caused by the subject other than the above
⑥ Forgetfulness of distribution of the questionnaire from a person in charge to the subject

(8) Number of patients scheduled to undergo HRQoL survey

All of the subjects registered into this study are subjects of the survey.

5.6 Submission of data

The Case registration card for each subject will be submitted by fax, while other case report forms are submitted to the CSPOR data center via an electronic data capture (EDC) system in this study.

Clinical investigators, clinical research coordinators, and others will submit other case report forms to the CSPOR data center for all patients registered into this study on an ongoing basis until the completion of the study. When the Clinical Research Coordinator or another person completes the case report forms, verification by a clinical investigator is required.

Types of submitted case report forms and the method and timing of distribution/submission are shown below:

No.	Type	Method and timing of distribution	Method and timing of submission
1	Case registration card	Sent to participating facilities in advance.	Fax<br>At the time of registration, within 2 weeks before the scheduled initiation of protocol treatment.
2	Report of initiation of protocol treatment	EDC	EDC<br>Submitted within 2 weeks after the initiation of protocol treatment.
3	Progress report<br>(Adverse events, effect measurement)	EDC	EDC<br>Submitted within 2 months from each specified date of the 3- and 6-month surveys.
4	Progress report<br>(After-treatment)	EDC	EDC<br>Submitted within 2 months from the specified date of the annual survey after the completion of the protocol treatment.
5	Progress report<br>(Survival)	EDC	EDC<br>Submitted within 2 months from the specified date of the annual survey after the completion of the protocol treatment.
6	Emergency adverse event report	A form provided by the hospital is also acceptable.<br>Sent to participating facilities in advance.	Fax<br>Within 72 hours after having cognizance of the incidence.

5.7 Items described in reports

(1) Case registration card

① Date of entry, name of facility, name of clinical investigator, patient identification number, phone number of facility, and fax number for response

② Age, height, and weight

③ Expression status of ER and PgR (J-score classification, at least 0%, 1–9%, and 10% or higher)

④ Inclusion criterion (Yes/no; the date of acquisition of consent; 0, 1, or 2 is selected for PS)

⑤ Exclusion criterion (Yes/no; confirm that all terms do not correspond)

⑥ Planned treatment and scheduled initiation date

(2) Report of initiation of protocol treatment

① Medical history: (Of terms in 5.3.2 (1), all items except expression of hormonal receptors and expression status of HER2)

② Implementation state of imaging examinations before the registration: 5.3.2 (3)

③ Focal findings: Presence or absence of nonmeasurable lesions, descriptions of the site and measurement results of target lesions and non-target lesions.

④ Adverse events

⑤ Concomitant drugs

⑥ Started drugs, reasons for drug selection, and date of initiation

(3) Progress report: During protocol treatment

At 3 and 6 months after the initiation of protocol treatment unless discontinued.

No report at 6 months is required when discontinuation of the protocol treatment is reported in the survey at 3 months.

① Body weight, PS

② Implementation state of image examinations after the initiation of protocol treatment: 5.3.4 (2) and 5.3.5 (2)

③ Focal findings: Descriptions of the site and measurement results for target lesions and non-target lesions.

④ Adverse events

⑤ Concomitant drugs and treatment, and reasons for implementation

⑥ Presence or absence of discontinuation of the protocol treatment. When the treatment is discontinued, the reasons for the discontinuation and date of discontinuation.

⑦ Compliance of the protocol treatment

⑧ Time point response

(4) Progress report: After-treatment

① After-treatment survey: Implementation date and type of chemotherapy

(5) Progress report: Prognosis survey

① Presence or absence of progression and date of confirmation

② Date of last confirmation of survival, date and cause of death (in case of death)

(6) Emergency adverse event report

(See Appendix E)

5.8 Handling of data

The CSPOR data center will implement chase-up of unsubmitted data, careful examination and inquiry of submitted data, data correction based on results of the inquiry, and database management in accordance with data management plans (Standard Operating Procedure and Manual) specified separately. The CSPOR data center will prepare monitoring materials and data for statistical analysis based on the entered data.

5.9 Estimated enrollment and estimated study duration

(1) Estimated enrollment

200 or more (however, registration is to be continued during the registration period even if the target number is achieved)

(2) Estimetd study duration

Study duration: Four years from November 2015 to October 2019

Of this period, the registration period is a year from November 2015 to October 2016, and the observation period is November 2016 to 3 years from registration of the last case

5.10 Method of analysis and assay

(1) Definition of population to be analyzed

The analysis set is defined as below:

Total eligible cases: "Total eligible cases" is defined as a group of patients that overlapped and error registrations are removed from patients who registered in accordance with "5.2 Registration of cases".

Total treated cases: "Total treated cases" is defined as a subgroup of patients among the total eligible cases who received scheduled treatment at least once daily.

(2) Outcome measures

Outcome measures in this study are defined as below:

① Primary outcome measures

- Clinical benefit rate (CBR)

② Secondary outcome measures

- Progression free survival (PFS)
- Overall survival (OS)
- Time to treatment failure (TTF)
- Time to chemotherapy (TTC)
- Response rate (RR)
- Health-related quality of life
- Adverse events (toxicity)

(3) Analysis plan

① Primary analyses

Whether CBR, the primary outcome measures, exceeds 30% will be examined using accurate tests based on binomial distribution and the corresponding 90% confidence interval by cohorts with the entire secondary endocrine therapy. Meanwhile, the analysis set includes all treated cases, and a significance level of 5% is set for 1-sided tests.

② Other analyses

Analysis of the interaction effect of CBR is conducted for responsiveness to the primary endocrine therapy, expression intensity of ER, and presence or absence of PgR expression for all registered cases to examine the significance of effect predictors of the secondary endocrine therapy. The following classification will be used for each factor:

A) Responsiveness to the primary endocrine therapy

- Very low sensitivity group: Recurrence at less than 2 years after the initiation of postoperative endocrine therapy, or progression at less than 3 months after the primary therapy for metastatic breast cancer [corresponding to the 'very low' group of Figure 1 in 4.2 (2)].
- Low sensitivity group: Recurrence at 2–5 years after the initiation of postoperative endocrine therapy, or progression at 3–9 months after the primary therapy for metastatic breast cancer [corresponding to the 'low' group of Figure 1 in 4.2 (2)].

B) ER expression intensity

- ER low group: ER slightly positive (J-score classification is 1–9%)

- ER high group: ER positive (J-score classification is 10% or higher)

C) Presence or absence of PgR expression

- PgR negative group: PgR negative (J-score classification is negative)
- PgR positive group: PgR positive (J-score classification is 1% or higher)

Analyses of secondary outcomes will also conducted on a by-cohort basis. For RR defined by a 2-value endpoint response, an accurate 90% confidence interval based on point estimation and the binomial distribution is calculated. Furthermore, for PFS, OS, TTF, and TTC, which are time-to-event outcomes, a survival curve will be estimated using a Kaplan-Meier method, and 90% confidence intervals of the survival rate at each event will be calculated using Greenwood's formula. The interaction effect will be analyzed for effect predictors in the same manner as that used for the primary outcomes.

Analysis of adverse events will be performed in all treated cases. Incidence rate by types and grade will be calculated for reported adverse events.

③ Analysis of HRQoL

FACT-G, Breast, and ES, which are calculated as total scores, will be calculated for the aggregate score and subdomain scores in accordance with a scoring manual to calculate the fundamental statistics at the time of each survey for all subjects.

Furthermore, cases with decreases in scores exceeding the threshold values as compared with scores at the registration will be individually identified and the maintenance rate of QoL will be calculated using MIDs identified by scales as a meaningful threshold value of decrease of QoL score at each measurement point.

Information obtained from questions about changes will be used as subjective anchoring information required for the identification of MID during the endocrine therapy, and differences from the previously established MID will be examined.

Since it is considered that the evaluation and analysis of HRQoL have no influence on the clinical benefit rate, which is the primary outcome measures of this study, analysis will be implemented immediately after the data collection (after the collection of QoL questionnaires at 3 months for the last registered case). The results of the analysis will be published as conference presentations and papers.

6 Subject selection policy

6.1 Definition of the subject of this study

Although there is no existing consensus about how to define "ER-positive, HER2-negative postmenopausal metastatic breast cancer with low sensitivity to the primary endocrine therapy", a definition is established as below in this study:

- Cases that are continuing postoperative adjunct endocrine therapy and experienced recurrence within 5 years after the initiation of the therapy.
- Cases that experienced disease progression within 9 months after the initiation of the primary endocrine therapy for metastatic breast cancer.

These correspond to the "very low" and "low" groups described in Figure 1 in 4.2 (2).

Cases that fit the definition of the above-mentioned clinical course are called "breast cancer with low sensitivity to the primary endocrine therapy" in this study.

6.2 Inclusion criteria

Cases that satisfy all of the following conditions are included in this study as subjects.

(1) A patient with estrogen receptor (ER) positive [Note 2] postmenopausal [Note 3] breast cancer who is histologically diagnosed as breast cancer [Note 1].

Note 1: No distinction is made according to the tissue subtype of cancer.

Note 2: Determination of ER is based on the determination of the primary focus of the breast in accordance with the definition of ER positive that is detailed in section 3.6. In this regard, a case in which evaluation of the primary focus is difficult and which is determined as ER positive based on evaluation of a metastatic lesion can be registered. Furthermore, when the results of evaluation differ between the primary focus and metastatic lesion, the result of the primary focus will be considered for the evaluation of ER expression.

Note 3: At least 1 of the following conditions regarding menopause should be satisfied.

① Aged 60 years or older

② Younger than 60 years and absence of menstruation for the past 1 year or longer (without hysterectomy)

③ Had bilateral oophorectomy

When the presence or absence of menopause of the subject is unclear, FSH and plasma estradiol levels will be measured to confirm whether these are at the after menopausal level as defined by the criteria of each facility.

(2) Diagnosis of breast cancer corresponds to either of the following. No distinction is made according to the presence or absence of measurable lesions.

① Stage IV breast cancer associated with distant metastasis that cannot be a candidate for surgery at the first visit.
(See 3.3 Classification of clinical stages)

② Breast cancer associated with progression or recurrence caused by distant metastasis after initial treatment (after surgery and treatment before or after the surgery) for breast cancer aiming at curing. However, local recurrence for which radical excision is applicable (this represents the chest wall surrounded by the lower edge of the clavicle as the upper limit of surgical site, the costal arch as the lower limit, the midline of the sternum as interior limit, and the anterior edge of the latissimus dorsi muscle as the outer limit) is excluded.

(3) Endocrine therapy for metastatic breast cancer is planned.

(4) Performance status (PS) of ECOG is 0 or 1 [Note 4].
(See 3.5 Evaluation of Performance Status (PS))

Note 4: A case determined as PS 2 because of decreased activity due to bone metastasis will be considered as eligible.

(5) Previous endocrine therapy for breast cancer corresponds to either of the following. No distinction is made according to the type of endocrine therapeutic medicines used.

① A case that has continuously received endocrine therapeutic medicines as a postoperative adjunct therapy and in which recurrence occurred within 5 years after the initiation of the endocrine therapy.

② A case that received endocrine therapy as the primary treatment for metastatic breast cancer [Note 5] and in which progression of the disease occurred within 9 months after the initiation of the endocrine therapy.

Note 5: A case that received postoperative endocrine therapy for at least 5 years and had metastasis 5 or more years after the initiation of the endocrine therapy will not be included. In addition, a case that discontinued postoperative endocrine therapy within 5 years for any reason and then had metastasis and recurrence will also not be included.

(6) Previous chemotherapy for breast cancer corresponds to either of the following:

① No previous chemotherapy was administered.

② When chemotherapy was administered as a preoperative or postoperative adjunct therapy, it has been at least 6 months (168 days, 24 weeks) since the final day of administration.

(7) Previous radiotherapy for breast cancer corresponds to the following:

① It has been at least 14 days after the final day of radiotherapy administration.

(8) Agreement about the participation in the study will be obtained from the subject him/herself using the consent form (Appendix G).

6.3 Exclusion criteria

A case that corresponds to any of the following is excluded from participating in this study as a subject.

(1) HER2-positive breast cancer (See 3.7 Determination of HER2 expression status)

(2) A case not indicated for endocrine therapy

(3) Any other case that a physician determines to be unsuitable for participation in this study

7 Scientific rationale of the study

7.1 Choice of outcome measures

No single index to evaluate the efficacy and safety of cancer therapy has been established; therefore, a multiple outcome measures will be used in this study. Due to the difficulties involved in making multiple comparisons when performing statistical analyses, clinical benefit rate (CBR) was set as the primary outcome measures for the evaluation of the clinical benefit of secondary treatment with endocrine therapeutic medicines for breast cancer with low sensitivity to the primary endocrine therapy in this study. In addition, the objectives of treatment of metastatic breast cancer are the improvement of life prognosis, alleviation of symptoms, and maintenance and improvement of HRQoL; therefore, overall survival, adverse events, and HRQoL are chosen as secondary outcome measures. The tumor-related outcomes of progression free survival, time to treatment failure and response rate are set as secondary outcomes.

7.2 Scientific rationale and grounds for research hypothesis

The usual current first-line drugs for postmenopausal breast cancer are aromatase inhibitors as a postoperative endocrine therapeutic medicine or as a primary endocrine therapy for metastatic breast cancer. Hence, it is presumed that most of cases registered in this study will have been administered an aromatase inhibitor in their previous treatment. The following reports detail the results of clinical trials related to the use of secondary endocrine therapy after previous treatment with an aromatase inhibitor.

[Tamoxifen]

- In the TARGET trial, which compared tamoxifen with anastrozole as primary endocrine therapies for metastatic breast cancer, 137 cases received administration of tamoxifen as the secondary endocrine therapy of the anastrozole group, and a CBR of 48.7% was obtained [25)].
- A CBR of 50% was obtained for the use of tamoxifen after anastrozole in a subtrial of the TARGET trial implemented in Switzerland (SAKK 21/95 subtrial).

[Other aromatase inhibitors]

- Nine previous reports examined the therapeutic effect of steroidal exemestane as the secondary treatment after primary treatment with a nonsteroidal aromatase inhibitor (anastrozole or letrozole), and the CBR was 12–55% [27)].

[Fulvestrant]

- In the EFECT trial, which compared steroidal exemestane with a fulvestrant loading dose regimen as secondary treatments after primary treatment with a nonsteroidal aromatase inhibitor (anastrozole or letrozole), the therapeutic effect was equivalent in each group, and the CBRs of fulvestrant and exemestane were 32.2% and 31.5%, respectively [28)].
- In a dose comparison study of fulvestrant (250 mg/month vs. 500 mg/month) conducted in patients

with cancer recurrence under postoperative endocrine therapy, the CBRs of the 250 mg/month and 500 mg/month groups were high (39.6% and 45.6%, respectively) and the 500 mg/month group also showed better overall survival [29, 30)].

[mTOR inhibitors: combination use of everolimus and endocrine therapy]

- In the BOLERO-2 trial, which compared exemestane with an mTOR inhibitor (everolimus) + exemestane as secondary treatments after primary treatment with a nonsteroidal aromatase inhibitor (anastrozole or letrozole), superiority of the combination of exemestane + everolimus was indicated, and the CBRs of the exemestane group and the everolimus + exemestane group were 59% and 79.6%, respectively [15, 16)].
- In the TAMRAD trial, which compared tamoxifen with everolimus + tamoxifen, the CBRs of the tamoxifen group and the everolimus + tamoxifen group were 42.1% and 61.1%, respectively; indicating the superiority of the combined use of everolimus + tamoxifen [17)].

As described above, CBRs of about 50% were obtained for several trial therapies tested as secondary treatments used after primary treatment with aromatase inhibitors, although there have been variations between different studies and medicines. Especially, a strong trend towards higher CBR was observed for the combined use of fulvestrant 500 mg and an mTOR inhibitor in a recent clinical study[31)].

This study targets breast cancer with low sensitivity to the primary endocrine therapy, and previous trial results may not always be applicable; therefore, the therapeutic effect of secondary endocrine treatment in this clinical population needs to be evaluated. On the other hand, a clinically acceptable minimum therapeutic effect must be guaranteed.

As a hypothesis to validate one of the study objectives {4.1 Objectives (1)}, as shown in 4.3 Research hypothesis (1), we hypothesized that when the CBR of secondary endocrine therapy for breast cancer with low sensitivity to the primary endocrine therapy is 30% or higher, it can be used as a therapeutic medicine in this study. This study examines the efficacy of general secondary endocrine therapy for breast cancer with low sensitivity to the primary endocrine therapy, and the efficacy by drug type is also examined as below {4.3 Research hypothesis (2)}:

- Cohort SERMs

  Selective Estrogen Receptor Modulators (SERMs): tamoxifen and toremifene.

- Cohort AIs

  Aromatase Inhibitors: anastrozole, letrozole, and exemestane.

- Cohort SERD

  Selective estrogen receptor downregulator (SERD): fulvestrant.

- Cohort combination therapy with mTORi
  Combined use of an endocrine therapeutic medicine and everolimus, which is an mTOR inhibitor.

7.3 Sample size determination

This study is an observational study, in which therapy is selected based on the preferences of medical staff and patients. The study does not aim to compare different cohorts. To test the major study hypothesis, the threshold clinical benefit rate is set as 30%, and the expected clinical benefit rate is set as 50% in each cohort. Assuming the use of an accurate binomial test, it is estimated that the required number of cases is 43 under a condition of $\alpha=0.05$ (one-tailed) and $\beta=0.2$. Assuming a dropout rate of about 10%, the target samle size estimated to be required for each cohort was set at 50. This study is an observational study, and cases will not always be evenly distributed to each cohort. However, it is needed to set the sample size corresponding to the 4 groups at a maximum [(4) Selection of drugs]. Therefore, a target case quantity of at least 200 was set. Meanwhile, registration will continue until all 4 groups achieve the target number of 50 cases per group within the time frame set for registration, even after a total registration number of 200 cases is achieved.

8 Protection of Patients

All researchers and data providers involved in this study will conduct the study in compliance with the Declaration of Helsinki and the "Ethical Guidelines for Medical and Health Research Involving Human Subjects" (December 22, 2014) jointly developed by the Ministry of Education, Culture, Sports, Science and Technology and the Ministry of Health, Labour and Welfare of Japan.

9 Procedures to obtain informed consent etc.

Clinical investigators provide information documents approved by the ethical review committee of their host facility to each potential study subject before registration, together with an adequate explanation of what the study will involve for the subjects. After the explanation, sufficient time will be given so that the subjects can raise questions and make a judgment about whether or not to participate. It will be confirmed whether the subjects understood the contents of the study well, then participation in the study will be requested and voluntary agreement will be obtained as a completed and signed consent form.

When agreement to participate in the study is obtained from a subject, the subject will enter the date of the agreement and his/her name and provide their seal or signature on the consent form. In addition, the clinical investigators and research collaborators (when the research collaborators provided supplementary explanation) will enter the date of the explanation and his/her name and seal or signature. Two photocopies of the consent form are created for each subject. One copy of the consent form is provided for the patient and the other copy is filed at the facility. The original copy is filed with the subject’s medical records.

The following terms are explained to study subjects when informed consent is obtained:

(1) Title of the study and the procedures involved in implementation of the study
(2) Name of the research institute and principal investigator
(3) Objectives and significance of the study
(4) Methods and duration of the study
(5) Reasons why the subject was selected for the study
(6) Risks and benefits associated with participation in the study
(7) Withdrawal of consent after agreeing to participate in the study
(8) Treatment after withdrawal of consent when the subject does not wish to participate in the study
(9) Disclosure of information of the study
(10) Inspection and access to the research data
(11) Handling of personal information
(12) Methods of storage and disposal of collected information
(13) Research funding and conflicts of interest
(14) Consultation desk regarding the study
(15) Burden of expenses and rewards
(16) Other methods of treatment
(17) Treatment after the completion of the study

(18) Provision of new information related to the study

(19) Compensation for health hazards

(20) Utilization for research and transfer of the collected information

(21) Inspection of the collected information by bystanders

(22) Contact information of principal investigators and clinical investigators of the facility

10 Handling of personal information

(1) Privacy protection

For protection of privacy and personal information, persons involved in implementation of this study will exercise adequate care in ensuring the protection of privacy of study subjects and personal information. Data of the study subjects obtained in this study will not be used for any other purpose than meeting the objectives of this study. In addition, information that can specify the study subjects will not be used when the study results are published.

(2) Linkable anonymization

Identification and inquiry of registered patients is conducted using registry numbers issued at the time of registration (linkable anonymization). Information from which bystanders can directly identify a patient, such as the name, initials, and date of birth of patients, will not be registered into the database of the CSPOR data center.

(3) Timing and method of anonymization

Anonymization is conducted at the CSPOR data center at the registration of cases.

The CSPOR datacenter issues registry numbers for patient identification numbers described in the "Case registration card" and registered facilities. The data center will book the registry numbers issued on a "Confirmation note for case registration", which is sent to the clinical investigators.

Registry numbers will be used for the identification of subjects required for data collection (including QoL questionnaires) and subsequent inquiries. A correspondence table will be securely managed and stored by the CSPOR data center.

11 Burden and expected risks and benefits for study subjects

Treatment implemented in this study does not exceed the range of regular clinical practice, and the treatment will be conducted based on the preferences of medical staff and patients. Furthermore, the techniques used and the frequency of image assessment implemented to evaluate the therapeutic effect and side effects do not exceed the range of regular clinical practice. Therefore, it is considered that there are no increased risks or benefits for study subjects associated with participation in this study.

QoL evaluation using survey sheets will be conducted in this study. The investigation time for a single survey is ≤ 15 minutes, and the survey will be performed 3 times (at the time of enrollment and at 1 and 3 month after the initiation of the protocol treatment). Therefore, no excessive burden is borne by study subjects, and it is considered that the level of invasion associated with participation in this study is minor.

12 Methods of storage and disposal of specimens and information

12.1 Storage

Records of the consent given by subjects, test data etc. for the preparation of reports, certificates of approval by the ethical committee, and records prepared in medical institutes are securely stored under the supervision of the principal investigator. However, when the storage methods used for these materials (storage site and person in charge of storage) are specified to be at a collaborating research facility, storage will be conducted in accordance with the same rules as those used for central storage. Adequate care will be exercised for storage to avoid leakage of personal information.

The duration of information storage is up to 5 years from the discontinuation or completion of the study.

12.2 Disposal

When information are disposed of, adequate care will be exercised to avoid leakage of personal information by deleting electronic data and shredding printed documents containing personal information.

13 Contents and methods of report to Chief Executive of research implementing entity

13.1 Contents of report to Chief Executive of research implementing entity

Principal investigators in collaborating research institutes will report the following items to the Chief Executive of the research implementing entity.

Meanwhile, when the contents of the report to the Chief Executive of the research implementing entity are specified by the research institute, the report is conducted in accordance with the provision.

- Progress of the study
- Status of implementation of informed consent procedure
- Status of management of personal information
- The presence or absence of problems during the study
- Details of and measures taken to overcome any problems

13.2 Timing and methods of reporting

When the timing and methods of reporting to the Chief Executive of the research implementing entity are specified by the research institute, the report will be conducted in accordance with the provision. When provision is not provided by the research institute, principal investigators will prepare a "Status report on the study implementation" containing the contents of 13.1 and report to the chief executive of the research implementing entity.

The timing of reporting is as follows:

- End of every fiscal year
- End of the study

14 Research funding and conflicts of interest

14.1 Research funding

This study is conducted based on the support of research funds from the Investigator Initiated-Sponsored Research (IISR) (investigator sponsored study) of the Externally Sponsored Research (ESR) program of AstraZeneca K.K. to the General Incorporated Association of CSPOR-BC (http://www.astrazeneca.com/Research/externally-sponsored-research).

All responsibilities for the planning of the study, implementing institutes/facilities, and approval of the Ethical Review Board, implementation of the study, analysis, interpretation, and publication of the study results, and ensuring transparency of the study are assumed by researchers, and AstraZeneca K.K. is not involved in such decision-making.

The above will be clearly described at conference presentations and paper publications of the study results.

14.2 Disclosure of conflict of interest

(1) Disclosure of conflict of interest of members of executive committee of the study

The status of conflict of interest of the executive committee of this study is disclosed below. Meanwhile, disclosure of the status of conflict of interest was conducted in accordance with the "Breast Cancer Clinical Research Conflict of Interest Disclosure Policy (renewed on April 1, 2015)" and "Regulations regarding Breast Cancer Clinical Research Conflict of Interest Disclosure Policy (renewed on June 9, 2015)" presented by the Japanese Breast Cancer Society (http://www.jbcs.gr.jp/AboutJBCS/rieki.html).

Name of researchers and role in this study	Situation of conflict of interest* (as of July 31, 2015)
Naruto Taira (Research representative)	N.T. received research funding from AstraZeneca K.K. in 2014.
Tomomi Fujisawa (Research representative)	All items are not applicable.
Kazuhiro Araki (Executive committee member)	All items are not applicable.

Takayuki Iwamoto (Executive committee member)	All items are not applicable.
Kentaro Sakamaki (Person in charge of statistical analysis)	All items are not applicable.

* Disclosed terms: board member and adviser, stock, patent royalty, lecture's fee etc., and manuscript fee etc.

(2) Disclosure of conflict of interest in principal investigators of collaborating research institutes

When disclosure of conflicts of interest of principal investigators is required at ethical review of the study, the disclosure will be conducted by the principal investigators themselves in accordance with the policy specified by the Ethical Review Board of the collaborating research institute. The report should be submitted in accordance with the style specified by the research institute.

15 Method of disclosure for information about the study

15.1 Registration of the outline and results of the study

A research proposal for this study is registered with the UMIN Clinical Trials Registry (UMIN-CTR, http://www.umin.ac.jp/ctr/index-j.htm) before the entry of the first study subject to disclose the information. Furthermore, the information will be renewed on a timely basis in response to changes in the protocol and development of the study. When the study is completed, the study results will be registered without delay.

15.2 Publication of study results

When the study is completed, the study results will be published after taking measures required for the protection of the human rights of study subjects and involved persons or the rights and benefits of researchers etc. and their involved parties without delay. Publication of the study results will be determined by the steering committee based on a proposal by the executive committee of this study. Publication related to this study will be appropriately conducted based on a preliminary publication plan, which is specified separately. Planned presentation styles are conference presentations and submission of papers to medical journals.

16 Response to consultation etc. from study subjects and involved persons

The followings are described in a briefing paper as contacts for consultation etc. from study subjects and interested persons.

- Name, affiliation, and contact information of the research representative of this study.
- Name, affiliation, and contact information of principal investigators of research institutes.
- Contact information of consultation services designated by collaborating research institutes, if there are any available.

17 Informed consent by legally acceptable representative

This study has no legally acceptable representative.

18 Economic burden and rewards for study subjects

Medical examination of this study will be conducted within the normal range of regular clinical practice. Accordingly, all medical expenses including medication and examination during participation in the study will be paid as healthcare services provided by health insurance or by the patients participating in the study. No reward is provided to the study subjects who participate in this study.

19 Evaluation and reporting of adverse events

Adverse events are defined as all unfavorable symptoms and signs (including abnormal clinical laboratory test results) that develop in study subjects after the initiation of the study regardless of the presence or absence of causation by treatment.

When adverse events occur, clinical investigators must immediately take the required measures (examinations, treatment of the adverse events, and discontinuation of the treatment etc.) to ensure the safety of the study subjects.

19.1 Evaluation of adverse events

The naming of adverse events and their grades is determined based on the Common Terminology Criteria for Adverse Events v4.0 Japanese translation JCOG edition. Adverse events described as side effects in the package insert of each drug are considered to be known adverse events, and those not described are considered to be unknown adverse events.

(1) Evaluation duration of adverse events

The evaluation duration of adverse events in this study is from the registration to 30 days after the completion of the protocol treatment specified as the object of evaluation.

(2) Adverse events subjected to evaluation

Medical treatment and examination of subjects in this study is conducted within the normal range of regular clinical practice. Therefore, the risk of adverse events in subjects who participate in this study is similar to that in regular clinical practice. For this reason, information about adverse events of Grade 2 or milder is not collected in this study. Clinical investigators record the name, most severe grade, and date of initial development of the adverse events in a "Progress report" (Appendix X) for adverse events of Grade 3 and more severe. The naming of adverse events and their grades is determined based on the CTCAE v4.0.

(3) Handling of treatment-related death

In a case of treatment-related death, causal adverse events are defined as Grade 5. In addition, treatment-related death cases are urgently reported {19.2 (1)}. The final grades of causal adverse events are determined based on *ex post facto* examination including the urgent report.

19.2 Reporting of adverse events

When adverse events carrying an obligation to report corresponding to the below categories occur, a principal investigator reports to the secretariat. In addition, if serious adverse events ("serious" specified in ICH E2A) for which a causal correlation with the study treatment cannot be negated are observed, a report as stipulated by the reporting system of safety information on drugs etc. {Pharmaceutical and Medical Device Act, Section 68 (10), Reports of side effects} are appropriately implemented by the principal investigator's own responsibility in accordance with the procedures

stipulated by each medical institute.

(1) Adverse events carrying an urgent obligation to report

Adverse events corresponding to any of the following must be urgently reported via an "Emergency adverse event report" (Appendix X). These adverse events are defined as "Major adverse events".

① All deaths occurring during the study endocrine therapy.

② Deaths within 30 after the final administration of the study endocrine therapy and all deaths before the initiation of the next treatment.

"30 days" means 30 days counted from the day following a treatment day (the treatment day is defined as Day 0). All deaths occurred during this period are urgently reported regardless of the suspected causation.

However, even deaths occurring in a period other than the above (i.e., more than 30 days after the final administration of the study endocrine therapy and after the initiation of subsequent treatment) must be urgently reported when a correlation with the study endocrine therapy is suspected.

③ Unknown non-hematotoxicity observed before the initiation of the next treatment

(2) Adverse events carrying a regular obligation to report

Adverse events carrying a regular obligation to report are defined as any adverse events corresponding to Grade 3 or Grade 4 that are not adverse events carrying an urgent obligation to report {19.2 (1)}.

(3) Principal investigators' obligation to report and reporting procedures

① Urgent report

When adverse events occur that carry an urgent obligation to report {19.2 (1)}, a clinical investigator must immediately inform the relevant principal investigator. When the principal investigator is not available, the clinical investigator must cover the responsibility of the principal investigator.

When an adverse event subjected to urgent reporting is observed, the event must be orally reported to the secretariat within 24 hours and the principal investigator must complete an "Emergency adverse event report" (Appendix X) (It is also acceptable to use a form provided by the hospital) and submits a fax (03-5298-8536) or E-mail (info@csp.or.jp) to the CSPOR data center within 72 hours after knowing the incidence of the adverse event.

In addition, the principal investigator must prepare a case report (A4 size, free format) describing further information as a separate document and submit a fax to the CSPOR data center within 15 hours after knowing the incidence of the adverse event.

② Regular report

Principal investigators should record predefined terms in the "Progress report" (Appendix X) corresponding to the timing of the incidence of the adverse events and submit it to the CSPOR data center at the stipulated normal submission time of the Progress report.

19.3 Responsibility of the secretariat

(1) Determination of the necessity of discontinuance of registration and urgent notification to a facility

When the secretariat receives a report from a principal investigator, the secretariat must seek the decision of a research representative or his/her proxy regarding the urgency, importance, and degree of impact of the reported contents. The secretariat must then take measures as needed including discontinuation of registration (communication to CSPOR data center and all participating facilities) and urgent communication about terms that must be recognized to participating facilities. Furthermore, the principal investigator is strongly prompted to implement reporting as stipulated by the reporting system of safety information on drugs etc. based on the Pharmaceutical and Medical Device Act.

(2) Report to independent data monitoring committee

When the research representative determines that an adverse event reported as an urgent or regular report from a facility corresponds to an "adverse event carrying an obligation to report", the research representative must **report it to the independent data monitoring committee in writing within 15 days after knowing the incidence of the adverse event** and request review of the accuracy of the opinion of the research representative about the adverse event and any measures taken against the adverse event.

19.4 Examination by the independent data monitoring committee

The independent data monitoring committee will review the reported contents and make a recommendation in written form about future responses including handling of cases and the advisability of continuing registration to the research representative.

20 Compensation for health hazards

When adverse events occur in study subjects during their participation in this study, clinical investigators must immediately take required measures (examinations, treatment, withdrawal from the study, etc.) to ensure the safety of the study subjects. At the time, the best medical care within the range of the health care services provided by health insurance shall be provided. Monetary compensation for health hazards will not be provided in this study.

21 Response to queries regarding the delivery of medical care after the completion of the study

Medical care after the completion of the treatment specified in the protocol of this study is not stipulated. In addition, medical practice implemented in this study will be conducted within the normal range of regular clinical practice; therefore, no response related to the delivery of medical care after the completion of the study will be offered. Clinical investigators provide the best medical treatment aiming at prolonged survival, alleviation of symptoms, and maintenance and improvement of QoL, which are the goals for the treatment of metastatic breast cancer.

22 Handling of information related to health conditions of study subjects and study results

When information or research results that may influence the will of study subjects to continue participating in the study is obtained during the intervention period, the clinical investigator must immediately provide information documents describing the corresponding information and offer explanations of the following to the study subjects based on these documents:

- Corresponding information
- Continuation of the participation in the study is left to their discretion.

The clinical investigator offering the explanation will enter the date of the explanation and his/her name and seal or signature on the information documents and consent form, and the study subject will enter the date they received the information and his/her name and seal or signature. In addition, when a research collaborator offers a complementary explanation, the research collaborator will also enter the date of the explanation and his/her name and seal or signature. A copy of the materials will be provided to the study subjects.

The clinical investigator will confirm with the study subject whether he/she wishes to continue participating in the study, and will enter the date and the subject's answer on the original copy of the information documents and consent form and then file them. The clinical investigator will revise the information documents and consent form and obtain approval from the ethical review board of the facility, as needed. They must then explain the results to the study subject again using the revised information documents and consent form, and obtain voluntary consent for continuing participation in the study from the study subject in writing. The clinical investigator will enter the date when they gave the explanation and his/her name and

seal or signature on the consent form and the study subject will also enter the date of consent and his/her name and seal or signature. A copy of the consent form will be provided to the study subject and the original copy will be filed.

23 Outsourcing of research works, the contents of outsourced works, and method of supervision of outsourcing contractors

23.1 Outsourcing of data management

CSPOR Data Center

Locates in the Japan Clinical Research Support Unit (J-CRSU), NPO

J-CRSU Data Center

Representative (Director of Data Center)

Yasuo Ohashi (Department of Integrated Science and Engineering for Sustainable Society, Faculty of Science and Engineering, Chuo University)

Yushima D&A building 1F, 1-10-5 Yushima, Bunkyo-ku, Tokyo, 113-0034 Japan

TEL: 03-3254-8029

FAX: 03-5298-8536

E-mail: trial-bc@cspor-bc.or.jp

23.2 Contents of works

- Case registration
- Progress management
- Data management

23.3 Method of supervision

Members of the executive committee will supervise and give guidance about the appropriate implementation of the operations described in 25.1 by the CSPOR data center in addition to performing monitoring as described in a section 23.2.

24 Future utilization of samples and information

When the information obtained from subjects of this study are used for a study conducted for objectives different from this study or when the information are provided for other research institutes in the future, a new protocol will be developed and the study will be implemented under the approval of the ethical review board.

25 Monitoring and audit

25.1 Monitoring

Monitoring will be implemented to confirm whether the study is implemented safely and in accordance with the protocol of the study, and will evaluate whether data are accurately collected.

Monitoring will be conducted as in-house monitoring with case report forms collected by the CSPOR data center using computerized data processing results as reference in cooperation with the executive committee and CSPOR data center. Monitoring through facility visits is not planned.

25.2 Audit

Audit is not planned for this study because this study does not correspond to an "intervention study involving high degree of invasiveness" as described in the "Ethical Guidelines for Medical and Health Research Involving Human Subjects" (issued on December 22, 2014).

26 Approval by Ethical Review Boards

26.1 Approval at the start of participation in the study

When facilities start to participate in this study, each facility must submit necessary documents and receive approval from the ethical review board of the facility. When approval is obtained, the facility sends a copy of the certificate of approval to the secretariat. The facility will file the original copy of the certificate of approval, and the secretariat will file a copy of the certificate.

26.2 Annual renewal of the approval by the ethical review board

Review and annual renewal of approval for the study protocol and information documents for patients by the ethical review board of each facility will be conducted in accordance with the stipulations of each facility.

27 Compliance and changes to the protocol

27.1 Completion, withdrawal, and interruption of the study

(1) Completion of the study

The completion of the study is defined as the point when all follow-ups until the completion of the study duration are completed in all facilities. When the study is completed at each facility, principal investigators will submit a study completion report to the Chief Executive of the relevant research implementing entity and the research representative immediately.

(2) Withdrawal and interruption of the study

① The independent data monitoring committee examines the relevancy of continuation of the study, as needed. If at any point the committee determines that the continuation of the study is not appropriate, the committee will make a recommendation of withdrawal or interruption of the study to the research representative.

If the research representative decides to execute withdrawal of the study in accordance with the recommendation, the withdrawal, the reason of the withdrawal, and measures for participants will be communicated to principal investigators as soon as possible.

The principal investigators will report the situation in writing to the Chief Executives of research implementing entities and to the ethical review board of the facility at the same time. Suitable measures will then be taken for each study participant in accordance with the directions given by the research representative and the ethical review board of the facility.

② When a recommendation or direction of withdrawal is issued by the ethical review board, the research representative and principal investigators must take appropriate measures in accordance with the following:

A) When a recommendation or direction to withdraw the study is issued to the research representative by the ethical review board, the research representative must consider the withdrawal of the study. When withdrawal is decided, the withdrawal, the reasons for withdrawal, and measures for participating in the study will be communicated to principal investigators as soon as possible.
The principal investigators must report the situation in writing to the Chief Executives of research implementing entities and to the ethical review board of the facility at the same time. Suitable measures will then be taken for each participant in the study in accordance with the directions given by the research representative and the ethical review board of the facility.

B) When a recommendation or direction for withdrawal of the study is issued to the principal investigators by the ethical review board of the facility, the principal investigators must report it to the research representative immediately. The research representative who received the report will relay it to the independent data monitoring committee, which will examine the relevancy of the continuation of the study. When withdrawal or interruption of the study is decided based on the recommendation or direction from the ethical review board of the facility, the principal investigators will immediately report it together with the reasons to the Chief Executive of the relevant research implementing entity in writing.

③ Principal investigators must examine the advisability of continuation of the study when the following terms apply:

A) When it is determined that recruitment of the planned number of study subjects is difficult because of difficulty in recruiting study subjects.

B) When the objective of the study is achieved before the number of planned study subjects is recruited or before the end of the planned study duration.

C) When the independent data monitoring committee or ethical review board directed a change in the protocol and it is determined that it is difficult to accept this direction.

Note: "The independent data monitoring committee" refers to the independent data monitoring committee of the General Incorporated Association of CSPOR-BC organized by the General Incorporated Association of CSPOR-BC.

27.2 Compliance with the protocol

Researchers who conduct this study shall comply with the protocol of the study as long as the safety

and human rights of the participant are not blemished.

27.3 Deviation from the protocol

(1) Clinical investigators should not deviate from or change the protocol before obtaining approval of a chief executive of research implementing entity based on preliminary agreement of the research representative and preliminary review by the ethical review board of the facility.

(2) When it is determined that there is an imperative reason such as the avoidance of an emergency, clinical investigators can deviate from or change the protocol before obtaining preliminary agreement with the research representative and preliminary approval of the ethical review board of the facility. On such occasions, if the protocol is revised, the clinical investigators shall promptly submit a proposal to the ethical review board of the facility and obtain the approval of the research representative, the ethical review board of the facility, and the Chief Executive of the relevant research implementing entity.

(3) When deviations from the protocol occur, clinical investigators must record all deviations with the reasons. Principal investigators should report the deviations to the research representative and file a copy of the report.

27.4 Changes to the protocol

(1) Classification of changes to the protocol

Changes made to the protocol after the approval by the study review committee, steering committee, and ethical review board will be handled by division of the proposed changes into 2 categories, amendment and revision. Definition and handling of proposed changes in each category are as described below:

① Amendment

Amendments are partial changes to the protocol that have a possibility to increase the risk to the study participants or that are related to the primary outcomes of the study. Review and approval by the independent data monitoring committee is required. The date of approval by the independent data monitoring committee must be described on the cover page.

② Revision

Revisions are changes to the protocol that have no possibility to increase the risk to the study participants and/or that are not related to the primary outcomes of the study. No review by the independent data monitoring committee is required, but approval by the research representative and report to the independent data monitoring committee are required. Whether review and approval by the ethical review board of the facility are required is determined in accordance with agreement at each facility. The date of approval by the

research representative must be described on the cover page.

(2) Approval by ethical review board of the facility for amendment/revision of the protocol
When the protocol or information documents for patients is amended by obtaining approval of the independent data monitoring committee during the study, the amended protocol or information documents must be approved by the ethical review board of the facility. Whether the change to the contents is not amendment but revision and whether review and approval by the ethical review board of the facility is required is determined in accordance with agreement at each facility. When approval for the amendment is obtained from the ethical review board of the facility, principal investigators of each facility must send a copy of a certificate of the approval of the ethical review board to the secretariat. The principal investigators must file the original copy of the certificate of the approval, and the secretariat must file a copy of the certificate.

28 Attribution of intellectual property rights

Patent properties and other intellectual property rights acquired based on the research results of this study will be attributed to the General Incorporated Association of CSPOR-BC.

29 References

1） The Japanese Breast Cancer Society: General rules for clinical and pathological recording of breast cancer (17th ed.) [in Japanese]. Kanehara & Co., Ltd., Tokyo, 2012.

2） Breast cancer HER2 testing and pathology section. Guidelines for HER2 testing in breast cancer (4th ed.) [in Japanese]. http://pathology.or.jp/news/pdf/HER2-150213.pdf, accessed 25th March, 2014.

3） Eisenhauer EA, Therasse P, Bogaerts J, Schwartz LH, Sargent D, Ford R, Dancey J, Arbuck S, Gwyther S, Mooney M, Rubinstein L, Shankar L, Dodd L, Kaplan R, Lacombe D, Verweij J. New response evaluation criteria in solid tumours: revised RECIST guideline (version 1.1). Eur J Cancer. 2009; 45(2):228–47.

4） National Cancer Center, Center for Cancer Control and Information Services. http://gdbganjohojp/graph_db/index?lang=ja 2001, accessed 25th March, 2014.

5） Bonadonna G, Hortobagyi GN, Valagussa P. Textbook of breast cancer: a clinical guide to therapy, CRC Press, FL, USA, 2006.

6） Yamamoto N, Watanabe T, Katsumata N, Omuro Y, Ando M, Fukuda H, et al. Construction and validation of a practical prognostic index for patients with metastatic breast cancer. J Clin Oncol 1998; 16(7):2401–8.

7） Watanabe T. Evidence produced in Japan: tegafur-based preparations for postoperative chemotherapy in breast cancer. Breast cancer (Tokyo, Japan) 2013; 20(4):302–9.

8） Japanese Breast Cancer Society, The clinical practice guideline for breast cancer based on the evidence. 1. Pharmacotherapy [in Japanese]. (2015). Kanehara & Co., Ltd., Tokyo, 2015.

9） Cardoso F, Costa A, Norton L, Senkus E, Aapro M, André F, et al. ESO-ESMO 2nd international consensus guidelines for advanced breast cancer (ABC2). Ann Oncol 2014:mdu385.

10） Chlebowski RT. Strategies to overcome endocrine therapy resistance in hormone receptor-positive advanced breast cancer. Clin Investig 2014;4(1):19–33.

11） DeFriend DJ, Anderson E, Bell J, Wilks DP, West CM, Mansel RE, Howell A. Effects of 4-hydroxytamoxifen and a novel pure antioestrogen (ICI 182780) on the clonogenic growth of human breast cancer cells *in vitro*. Br J Cancer 1994;70(2):204–11.

12） Dauvois S, White R, Parker MG. The antiestrogen ICI 182780 disrupts estrogen receptor nucleocytoplasmic shuttling. J Cell Sci 1993;106(Pt. 4):1377–88.

13） DeFriend DJ, Howell A, Nicholson RI, Anderson E, Dowsett M, Mansel RE, Blamey RW, Bundred NJ, Robertson JF, Saunders C, et al. Investigation of a new pure antiestrogen (ICI 182780) in women with primary breast cancer. Cancer Res 1994;54(2):408–14.

14） Robertson JF, Llombart-Cussac A, Feltl D, et al. Fulvestrant 500 mg versus anastrozole as first-line treatment for advanced breast cancer: Overall survival from the phase II “first” study. 2014 San Antonio Breast Cancer Symposium; Abstract S6–04.

15） Baselga J, Campone M, Piccart M, Burris HA 3rd, Rugo HS, Sahmoud T, Noguchi S, Gnant M, Pritchard KI, Lebrun F, Beck JT, Ito Y, Yardley D, Deleu I, Perez A, Bachelot T, Vittori L, Xu Z, Mukhopadhyay P, Lebwohl D, Hortobagyi GN. Everolimus in postmenopausal hormone-receptor-positive advanced breast cancer. N Engl J Med 2012;366(6):520–9.

16） Piccart M, Hortobagyi GN, Campone M, Pritchard KI, Lebrun F, Ito Y, Noguchi S, Perez A, Rugo HS, Deleu

I, Burris HA 3rd, Provencher L, Neven P, Gnant M, Shtivelband M, Wu C, Fan J, Feng W, Taran T, Baselga J. Everolimus plus exemestane for hormone-receptor-positive, human epidermal growth factor receptor-2-negative advanced breast cancer: overall survival results from BOLERO-2. Ann Oncol 2014;25(12):2357–62.

17) Bachelot T, Bourgier C, Cropet C, Ray-Coquard I, Ferrero JM, Freyer G, Abadie-Lacourtoisie S, Eymard JC, Debled M, Spaëth D, Legouffe E, Allouache D, El Kouri C, Pujade-Lauraine E. Randomized phase II trial of everolimus in combination with tamoxifen in patients with hormone receptor-positive, human epidermal growth factor receptor 2-negative metastatic breast cancer with prior exposure to aromatase inhibitors: a GINECO study. J Clin Oncol 2012;30(22):2718–24.

18) Cella DF. Measuring quality of life in palliative care. Semin Oncol 1995; 22(2 Suppl 3): 73-81.

19) Shimozuma K, Ohashi Y, Takeuchi A, et al. Feasibility and validity of the Patient Neurotoxicity Questionnaire during taxane chemotherapy in a phase III randomized trial in patients with breast cancer: N-SAS BC 02. Support Care Cancer 2009;17(12):1483–1491.

20) Basch E, Abernethy AP, Mullins CD, et al. Recommendations for incorporating patient-reported outcomes into clinical comparative effectiveness research in adult oncology. J Clin Oncol 2012;30(34):4249–4255.

21) Stockler M, Wilcken NR, Ghersi D, et al. Systematic reviews of chemotherapy and endocrine therapy in metastatic breast cancer. Cancer Treat Rev 2000;26(3):151–168.

22) Fallowfield LJ, Leaity SK, Howell A, Benson S, Cella D: Assessment of quality of life in women undergoing hormonal therapy for breast cancer: validation of an endocrine symptom subscale for the FACT-B. Breast Cancer Res Treat 1999;55:189–99.

23) Cella DF, Tulsky DS, Gray G, Sarafian B, Linn E, Bonomi A, et al.: The Functional Assessment of Cancer Therapy scale: development and validation of the general measure. J Clin Oncol. 1993;11:570–9.

24) Brady MJ, Cella DF, Mo F, Bonomi AE, Tulsky DS, Lloyd SR, et al.: Reliability and validity of the Functional Assessment of Cancer Therapy-Breast quality-of-life instrument. J Clin Oncol 1997;15:974–86.

25) Thürlimann B, Robertson JF, Nabholtz JM, Buzdar A, Bonneterre J; Arimidex Study Group. Efficacy of tamoxifen following anastrozole ('Arimidex') compared with anastrozole following tamoxifen as first-line treatment for advanced breast cancer in postmenopausal women. Eur J Cancer. 2003;39(16):2310–7.

26) Thürlimann B, Hess D, Köberle D, Senn I, Ballabeni P, Pagani O, Perey L, Aebi S, Rochlitz C, Goldhirsch A. Anastrozole ('Arimidex') versus tamoxifen as first-line therapy in postmenopausal women with advanced breast cancer: results of the double-blind cross-over SAKK trial 21/95—a sub-study of the TARGET (Tamoxifen or 'Arimidex' Randomized Group Efficacy and Tolerability) trial. Breast Cancer Res Treat 2004; 85(3):247-54.

27) Beresford M, Tumur I, Chakrabarti J, Barden J, Rao N, Makris A. A qualitative systematic review of the evidence base for non-cross-resistance between steroidal and non-steroidal aromatase inhibitors in metastatic breast cancer. Clin Oncol (R Coll Radiol). 2011;23(3):209–15.

28) Chia S, Gradishar W, Mauriac L, Bines J, Amant F, Federico M, Fein L, Romieu G, Buzdar A, Robertson JF, Brufsky A, Possinger K, Rennie P, Sapunar F, Lowe E, Piccart M. Double-blind, randomized placebo controlled trial of fulvestrant compared with exemestane after prior nonsteroidal aromatase inhibitor therapy

in postmenopausal women with hormone receptor-positive, advanced breast cancer: results from EFECT. J Clin Oncol 2008; 26(10):1664–70.

29） Di Leo A, Jerusalem G, Petruzelka L, Torres R, Bondarenko IN, Khasanov R, Verhoeven D, Pedrini JL, Smirnova I, Lichinitser MR, Pendergrass K, Garnett S, Lindemann JP, Sapunar F, Martin M. Results of the CONFIRM phase III trial comparing fulvestrant 250 mg with fulvestrant 500 mg in postmenopausal women with estrogen receptor-positive advanced breast cancer. J Clin Oncol 2010; 28(30):4594–600.

30） Di Leo A, Jerusalem G, Petruzelka L, Torres R, Bondarenko IN, Khasanov R, Verhoeven D, Pedrini JL, Smirnova I, Lichinitser MR, Pendergrass K, Malorni L, Garnett S, Rukazenkov Y, Martin M. Final overall survival: fulvestrant 500 mg vs 250 mg in the randomized CONFIRM trial. J Natl Cancer Inst 2014;106(1):djt337.

31） Massarweh S1, Romond E, Black EP, Van Meter E, Shelton B, Kadamyan-Melkumian V, Stevens M, Elledge R. A phase II study of combined fulvestrant and everolimus in patients with metastatic estrogen receptor (ER)-positive breast cancer after aromatase inhibitor (AI) failure. Breast Cancer Res Treat 2014 Jan;143(2):325-32.

30 Appendices

A) "Ethical Guidelines for Medical and Health Research Involving Human Subjects" issued on December 22, 2014 by the Ministry of Education, Culture, Sports, Science and Technology and the Ministry of Health, Labour and Welfare of Japan

B) Case registration card

C) Confirmation note for case registration

D) Progress report

E) Emergency adverse event report

F) QoL questionnaire

G) Written informed consent
