# Supplementary material for: Health-related quality of life and estimation of the minimally important difference in the Functional Assessment of Cancer Therapy-Endocrine Symptom score in postmenopausal ER+/HER2- metastatic breast cancer with low sensitivity to endocrine therapy
Source: PLoS One. 2022 Nov 29;17(11):e0278344. doi: 10.1371/journal.pone.0278344 (PMC9707754; doi:10.1371/journal.pone.0278344)
Supplement: S2 Protocol — (PDF) [file pone.0278344.s004.pdf]

HORSE-BC

HOrmonal therapy ReSistant Er positive
metastatic Breast Cancer cohort study

一般社団法人 CSPOR-BC　臨床研究支援事業

内分泌療法耐性エストロゲン受容体陽性転移乳がんに対する二次内分泌療法のコホート研究実施計画書

Hormonal therapy resistant estrogen-receptor positive metastatic breast cancer cohort study

研究代表者/プロトコール作成:

平　成人　　岡山大学病院　乳腺・内分泌外科
〒700-8558 岡山県岡山市北区鹿田町 2-5-1
PHONE　086-235-7265
FAX　086-235-7269
E-mail　ntaira@md.okayama-u.ac.jp

藤澤 知巳　　群馬県立がんセンター　乳腺科
〒373-8550 群馬県太田市高林西町 617-1
PHONE　0276-38-077
FAX　0276-38-8386
E-mail　fujisawa@gunma-cc.jp

研究事務局:

一般社団法人 CSPOR-BC
〒101-0021 東京都千代田区外神田 2-19-3 お茶の水木村ビル 4F
PHONE　03-5294-7288
FAX　03-5294-7290
E-mail　trial-bc@cspor-bc.or.jp

データセンター（日本臨床研究支援ユニット内）
FAX：03-5298-8536　　電話：03-3254-8029
受付時間：平日 10 時～17 時(祝祭日，土曜・日曜，年末年始は受け付けない)

原案作成：	2015 年 3 月 25 日
CSPOR-BC 運営委員会承認：	2015 年 8 月 12 日
Ver1.0 作成：	2015 年 8 月 12 日
岡山大学研究倫理審査専門委員会承認：	2015 年 10 月 28 日

内 容

0　概要 5
0.1　研究デザイン 5
0.2　目的 5
0.3　研究仮説 6
0.4　研究対象者 6
0.5　研究の方法 6
0.6　評価項目 6
0.7　目標症例登録数と研究期間 6
1　研究の名称 7
2　研究の実施体制 8
2.1　実行委員会 8
2.2　実行委員会のミッション 8
2.3　(社)CSPOR-BC 運営委員会 8
2.4　運営委員会のミッション 9
2.5　CSPOR-BC 試験審査委員会 9
2.6　試験審査委員会のミッション 9
2.7　(社)CSPOR-BC 独立データモニタリング委員会 9
2.8　(社)CSPOR-BC データマネジメント委員会 10
2.9　CSPOR データセンター 10
2.10　(社) CSPOR-BC 事務局 10
2.11　共同研究機関 10
3　本研究で用いる用語の定義 11
3.1　「人を対象とする医学系研究に関する倫理指針」の遵守 11
3.2　一次内分泌療法低感受性乳がん 11
3.3　臨床病期(stage)分類 12
3.4　組織学的分類 14
3.5　Performance Status（PS）の評価 14
3.6　ホルモン受容体発現の判定 15
3.7　HER2 発現状況の判定 16
3.8　効果判定 17
3.9　本研究における評価項目の定義 23
4　研究の目的及び意義 25
4.1　研究の目的 25
4.2　研究の着想に至った背景 25

4.3　研究仮説 29
4.4　研究の意義 29
5　研究の方法及び期間 30
5.1　研究デザイン 30
5.2　症例の登録 31
5.3　治療 32
5.4　調査 36
5.5　健康関連 Quality of life の調査 44
5.6　データの提出 47
5.7　報告書への記載項目 48
5.8　データの取り扱い 49
5.9　予定症例登録数と研究期間 49
5.10　解析・分析の方法 49
6　研究対象者の選定方針 52
6.1　対象集団の定義 52
6.2　選択基準 52
6.3　除外基準 53
7　研究の科学的合理性の根拠 54
7.1　評価項目の設定根拠 54
7.2　研究仮説の科学的合理性と根拠 54
7.3　目標症例数の設定根拠 56
8　患者の保護 57
9　インフォームドコンセントを受ける手続き等 57
10　個人情報の取り扱い 58
11　研究対象者に生じる負担並びに予想されるリスク及び利益 59
12　試料・情報の保管及び廃棄の方法 59
12.1　保管 59
12.2　廃棄 59
13　研究機関の長への報告内容及び方法 59
13.1　研究機関の長への報告内容 59
13.2　報告の時期と方法 59
14　研究資金・利益相反 60
14.1　研究資金 60
14.2　利益相反状態の開示 60
15　研究に関する情報公開の方法 61
15.1　研究の概要及び結果の登録 61
15.2　研究結果の公表 61
16　研究対象者等及びその関係者からの相談等への対応 61

17　代諾者からのインフォームドコンセントについて 61
18　研究対象者の経済的負担・謝礼 61
19　有害事象の評価と報告 62
19.1　有害事象の評価 62
19.2　有害事象の報告 62
19.3　事務局の責務 63
19.4　独立データモニタリング委員会での検討 64
20　健康被害に対する補償 65
21　研究実施終了後における医療の提供に関する対応 65
22　研究対象者の健康状態に係わる情報，および研究結果の取り扱い 65
23　研究業務の委託，当該業務内容及び委託先の監督方法 66
23.1　データメネジメントの委託先 66
23.2　業務の内容 66
23.3　監督の方法 66
24　試料・情報の将来利用について 66
25　モニタリング及び監査 66
25.1　モニタリング 66
25.2　監査 66
26　倫理審査委員会での承認 67
26.1　研究への参加開始時の承認 67
26.2　倫理審査委員会承認の年次更新 67
27　研究計画の遵守，変更 67
27.1　研究の終了，中止，中断 67
27.2　研究実施計画書の遵守 68
27.3　研究実施計画書からの逸脱 68
27.4　研究実施計画書の変更 69
28　知的財産権の帰属 69
29　参考文献 70
30　添付書類（Appendix） 73

0　概要

0.1　研究デザイン

多施設共同，前向き観察研究

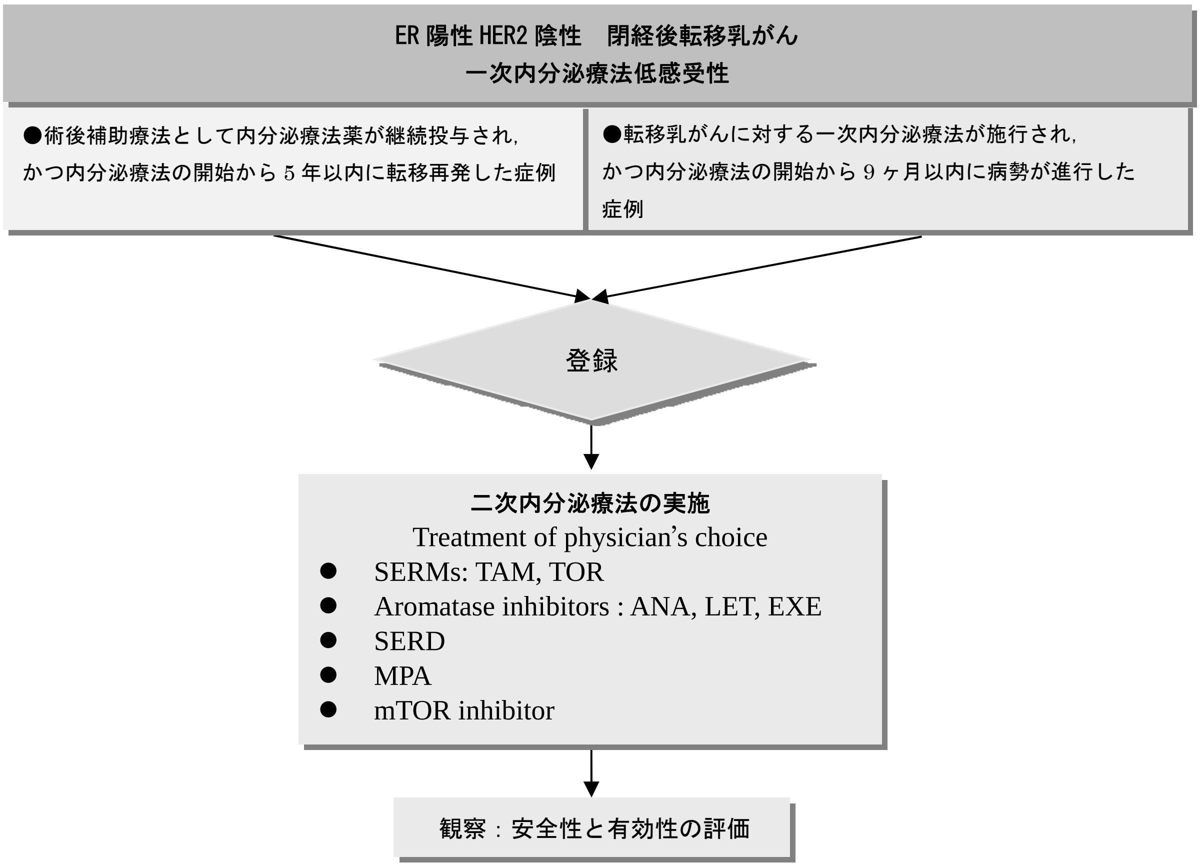

0.2　目的

(1)　一次内分泌療法に良好な臨床効果の得られなかったエストロゲン受容体陽性 HER2 陰性の閉経後転移乳がん（一次内分泌療法低感受性乳がん）に対する，二次内分泌療法全般，および薬剤種別の有効性と安全性を明らかにする。

(2)　前内分泌療法への反応性（術後内分泌療法開始から再発までの期間，あるいは転移乳がんに対する一次内分泌療法の無増悪期間），および腫瘍の生物学的特性（ER の発現強度，PgR の発現の有無）が，二次内分泌療法の効果に及ぼす影響を明らかにし，Hortobagyi の治療アルゴリズムを補完する情報を得る。

0.3 研究仮説

(1) 一次内分泌療法低感受性乳がんに対しても，近年の内分泌療法薬により 30%以上の臨床的有用率が期待できる。

(2) 一次内分泌療法低感受性乳がんに対する二次内分泌療法の治療効果には，前治療への反応性や腫瘍の生物学的特性が影響し，二次内分泌療法の効果を予測できる。

0.4 研究対象者

一次内分泌療法低感受性のエストロゲン受容体陽性 HER2 陰性，閉経後転移乳がんを対象とする。（詳細は6研究対象者の選定方針を参照）

0.5 研究の方法

医療者と患者の選好により，現代の内分泌療法薬を用いた治療を実施し，前向きに観察することによりその有効性と安全性を評価する。

0.6 評価項目

主要評価項目：

臨床的有用率

副次的評価項目：

無増悪生存期間，全生存期間，治療成功期間，化学療法までの期間，
奏効率，健康関連 quality of life，有害事象

0.7 目標症例登録数と研究期間

目標症例登録数：200 例以上

研究期間：2015 年 11 月～2019 年 10 月までの 4 年間
内，登録期間：2015 年 11 月～2016 年 10 月までの 1 年間
内，観察期間：2016 年 11 月～最後の症例登録から 3 年間

1　研究の名称

（1）　日本語名称

内分泌療法耐性エストロゲン受容体陽性転移乳がんに対する二次内分泌療法のコホート研究

（2）　日本語略記

内分泌療法耐性転移乳がんのコホート研究

（3）　英語名称

Hormonal therapy resistant estorgen receptor positive metastatic breast cancer cohort study

（4）　英語略記

HORSE-BC

2　研究の実施体制

本研究は，一般社団法人 CSPOR-BC（Comprehensive Support Project for Oncological Research of Breast Cancer：以下，(社)CSPOR-BC）による研究支援事業として行われる。
本研究を実施するために実行委員会を設置する。また研究組織は(社)CSPOR-BC が組織する
運営委員会，試験審査委員会，独立データモニタリング委員会，データマネジメント委員会が，
研究計画書の審査，方針の検討および決定，運営の管理，監督を行う。

2.1　実行委員会

委　員　長（観察研究代表者）

平　成人　　　　（岡山大学病院　乳腺・内分泌外科）Health ouccome research（HOR）担当
藤澤知巳　　　　（群馬県立がんセンター　乳腺科）

実行委員（50 音順）

荒木　和浩　　　（癌研有明病院　乳腺センター　乳腺内科）
岩本　高行　　　（岡山大学病院　乳腺・内分泌外科）

観察研究統計家（生物統計解析責任者）

坂巻顕太郎　　　（横浜市立大学学術院医学群　臨床統計学・疫学）

2.2　実行委員会のミッション

- データセンターおよび事務局と協力して行う，研究実施に関わる調整作業
- 研究実施状況の運営委員会への報告
- 研究全体の品質管理・品質保証に必要な事項
- 研究に参加する施設の質評価
- データセンターに対するデータ管理の支援
- 試験統計家に対する統計解析の支援
- 報告書の作成
- その他，各研究の円滑かつ効率的な実施に必要な事項

2.3　(社)CSPOR-BC 運営委員会

運営委員長

向井　博文（国立がん研究センター東病院　乳腺・腫瘍内科）

運営委員（50 音順）

相原　智彦　　　（啓明会　相原病院　理事長）
岩田　広治　　　（愛知県がんセンター中央病院　乳腺科）
大住　省三　　　（国立病院機構四国がんセンター　乳腺外科）
高橋　將人　　　（国立病院機構北海道がんセンター　乳腺科）
穂積　康夫　　　（自治医科大学附属病院　乳腺科）
山本　精一郎　　（国立がん研究センター　がん対策情報センター）

2.4 運営委員会のミッション

- 事業の長期計画，年次計画の策定と活動状況の財団への報告
- 事業遂行のための予算案の策定と，決算の財団への報告
- 事業を遂行するための各種小委員会の設立・改廃と小委員会委員の任命
- 小委員会の活動状況の監督
- その他，本事業の目的遂行に必要な事項

2.5 CSPOR-BC 試験審査委員会

委員長

遠山 竜也 （名古屋市立大学病院 乳腺内分泌外科）

副委員長

朴 英進 （東北薬科大学病院 乳腺外科）

委員（試験統計家他，50 音順）

手良向 聡 （試験統計家：京都府立医科大学大学院医学研究科 生物統計学）

高野 利実 （虎の門病院 臨床腫瘍科）

田村 研治 （国立がん研究センター中央病院 乳腺・腫瘍内科）

松原 伸晃 （国立がん研究センター東病院 乳腺・腫瘍内科）

2.6 試験審査委員会のミッション

- 新規に開始する臨床試験および付随研究の決定，および研究を公募する場合の公募要綱の決定と研究の採択
- 臨床試験および付随研究毎の実行委員会委員の任命
- 臨床試験および付随研究の実施状況の監督
- 臨床試験および付随研究に関する口頭発表・論文発表の承認
- その他事業で行う臨床試験および付随研究の円滑な実施と研究間の調整に必要な事項

2.7 (社)CSPOR-BC 独立データモニタリング委員会

独立データモニタリング委員長

中村 清吾 （昭和大学病院 乳腺外科）

独立データモニタリング副委員長

勝俣 範之 （日本医科大学武蔵小杉病院 腫瘍内科）

独立データモニタリング委員（50 音順）

柴田 大朗 （国立がん研究センター 多施設臨床試験支援センター）

柳澤 昭浩 （NPO 法人キャンサーネットジャパン）

2.8 (社)CSPOR-BC データマネジメント委員会

データマネジメント委員長

大住 省三　　　（国立病院機構四国がんセンター　乳腺外科）

データマネジメント委員（50 音順）

澤木 正孝　　　（愛知県がんセンター中央病院　乳腺科）

平 成人　　　（岡山大学病院乳腺・内分泌外科）

2.9 CSPOR データセンター

症例登録業務，進捗管理業務，データマネジメント業務を行う。

NPO 日本臨床研究支援ユニット(J-CRSU)内

J-CRSU データセンター

代表（データセンター長）

大橋靖雄　　　（中央大学理工学部人間総合理工学科 ）

〒113-0034　東京都文京区湯島 1-10-5　湯島 D&A ビル 1F

TEL：03-3254-8029

FAX：03-5298-8536

E-mail：trial-bc@cspor-bc.or.jp

2.10 (社) CSPOR-BC 事務局

(社) CSPOR-BC 事務局長

相原 智彦　　　（啓明会　相原病院　理事長）

(社) CSPOR-BC 事務局部長

石河 吉輝

〒101-0021　東京都千代田区外神田 2-19-3　お茶の水木村ビル 4F

一般社団法人 CSPOR-BC

TEL：03-5294-7288

FAX：03-5294-7290

E-mail：trial-bc@cspor-bc.or.jp

2.11 共同研究機関

最新の共同研究機関，および研究機関の責任医師氏名一覧は

http://cspor-bc.or.jp/index.html を参照

3 本研究で用いる用語の定義

3.1 「人を対象とする医学系研究に関する倫理指針」の遵守

本研究で用いる以下の用語は，文部科学省及び厚生労働省より平成 26 年 12 月 22 に公布された「人を対象とする医学系研究に関する倫理指針」(Appendix A)の第 1 章の第 2 に記載された，「用語の定義」に従う。

侵襲；介入；研究に用いられる情報；試料・情報；既存試料・情報；研究対象者；研究機関
共同研究機関；試料・情報の収集・分譲を行う機関；研究者等；研究責任者；研究機関の長
倫理審査委員会；インフォームド・コンセント；代諾者；代諾者等；個人情報；個人情報等；匿名化
連結可能匿名化；連結不可能匿名化；有害事象；重篤な有害事象
予測できない重篤な有害事象；モニタリング；監査

3.2 一次内分泌療法低感受性乳がん

本研究では，術後補助内分泌療法を継続中の患者が，内分泌療法の開始から 5 年以内に再発した症例，あるいは転移乳がんの一次内分泌療法に対して，内分泌療法の開始から 9 ヶ月以内に病勢進行した症例を一次内分泌療法低感受性乳がんと呼ぶ。

3.3 臨床病期(stage)分類

「乳癌取り扱い規約（第 17 版，2012 年）」を用いる[1)]。UICC-TNM 分類（第 7 版，2009 年）準拠

(1) T：原発巣[注1]

		大きさ（cm）	胸壁固定[注2]	皮膚の浮腫，潰瘍<br>衛星皮膚結節
TX		評価不可能		
Tis		非浸潤がんあるいは Paget 病		
T0		原発巣を認めず[注3, 4]		
T1[注5]		≦ 2.0	－	－
T2		2.0 ＜<br>≦ 5.0	－	－
T3		5.0 ＜	－	－
T4	a	大きさを問わず	＋	－
	b		－	＋
	c		＋	＋
	d	炎症性乳がん[注6]		

注 1：T の大きさは原発巣の最大浸潤径を想定しており，視触診，画像診断を用いて総合的に判定する。乳管内成分を多く含む癌で，触診径と画像による浸潤径との間に乖離がみられる場合は画像による浸潤径を優先する。乳腺内に多発する腫瘍の場合は最も大きい T を用いて評価する。

注 2：胸壁とは，肋骨，肋間筋および前鋸筋を指し，胸筋は含まない。

注 3：視触診，画像診断にて原発巣を確認できない。

注 4：異常乳頭分泌例，マンモグラフィの石灰化例などは T0 とはせず判定を保留し，最終病理診断によって Tis，T1mic などに確定分類する。

注 5：a（≦0.5），b（0.5＜ ≦1.0），c（1.0＜ ≦2.0）に亜分類する。
ただし，組織学的浸潤径が 0.1 cm 以下のものは T1mic として付記する。

注 6：炎症性乳がんは通常腫瘤を認めず，皮膚のびまん性発赤，浮腫，硬結を示すものを指す。腫瘤の増大，進展に伴う局所的な発赤や浮腫を示す場合はこれに含めない。

(2) N:所属リンパ節注1

		同側腋窩リンパ節 レベルⅠ,Ⅱ		胸骨傍リンパ節	同側腋窩リンパ節レベルⅢ注2	同側鎖骨上リンパ節
		可動	周囲組織への固定あるいはリンパ節癒合			
NX		評価不可能				
N0		−	−	−	−	−
N1		＋	−	−	−	−
N2	a	−	＋	−	−	−
	b	−	−	＋	−	−
N3	a	＋/−	＋/−	＋/−	＋	−
	b	＋ または ＋		＋	−	−
	c	＋/−	＋/−	＋/−	＋/−	＋

注 1:リンパ節転移の診断は触診と画像診断などによる。

注 2:UICC の TNM 分類第 7 版でいう鎖骨下リンパ節に相当する

(3) M:遠隔転移

M0 遠隔転移なし

M1 遠隔転移あり

(4) TNM 分類

		T0	T1	T2	T3	T4
M0	N0		I	IIA	IIB	IIIB
	N1	IIA	IIA	IIB	IIIA	IIIB
	N2	IIIA	IIIA	IIIA	IIIA	IIIB
	N3	IIIC	IIIC	IIIC	IIIC	IIIC
M1		IV	IV	IV	IV	IV

3.4 組織学的分類

「乳癌取り扱い規約(第 17 版, 2012 年)」を用いる [1)]。

1 非浸潤癌

1a.	非浸潤性乳管癌
1b.	非浸潤性小葉癌

2 浸潤癌

2a.	浸潤性乳管癌
2a1	乳頭腺管癌
2a2	充実腺管癌
2a3	硬癌
2b.	特殊型
2b1	粘液癌
2b2	髄様癌
2b3	浸潤性小葉癌
2b4	腺様嚢胞癌
2b5	扁平上皮癌
2b6	紡錘細胞癌
2b7	アポクリン癌
2b8	骨軟骨化生を伴う癌
2b9	管状癌
2b10	分泌癌(若年性癌)
2b11	浸潤性微小乳頭癌
2b12	基質産生癌
2b13	その他

3 Paget 病

3.5 Performance Status(PS)の評価

以下の ECOG scale 日本語訳を用いる

Grade	Performance Status
0	無症状で社会活動ができ，制限を受けることなく発病前と同様にふるまえる。
1	軽度の症状があり，肉体労働は制限を受けるが，歩行，軽労働や座業はできる。<br>例えば軽い家事，事務など。
2	歩行や身の回りのことはできるが，時に少し介助がいることもある。軽労働は<br>できないが，日中の 50%は起居している。
3	身の回りのある程度のことはできるが，しばしば介助がいり，<br>日中の 50%以上は就床している。
4	身の回りのこともできず，常に介助がいり，終日就床を必要としている。

3.6 ホルモン受容体発現の判定

(1) エストロゲン受容体(Estrogen Receptor：ER)発現

エストロゲン受容体α(ERα)の発現を免疫組織化学(IHC)法で評価する。
本研究では施設単位での評価を行い，以下の基準で ER 陽性と判定する。
J-score 分類：染色陽性細胞占有率 <u>1%以上を陽性</u>と判定する。
(Allred スコア分類では Proportion score により換算する)

(2) プロゲステロン受容体(Progesterone Receptor: PgR)の発現

プロゲステロン受容体(PgR)の発現を免疫組織化学(IHC)法で評価する。
本研究では施設単位での評価を行い，以下の基準で ER 陽性と判定する。
J-score 分類：染色陽性細胞占有率 <u>1%以上を陽性</u>と判定する。
(Allred スコア分類では Proportion score により換算する)

3.7 HER2 発現状況の判定

HER2 発現状況について，Immunohistochemistry（IHC）法および In situ hybridization （ISH）法による判定基準は，乳がん HER2 検査病理部会作成の HER2 検査ガイド乳癌編第四版に準じる[2)]。

（1） IHC 法の判定基準

下記判定でスコア 0， 1+を HER2 陰性，3+を HER2 陽性と定義する。

equivocal（スコア 2+）と判定された場合は，リフレックステスト（ISH 法を用いて同じ検体で），または新たな検査（IHC または ISH 法を用いて可能なら新たな検体で）を実施しなければならない。

判定	スコア	染色パターン
陽性	3+	強い完全な細胞膜の陽性染色がある癌細胞>10%
equivocal	2+	①弱～中等度の完全な細胞膜の陽性染色がある癌細胞>10%<br>②強い完全な細胞膜の陽性染色がある癌細胞≦10%
陰性	1＋	ほとんど識別できないどかすかな細胞膜の染色がある癌細胞>10%
	0	細胞膜に陽性染色なし，あるいは細胞膜の陽性染色がある癌細胞≦10%

（2） ISH 法の判定基準

下記判定にて陽性を HER2 陽性と定義する。

判定	基準
陽性	Single probe:<br>平均 HER2 コピー数≧6.0<br>Dual probe:<br>HER2/CEP17 比≧2.0; 平均 HER2 コピー数≧4.0 HER2/CEP17 比≧2.0; 平均 HER2 コピー数<4.0 HER2/CEP17 比<2.0; 平均 HER2 コピー数≧6.0
equivocal	Single probe:<br>平均 HER2 コピー数≧4.0， <6.0<br>Dual probe:<br>ER2/CEP17 比<2.0; 平均 HER2 コピー数≧4.0，<6.0
陰性	Single probe:<br>平均 HER2 コピー数<4.0<br>Dual probe:<br>ER2/CEP17 比<2.0; 平均 HER2 コピー数<4.0

3.8 効果判定

RECIST 基準(ver. 1.1)による測定可能病変を有する症例について，腫瘍縮小効果を評価し奏効率を算出する。最良総合効果が CR，PR のいずれかである被験者の割合を奏効率とする[3)]。

(1) 測定可能病変の定義

以下のいずれかに該当する病変を「測定**可能**病変」とする。

1） 以下のいずれかを満たす，リンパ節病変以外の病変(非リンパ節病変)

① 5mm 以下のスライス厚の CT にて最大径 10mm 以上

② 5mm を超えるスライス厚の CT にて最大径がスライス厚の 2 倍以上

③ ①または②を満たす軟部組織病変を有する，溶骨性骨転移病変

④ 他に測定可能な非嚢胞性病変を有さない場合の，①または②を満たす嚢胞性転移病変

2） 5mm 以下のスライス厚の CT にて短径が 15mm 以上のリンパ節病変
(短径が 10mm 以上，15mm 未満のリンパ節病変は測定不能病変であり，短径が 10mm 未満のリンパ節は病変ではない)

3） 胸部単純 X 線写真にて最大径 20mm 以上で，かつ周囲が肺野で囲まれている。
(縦隔や胸壁に接していない)

4） メジャーとともにカラー写真撮影ができる最大径 10mm 以上の表在性病変
(皮膚転移など)

上記以外のすべての病変を「測定**不能**病変」とする。
以下の病変等は検査法や病変の大きさによらず，測定不能病変とする。

- 骨病変(測定可能な軟部組織成分を有する溶骨性病変を除く)
- 嚢胞性病変{上記，1)の④を除く}
- 放射線治療等の局所治療の既往のある病変
- 髄膜病変
- 腹水，胸水，心嚢水
- 炎症性乳がん
- 皮膚/肺リンパ管症
- 画像による確認はできないが，触知可能な腹部腫瘤や腹部臓器腫大

(2)　標的病変の選択とベースライン評価

本試験の登録時（ベースライン）に認められた測定可能病変のうち，径（非リンパ節病変は長径，リンパ節病変は短径）の大きい順に 5 つまで，1 臓器あたり最大 2 個まで選択して標的病変とする。選択の際には，測定可能病変を有する臓器ができるだけ満遍なく含まれることと，繰り返し計測の際の再現性すなわち測りやすさを考慮して選択する。

選択した標的病変について頭側から尾側の順に部位，検査法，検査日，非リンパ節標的病変の長径，リンパ節標的病変の短径，および全ての標的病変の径の和を記録する。

(3)　非標的病変の選択とベースライン評価

標的病変として選択されなかった病変は，測定可能か否かを問わずすべて「非標的病変」として部位，検査方法，検査日を記録する。同一臓器や同一部位に同様の病変を多数認める場合，1 つの非標的病変として記録してよい。

（例：多発性骨盤リンパ節腫大，多発性肝転移）

(4)　腫瘍縮小効果の判定

本研究は観察研究であるため治療効果判定は基本的に主治医判断にて行うことを旨とする。ただし，当研究の対象が内分泌療法低感受性乳がんであるため病勢増悪が治療開始早期からみられる可能性もある。研究対象の安全性を確保し，治療方針決定のタイミングを見逃すことのないよう，治療開始3ヶ月目，6ヶ月目を目途に効果判定を行うことを規定する。

標的病変および非標的病変の評価は，登録前の検査法（造影，スライス幅等の撮影条件も同様とする）を用いて行い，標的病変の長径の測定（リンパ節は短径），非標的病変の消失または増悪の有無，新病変の有無を記録する。

＜標的病変の効果判定＞

CR<br>(Complete response)	すべての非リンパ節病変が消失し，すべてのリンパ節標的病変の短径が 10mm 未満となった場合。
PR<br>(Partial response)	標的病変の径和が，ベースラインの径和に比し 30%以上小さくなった場合。
SD<br>(Stable disease)	PR に該当する腫瘍縮小や PD に該当する腫瘍増大を認めない場合。
PD<br>(Progressive disease)	標的病変の径和が，それまでもっとも小さい径和に比して 20%以上大きくなり，かつ絶対値で 5mm 以上増加した場合（新病変の出現を含む）。
NE<br>(Not evaluable)	なんらかの理由で検査が行えない場合，または CR，PR，PD，SD いずれとも判定できない場合

最長径和の縮小率＝（治療前の径和－評価時の径和）／（治療前の径和）×100 ％

最長径和の増大率＝（評価時の径和－最小の径和）／（最小の径和）×100 ％

＊ 標的病変の径は測定可能な限り（例えば 5mm 未満であっても）実測値を記録するが，標的病変の径が「小さすぎて測定できない」と判断された場合には，CT のスライス厚によらず，腫瘍病変が残存していないと判断される時は径を 0mm とし，腫瘍病変が残存していると判断される時は径を 5mm とする。

＊ 縮小割合が PR の条件を満たし，同時に増大割合が PD の条件を満たす場合には PD とする。

＊ 治療中に 1 つの病変が分離した場合は，それぞれの径和に加算する。

＊ 治療中に複数の病変が癒合して境界が識別できなくなった場合は，癒合した病変の径を径和に加算する。病変どうしが接していても，病変の境界が識別可能な場合は各病変の径を径和に加算する。

＜非標的病変の効果判定＞

CR<br>(Complete response)	すべての非リンパ節非標的病変が消失し，腫瘍マーカーがすべて施設基準値上限以下となり，すべてのリンパ節非標的病変の短径が 10mm 未満となった場合。
Non-CR／non-PD	1 つ以上の非リンパ節非標的病変が消失しない，または 1 つ以上のリンパ節非標的病変の短径が 10mm 以上，または腫瘍マーカーのいずれかが施設基準値上限を超える場合。
PD<br>(Progressive disease)	非標的病変の「明らかな増悪」（新病変の出現を含む）<br>・明らかな増悪は表外のコメントを参照する。
NE<br>(Not evaluable)	なんらかの理由で検査が行えなかった場合，または CR，Non-CR/non-PD，PD いずれとも判定できない場合。

＜明らかな増悪＞

・測定可能病変を有する症例の場合，標的病変が PR や SD であっても，非標的病変の顕著な悪化により，治療を続けるよりもやめることのメリットが勝ると判断される場合，＜明らかな増悪＞とする。

・測定不能病変だけを有する症例では，例えば体積を目安とした場合，測定不能病変の体積の 73%の増大（測定可能病変の径の 20%増加に相当）に相当する腫瘍量を明らかに超えると判断されるような測定不能病変の増大を＜明らかな増悪＞とする。

＜新病変の出現＞

ベースラインでは存在しなかった病変が治療開始後に認められた場合，「新病変」とみなす。ただし「新病変」とするには，ベースライン評価時の検査とのスキャン方法や撮影モダリティの違いによる画像上の変化ではないことや，腫瘍以外の病態による画像上の変化ではないことを確認する必要がある。例えば，肝転移巣の壊死による病巣内に生じた嚢胞性病変は新病変とはしない。ベースラインにて必須としていなかった部位の検査により新たに認められた病変は新病変とする。

ベースラインで存在した病変がいったん消失した後に再出現した場合，総合効果が CR となった後の再出現であれば「PD」であるが，他の病変が残存している状態であれば，再出現のみで「新病変」や「PD」とするのではなく，標的病変であった場合には腫瘍径を径和に加算する。非標的病変であった場合は「PD」の定義における「明らかな増悪」に該当しない限り「Non-CR/non-PD」とする。

新病変である可能性があるが確定できない場合は新病変とはせず，臨床的に適切な時期を空けて画像検査を行う。再検した画像検査にて新病変であると確定した場合，新病変を疑った時点の画像検査日をもって新病変出現とする。

(5) 時点総合効果(Time point response)

時点総合効果は標的病変の効果，非標的病変の効果，新病変の出現の有無の組み合わせから，以下の表に従って，画像評価ポイント毎に判定する。

ベースラインで非標的病変が存在しない場合の総合効果は，標的病変の効果と新病変出現の有無により判定し，ベースラインで標的病変が存在しない場合の総合効果は非標的病変の効果と新病変の出現の有無により，下表に従って判定する。

＜時点総合効果の判定基準（標的病変を有する場合）＞

標的病変の効果	非標的病変の効果	新病変出現の有無	総合効果
CR	CR	No	CR
CR	Non-CR/Non-PD	No	PR
CR	Not evaluated	No	PR
PR	Non-PD or not all evaluated	No	PR
SD	Non-PD or not all evaluated	No	SD
Not all evaluated	Non-PD	No	NE
PD	Any	Yes or No	PD
Any	PD	Yes or No	PD
Any	Any	Yes	PD

＜時点総合効果の判定基準（標的病変を有さない場合）＞

非標的病変の効果	新病変出現の有無	総合効果
CR	No	CR
Non-CR/Non-PD	No	Non-CR/non-PD
Not all evaluated	No	NE
Unequivocal PD	Yes or No	PD
Any	Yes	PD

(6) 最良総合効果(Best Overall Response)

最良総合効果は，CR＞PR＞SD＞PD＞NE の順に良好であるとし，治療開始から後治療開始までを通じて最も良好な時点総合評価をもって最良総合効果とする。

なお本研究では 4 週間の持続期間による CR，PR の確定は不要である。

複数の区分の定義に該当する場合は，CR＞PR＞SD＞PD＞NE の順に，より良好なものに区分する。

SD 判定においては，治療開始日から起算して 6 週以降に SD の基準を満たしていることとする。1 回目の効果判定以前に明らかな原病の悪化（増悪）が認められ，画像による判定が一度も行われなかった場合は PD とし，有害事象や被験者拒否による治療中止のために判定が行われなかった場合は NE とする。

(7) 臨床的増悪

{3.8 (4) 腫瘍縮小効果の判定} で定義された方法以外で増悪と判断する場合，または測定不能病変の増悪判定は以下の基準に従って判定する。ただし，下記に該当する場合でも直ちに増悪と判断するものではなく，担当医師の判断を優先する。画像評価が可能な場合には画像評価を優先する。

① エコーによる増悪確認：エコーによる新病変の出現，胸水の増加等，明らかな増悪が認められた場合

② 骨病変に対する増悪確認：骨病変に対する骨シンチや PET の集積の増加，新病変の出現が認められた場合

③ 自覚症状の悪化：骨転移における明らかな骨痛の悪化，肺転移における明らかな呼吸困難感の悪化等，担当医師が増悪と判断した場合

3.9 本研究における評価項目の定義

(1) 臨床的有用率(clinical benefit rate: CBR)

全治療例のうち，治療の開始日から 6 カ月間「増悪」と判定されなかった患者の割合を臨床的有用率とする。

『6 カ月間「増悪」と判定されない患者』とは，治療開始から 3， 6 ヶ月目に実施する画像診断による時点総合効果{3.8 (5) 時点総合効果の判定基準}が，いずれの調査時点でも CR，PR または SD のいずれか。かつ，治療開始から 6 ヶ月間に臨床的増悪{3.8(7) 臨床的増悪}を認めない患者とする。

(2) 無増悪生存期間 (progression free survival: PFS)

登録日を起算日とし，増悪と判断された日，またはあらゆる原因による死亡日のうち早いものまでの期間。PFS の判定には，以下の規定を定める。

① 「増悪 progression」とは，総合効果判定 {3.8(5) 時点総合効果の判定基準} における増悪と，画像診断に依らない原病の増悪 {3.8(7)臨床的増悪} の両者を含む。画像診断に基づいて判断した場合はその検査日を増悪日とし，臨床的増悪の場合は臨床的判断日を増悪日とする。

② 増悪と判断されていない生存例では，増悪がないことが確認された最終日 (最終無増悪生存確認日)をもって打ち切りとする。

③ 増悪の診断が画像診断による場合，「画像上疑い」の検査日ではなく，後日「確診」が得られた画像検査の「検査日」をもってイベントとする。画像診断によらず臨床的に増悪と判断した場合は，増悪と判断した日をもってイベントとする。

④ 二次がん，異時性重複がん，異時性多発がんの発生はイベントとも打ち切りともせず，他のイベントが観察されるまで無増悪生存期間とする。

⑤ 特性や患者拒否などの理由による治療中止例で，後治療として他の治療が加えられた場合も，イベントと打ち切りは同様に扱い，治療中止時点や後治療開始日で打ち切りとはしない。

(3) 全生存期間(overall survival: OS)

登録日を起算日とし，あらゆる原因による死亡日までの期間。 生存例では最終生存確認日をもって打ち切りとする。

追跡不能例では追跡不能となる以前で生存が確認されていた最終の日付をもって打ち切りとする。

(4) 治療成功期間(time to treatment failure : TTF)

登録日を起算日とし，増悪と判断された日，あらゆる原因による死亡日，プロトコール治療中止日のうち，もっとも早いものまでの期間。

① プロトコール治療中止日は中止と判断した日とする。

② 「増悪 progression」は総合効果判定｛3.8(5) 時点総合効果の判定基準｝における増悪と，画像診断に依らない原病の増悪｛3.8(7)臨床的増悪｝の両者を含む。画像診断に基づいて判断した場合はその検査日を増悪日とし，臨床的増悪の場合は臨床的判断日を増悪日とする。

③ 増悪の診断が画像診断による場合，「画像上疑い」の検査日ではなく，後日確診が得られた画像検査の検査日をもってイベントとする。画像診断によらず臨床的に増悪と判断した場合は，増悪と判断した日をもってイベントとする。

④ 継続してプロトコール治療が実施され，かつ増悪がない場合は最終生存確認日（最終無増悪生存確認日)をもって打ち切りとする。

(5) 化学療法開始までの期間(time to chemotherapy: TTC)

登録日を起算日とし，最初に化学療法薬が投与された日までの期間。

① 化学療法開始前の死亡例は，死亡日をもって打ち切りとする。

② 化学療法の実施が確認できない追跡不能例では，追跡不能となる以前で生存が確認されていた最終の日付をもって打ち切りとする。

(6) 奏効率（response rate : RR)

全治療例のうち測定可能病変を有する対象集団における，最良総合効果｛3.8(6)最良総合効果｝が CR または PR のいずれかである患者の割合を奏効率とする。

(7) 健康関連 quality of life(health-related quality of life: HRQoL)

（5.5 健康関連 Quality of life の調査を参照）

(8) 有害事象(Toxicity)

全治療例を対象とし，登録時からプロトコール治療中止までに認められた有害事象を評価する(19 有害事象の評価と報告を参照)。

4 研究の目的及び意義

4.1 研究の目的

本観察研究の目的は以下である。

(1) 一次内分泌療法に良好な臨床効果の得られなかったエストロゲン受容体陽性 HER2 陰性の閉経後転移乳がん(一次内分泌療法低感受性乳がん)に対する，二次内分泌療法全般，および薬剤種別の有効性と安全性を明らかにする。

(2) 前内分泌療法への反応性(術後内分泌療法開始から再発までの期間，あるいは転移乳がんに対する一次内分泌療法の無増悪期間)，および腫瘍の生物学的特性(ER の発現強度，PgR の発現の有無)が，二次内分泌療法の効果に及ぼす影響を明らかにし，Hortobagyi の治療アルゴリズムを補完する情報を得る。

4.2 研究の着想に至った背景

(1) 対象疾患

国立がん研究センターがん情報サービスによると，2011 年の日本人女性の乳がん年間罹患患者数の全国推定値は約 72，500 人，罹患割合(人口 10 万対)は 110.5 と女性の悪性腫瘍罹患臓器として第 1 位であった。2013 年の年間死亡数は 13，148 人で，女性の悪性腫瘍による死亡原因のうち，胃がん，結腸・直腸がん，肺がん，膵がんに続いて第 5 位である。

日本人女性の乳がん罹患率は一貫して増加傾向にあり，結腸・直腸がんや肺がんとならんで上位を占めている[4)]。

乳がんと診断された患者の約 9 割には原発巣に対して外科的切除が行われるが，残りの 1 割は初診時に明らかな遠隔転移を有する。

乳がんは早期発見および外科的切除が行われた場合の生存率は良好であるが，初診時に臨床的に検出することができない微小転移の存在が予後を左右するとされ，手術を実施した患者の約 6 割では治癒が得られる一方で，約 4 割は再発をきたす[5)]。

一旦転移再発を来たした乳がんの，生命予後の中央値は 28 ヶ月であり，現代の治療をもっても治癒は難しい。

(2) 対象集団の治療

① 転移乳がんの治療目標

初診時に既に手術適応外の遠隔転移を有する乳がん(stage IV)，および遠隔転移による再発を来たした乳がんでは，治癒を得ることは極めて稀であり，治療の主な目的は症状の緩和，延命，Quality of Life(QOL)の維持と改善である[6, 7)]。

転移乳がんの治療は薬物療法を主体とし，必要に応じて放射線，外科療法などを組み合わせ，症状の緩和と日常生活の維持を目標とする。

② 転移乳がんに対する内分泌療法

近年，乳がんの薬物治療では，がんの生物学的特性に基づいた治療選択が重視されて

いる。乳がんではサブタイプとも呼ばれ，エストロゲン受容体（Estrogen receptor: ER）やHER2(human epidermal growth factor receptor type2)の発現は乳がんの重要な予後予測因子であるとともに，内分泌療法，化学療法，分子標的剤などの効果予測因子となることから，サブタイプ分類に基づいた治療選択が実施されている。

さらに治療ストラテジーとして Hortobagyi の提唱したアルゴリズムが実地臨床で実践されている。ER 陽性転移乳がんに対しては，生命予後を左右する遠隔転移がなければ内分泌療法から治療を開始し，効果が見られなくなり次第別の治療に移行するものである。一次内分泌療法に反応する症例では，効果がなくなっても二次内分泌療法には再度抗腫瘍効果が期待できるので，内分泌療法を継続することが基本である[8)]。

③ 内分泌療法への感受性や耐性に関する近年の見解

近年，内分泌療法への感受性や耐性機序に関して，初期の内分泌療法に伴う臨床経過を基盤として，いくつかの分類モデルが提唱されている。

再発乳がんの国際コンセンサス会議として開催された ABC2 では，ER 陽性転移乳がんの内分泌療法に対する耐性を，初期内分泌療法の開始から再発，あるいは増悪するまでの期間によって以下のように分類することを提唱している[9)]。

【一次内分泌療法耐性乳がん】

- 術後の補助内分泌療法を開始して2年以内に再発した症例。
- 転移乳がんに対する初回内分泌療法を開始し，開始時点から 6 ヶ月以内に病勢進行を示す症例。

【二次内分泌療法耐性乳がん】

- 術後の補助内分泌療法を開始して2年以降，あるいは内分泌療法の終了後 12 ヶ月以内に再発した症例。
- 転移乳がんに対する初回内分泌療法を開始し，開始時点から 6 ヶ月以降に病勢進行を示す症例。

その他，薬剤の感受性の観点からの分類として，術後補助内分泌療法開始から2年以内に再発した症例，あるいは初回内分泌療法を開始後3ヶ月以内に増悪したものを薬剤感受性“very low”， 術後補助内分泌療法中の再発ではあるが，開始して2年以降に再発した症例，あるいは初回内分泌療法を開始後3～9 ヶ月以内に増悪したものを薬剤感受性“low”とする分類も提唱されている（図 1)[9)]。

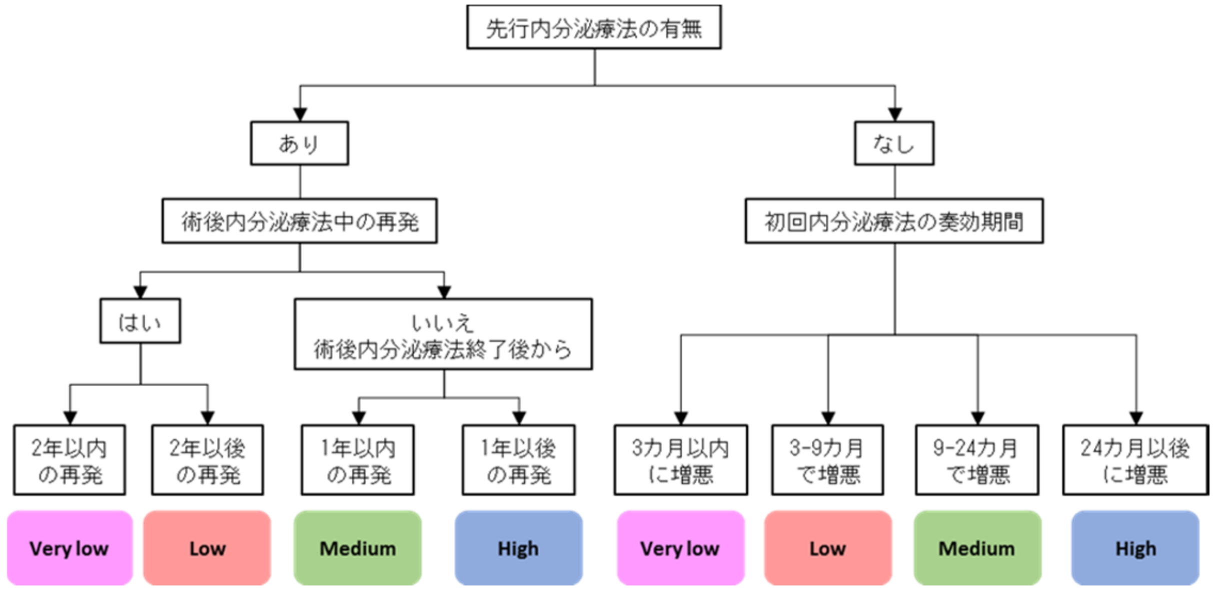

図1：ABC2 で提唱された，先行内分泌療法の効果による感受性の分類

④　近年の内分泌療法薬の有効性に関する報告

近年，乳がんの薬物療法剤では従来の作用機序とは異なる薬剤が使用可能となった。

フルベストラントは，タモキシフェンに認められるようなERに対する部分アゴニスト作用は有しておらず，乳がん細胞において ER を down regulate する効果を持つことから，Selective Estrogen Receptor downregulator（SERD）に分類される[10-12]。

転移乳がんの一次内分泌療法として，アナストロゾールとフルベストラントを比較した第Ⅱ相比較試験（First-Line Study Comparing Endocrine Treatments [FIRST] trial）の結果から，フルベストラントはアナストロゾールと同等の臨床的有用率を示し，
転移乳がんに対する有効性が示されている[13]。

また，エベロリムスは mTOR（mammalian target of rapamycin）阻害剤であり，細胞増殖を制御する代表的シグナル経路，PI3K/AKT 経路の下流に位置するmTORを阻害することによりがん細胞の増殖効果を示し，内分泌療法耐性となった乳がんに対し，他の内分泌療法薬と併用投与することで，無増悪生存期間を延長することが示されている[14-16]。

これら薬剤の登場により，一次内分泌療法へ十分な効果が認めらなかった症例に対しても，二次内分泌療法により臨床的に意味のある治療効果の得られることが期待される。

(3) 研究計画

今後，一次内分泌療法へ十分な効果が認めらなかった症例に対する標準治療の確立には，これらを対象集団としたランダム化比較試験での検証が必要である。しかしこれまでに一次内分泌療法へ十分な効果が認めらなかった症例を対象として，二次内分泌療法の有効性と安全性を評価した臨床研究はない。さらに，現在では乳がん二次内分泌療法薬として選択可能な薬剤は多種多様にわたる。

今後，これらを対象とした比較臨床試験を計画するためには，二次内分泌療法の適応となる対象群の選定と根拠，二次内分泌療法全般・および薬剤種毎に予想される治療効果等の基盤情報が必要である。

以上を背景として，本観察研究を計画した。

本研究では一次内分泌療法に良好な臨床効果の得られなかった閉経後エストロゲン受容体陽性 HER2 陰性の転移乳がん患者を対象集として，これらに対する内分泌療法薬を用いた二次内分泌療法施行例を前向きに観察し，その有効性と安全性を，臨床的有用率，無増悪生存期間，全生存期間，治療成功期間，化学療法までの期間，奏効率，健康関連 quality of life，有害事象を指標として明らかにする。

また同時に，現在本邦にて使用可能な多種にわたる内分泌療法薬を，その作用機序から分類し，各々の有効性と安全性に関しても検証する。

本研究では，前内分泌療法に対して低感受性を示した症例を対象とするが，これらに対する二次内分泌療法の反応性は一律ではないと予想される。前述したように，二次内分泌療法を選択するか，あるいは化学療法を選択するべきかは，薬剤の選択とともに極めて重要な臨床上の課題である。本研究では二次内分泌療法への反応性に影響を及ぼす可能性のある効果予測因子の検証も目的の一つとする。本研究で検証しようとする効果予測因子は①前内分泌療法への反応性（術後内分泌療法開始から再発までの期間，あるいは転移乳がんに対する一次内分泌療法の無増悪期間），②腫瘍の生物学的特性（ER の発現強度，PgR の発現の有無）である。

4.3 研究仮説

本研究では目的を明らかにするため，以下の研究仮説を設定し検証する。

（1） 一次内分泌療法低感受性乳がんに対しても，近年の内分泌療法薬により30%以上の臨床的有用率が期待できる。

（2） 一次内分泌療法低感受性乳がんに対する二次内分泌療法の治療効果には，前治療への反応性や腫瘍の生物学的特性が影響し，二次内分泌療法の効果を予測できる。

4.4 研究の意義

乳がんのサブタイプの内，およそ 70%が ER 陽性乳がんであり大多数を占める。よって転移乳がんにおけるサブタイプでも，ER 陽性乳がんの占める割合は大きい。

ER 陽性転移乳がんの治療では，Hortobagyi のアルゴリズムを踏襲した内分泌療法が日常的に実施されているが，一次内分泌療法低感受性乳がんに対する，至適な次治療に関するエビデンスは存在しない。

本研究により，一次内分泌療法低感受性乳がんに対する二次内分泌療法の有効性と安全性を明らかにすることで，今後の治療選択に有益な情報を得ることができる。

さらに，解決すべき臨床上の課題を明確とし，今後の臨床研究を計画する上で，基盤となる有益な情報を得ることができる。

過去に一次内分泌療法低感受性乳がんを対象とした臨床研究はなく，独創性の観点からも本研究の意義は大きい。

5　研究の方法及び期間

5.1　研究デザイン

本研究の計画と実施は，文部科学省及び厚生労働省より平成２６年１２月２２日に公布された「人を対象とする医学系研究に関する倫理指針」を遵守する（Appendix A）。

本指針で示されている「介入」と「侵襲」の以下の解釈により，
本研究は「軽度な侵襲を伴う，非介入研究」に相当し，研究デザインは観察研究と位置づけられる。

(1)　侵襲

指針の第1章，第2 「用語の定義」，（2）では侵襲を，
『研究目的で行われる，穿刺，切開，薬物投与，放射線照射，心的外傷に触れる質問等によって，研究対象者の身的又は精神に障害又は負担が生じることをいう。侵襲のうち，研究対象者の身体及び精神に生じる障害及び負担が小さいものを「軽微な侵襲」という。』
と定義されている。

本研究で実施する治療は，保険承認された薬剤を，患者と医療者の選好に基づき実施するため，定義にある「研究目的」の治療には相当しない。

本研究で実施する調査計画では質問票による Quality of life（QoL）の評価を実施する。医学系研究における QoL の調査は，妥当性と信頼性の検証された尺度を用いて実施され，すでに医学研究の評価方法として一般化している。尺度の妥当性検証の過程には，患者の精神的あるいは身体的な負担への配慮（内容妥当性）も含まれており，QoL 評価により研究対象者の精神に障害を及ぼすことはないものと考えられる。本研究での QoL 評価への回答には1回につき15分程度を要し，登録時，1ヵ月目，3ヵ月目の合計3回の評価を実施するため，軽度の負担を要する。

以上より，研究参加に伴う研究対象者の時間的及び身体・心理的な負担は小さいものと考えられ，本研究に参加した研究対象者への侵襲は「軽微な侵襲」に相当する。

(2)　介入

指針の第1章，第2項「用語の定義」，（3）では介入を，
『研究目的で，人の健康に関する様々な事象に影響を与える要因(健康の保持増進につながる行動及び医療における傷病の予防，診断又は治療のための投薬，検査等を含む。)の有無又は程度を制御する行為（通常の診療を超える医療行為であって，研究目的で実施するものを含む。)をいう。』
と定義されている。

本研究で実施する治療は，保険承認された薬剤を，患者と医療者の選好に基づき日常臨床として実施するため，定義にある「研究目的」の治療には相当しない。

本研究では調査として，登録時，3ヵ月目，6ヵ月目の合計3回の画像評価を規定しているが，これらは転移乳がんに対する薬物療法の評価手法として，通常の診療を超える医療行為に相当しない。また，QoL 評価は医療行為に相当しない。

以上より，本研究は介入を伴わない医学系研究であり，観察研究に位置づけられる。

5.2 症例の登録

(1) 「症例登録票」の送付

担当医師は，研究対象者が選択基準（6.2）に該当することを確認した後，症例登録票(Appendix B)を全て記入の上，データセンターへ FAX 送信する。

NPO 日本臨床研究支援ユニット　CSPOR データセンター<br>FAX：03-5298-8536　　電話：03-3254-8029<br>受付時間：平日 10 時～17 時(祝祭日，土曜・日曜，年末年始は受け付けない)

(2) 登録の確認

データセンターは受領した症例登録票に基づき研究対象者の適格性を確認し，登録する。

① 症例登録票の記載が不十分な場合は，登録されない。

② 登録日は一連の登録手続きが完了した日とし,「症例登録確認通知書」(Appendix C)に記載される。データセンターに症例登録票を FAX した時点では「登録」とならない。

③ データの研究利用の拒否があった場合を除き，一度登録された研究対象者は，登録取り消し（データベースから抹消）されない。重複登録の場合は，初回の登録情報(登録番号)を採用する。

④ 誤登録・重複登録が判明した場合，担当医師は速やかにデータセンターに連絡する。

(3) 「症例登録確認通知書」の送付

データセンターは，症例登録票に記載された担当医師宛に「症例登録確認通知書」をFAX 送信する。

5.3 治療

(1) 治療の選択

本研究において，有効性と安全性を評価しようとする治療とは，対象集団に対して日常臨床として実施されている二次内分泌療法全般であるが，本研究で選定した対象集団に対する，内分泌療法薬の選択肢は多岐にわたり，至適な薬剤は確立されていない。

したがって，対象集団に対する治療の選択は，前治療で使用された内分泌療法薬を除き，本邦にて閉経後の乳がん患者に対する内分泌療法薬として保険上認められている薬剤全般となる｛5.3 (2)の表を参照｝。

本研究では,治療の選択は医療者と患者との話し合いに基づき実施する(treatment of physician’s choice: TPC)。また，本研究は観察研究であることから，併用治療の禁止は規定しない。ただし，登録時に予定した以外の抗腫瘍効果を有する薬剤の使用(化学療法剤，分子標的薬の併用や他の内分泌療法)，その他の治療の併用（手術，放射線治療)はガイドラインに準じて実施することを推奨する[8)]。またこれらの治療を，内分泌療法と併用して実施した場合は，経過報告書にその詳細を記載することとする。

本研究への参加にあたっては，症例登録票に予定治療を明記する。

以下，本研究で有効性と安全性の評価対象とする予定治療を「プロトコール治療」と呼ぶ。

プロトコール治療は登録後 2 週以内に開始することとし，2 週以内に開始できなかった場合はその理由を「経過報告書 (Appendix D)」に記録する。

プロトコール治療の薬剤情報は，使用薬剤の添付文書を参照とする。

（最新の添付文書は「医薬品医療　機器情報提供ホームページ(http://www.info.pmda.go.jp/)」で確認可能である。

(2) 内分泌療法薬

本邦で閉経後乳がん患者に対する内分泌療法薬として承認されている薬剤の, 薬品名及び用法･用量を下表に示す。

分類	薬品名	用法・用量
選択的エストロゲン受容体モジュレーター (Selective Estrogen Receptor Modulators: SERMs)	タモキシフェン	通常, 1日 20mg を1～2回に分割経口投与する。なお, 症状により適宜増量できるが, 1日最高量はタモキシフェンとして 40mg までとする。
	トレミフェン	通常, 成人にはトレミフェンとして 40mg を1日1回経口投与する。また, 既治療例 (薬物療法及び放射線療法などに無効例) に対しては, 通常成人にトレミフェンとして 120mg を1日1回経口投与する。なお, 症状により適宜増減する。
アロマターゼ阻害剤 (Aromatase inhibitor: AI)	アナストロゾール	通常, 成人にはアナストロゾールとして 1mg を 1 日 1 回, 経口投与する。
	レトロゾール	通常, 成人にはレトロゾールとして1日1回 2.5mg を経口投与する。
	エキセメスタン	通常, 成人にはエキセメスタンとして 1 日 1 回 25mg を食後に経口投与する。
選択的エストロゲン受容体ダウンレギュレーター (Selective Estrogen Receptor Downregulator: SERD)	フルベストラント	通常, 成人にはフルベストラントとして 500mg を, 初回, 2 週後, 4 週後, その後 4 週ごとに 1 回, 左右の臀部に 1 筒ずつ筋肉内投与する。
黄体ホルモン製剤	メドロキシプロゲステロン酢酸エステル	メドロキシプロゲステロン酢酸エステルとして通常成人 1 日 600～1200mg を 3 回に分けて経口投与する。

(3) mTOR (mammalian target of rapamycin) 阻害剤の併用

ER 陽性かつ HER2 陰性で非ステロイド性アロマターゼ阻害剤（レトロゾール又はアナストロゾール）に抵抗性の局所進行性又は転移性の閉経後乳癌患者を対象とした第Ⅲ相国際共同臨床試験（日本を含めた世界 24 ヵ国で実施された二重盲検比較試験）で, プラセボ＋エキセメスタン群と比較しエベロリムス＋エキセメスタン群で有意な無増悪生存期間の延長が報告されている [15, 16)]。

また, アロマターゼ阻害剤に抵抗性の局所進行性又は転移性の閉経後乳がん患者を対象とした第Ⅱ相ランダム化試験では, タモキシフェン群に比較し, エベロリムス＋タモキシフェン群で有意な増悪までの期間, 全生存期間の延長が認められている [17)]。

本邦では, 手術不能又は再発乳がんに対して, 内分泌療法薬との併用においてエベロリムスの投与が認められている。よって, 本研究では 5.3 (2)に列挙した内分泌療法薬とエベロリムスとの併用は, 治療選択肢の一つと考えられる。

エベロリムスと内分泌療法薬との併用は, 有効性や有害事象に影響を及ぼし得る要因と考えられるため, 併用薬として経過報告書に記載する。

(4) 解析に用いる薬剤種類別の分類

現在，術後内分泌療法，および転移乳がんに対する一次内分泌療法の第一選択薬はアロマターゼ阻害剤である。よって，本研究に登録される症例の大半が，前治療としてアロマターゼ阻害剤の投与を受けていると予測される。

アロマターゼ阻害剤の次治療として選択できる薬剤は多数あるが，その主たる作用機序から以下のように分類できる。本研究では二次内分泌療法全般とともに，以下の分類を用いた有効性と安全性の評価を行う。なお，以下の others に分類された対象群は，解析結果の解釈が困難であるため，単独での解析対象としない。

- コホート SERMs
  Selective Estrogen Receptor Modulators: SERMs であるタモキシフェン，トレミフェンが選択された場合。

- コホート AIs
  Aromatase inhibitor: AI であるアナストロゾール，レトロゾール，エキサメスタンが選択された場合。

- コホート SERD
  Selective Estrogen Receptor Downregulator: SERD であるフルベストラントが選択された場合。

- コホート with mTORi
  内分泌療法薬に mTOR 阻害剤であるエベロリムスの併用が選択された場合。
  （併用内分薬の種類は問わず，エベロリムスが併用された場合は，本コホートに分類する）

- Others
  選択された治療が，上記のいずれのコホートにも分類できない場合。

(5) 併用治療・併用薬剤について，及び経過報告書への記載事項

① ガイドラインに準じた治療

本研究への参加による，併用禁止治療，薬剤は規定しないが，ガイドラインに準じた標準治療を実施することを推奨する[8)]。

以下の治療や薬剤を，登録時以後にあらかじめ報告されたプロトコール治療に併用して実施した場合は，その実施理由（病勢の増悪やプロトコール治療の有害事象による中止の有無）を経過報告書に記載する。

A) 乳がん病巣に対する手術療法

B) 乳がん病巣に対する放射線療法

C) mTOR 阻害剤以外の分子標的薬

D) 化学療法

E) 免疫療法

F) その他，乳がんへの抗腫瘍活性が認識されている薬剤の使用

② その他，経過報告書への記載が必要な治療・薬剤

転移乳がんの治療薬として併用されることの多い以下の薬剤は，経過報告書に併用の有無を記載する。実施理由の記載は要しない。

G) 骨転移に対する治療を目的としたビスフェオスフォネート製剤の使用

H) 骨転移に対する治療を目的とした抗 RANKL 抗体の使用

③ 経過報告書への記載の必要のない治療・薬剤

以下の併用薬・併用治療は，経過報告書への記載を要しない。

I) 既往症に対する治療薬

J) 症状の緩和や，有害事象に対する治療を目的とした治療薬

(6) プロトコール治療終了後の治療選択，及び経過報告書への記載事項

プロトコール治療終了後の治療選択は規定しない。

本研究では転移乳がんに対する化学療法開始日までの期間を評価項目とするため，

プロトコール治療終了後に化学療法を実施した場合は，投与開始日と投与した化学療法の種類を調査し，経過報告書に記載する。

5.4 調査

(1) 調査項目

本研究では，対象者に対するプロトコール治療の有効性と安全性評価のため，以下の調査を実施する。

① 病歴
② 身体所見
③ 乳がん病巣の評価のための画像診断
④ 有害事象の調査
⑤ 治療の調査：選択薬と選択理由，コンプライアンス，併用薬・併用治療
⑥ 健康関連 Quality of life（Health-related Quality of Life: HRQoL）の調査
⑦ 後治療の調査
⑧ 予後調査

本研究で実施する①－⑤の調査は患者の負担に十分留意し，日常臨床の範疇の範囲で実施するよう，以下 5.4 (2)のように定める。

また，健康関連 QoL の調査では，研究対象者の負担に配慮し，1 回の調査に要する時間を通常 15 分以内，調査回数も必要最小限とする（登録時，プロトコール治療開始後1，3ヶ月目の合計3回）。

プロトコール治療開始から 6 ヶ月目以後の調査は，後治療，予後調査を除き実施しない。

(2) 調査スケジュール

下表のスケジュールで調査を実施する。

調査項目	登録まで	プロトコール治療開始から 1 ヶ月目	プロトコール治療開始から 3 カ月目	プロトコール治療開始から 6 カ月目
病歴	○			
身体所見[注1]<br>身長・体重・PS	○		○	○
画像評価[注2]	○[注3]		○[注4]	○[注4]
有害事象	○		○	○
治療の調査	○		○	○
健康関連 QoL	○	○[注4]	○[注4]	
後治療の調査		プロトコール治療中止後に調査する[注5]		
予後調査				

注 1：体重測定は登録時のみ

注 2：画像検査は，原則として登録までに実施した検査と同一手法で実施すること

注 3：登録前 28 日以内（4 週間以内）に評価する

注 4：規定日の前後 2 週間内に評価することを推奨する

注 5：プロトコール治療終了後から 1 年毎

(3) 登録までに必要な調査

担当医は症例の登録までに，以下の調査を実施する。

① 病歴

A) 原発病巣に関する項目

- 手術歴の有無と手術日
- 臨床病期分類(3.3 臨床病期分類を参照)
- 組織型(3.4 組織学的分類を参照)
- 組織学的リンパ節転移個数
- ホルモン受容体(ER, PgR)の発現
- HER2 の発現状況

B) 前治療歴

- 術前・術後の化学療法実施の有無と種類，最終投与日
- 術後内分泌療法の種類と開始日，最終投与日
  あるいは，一次内分泌療法の種類と開始日，最終投与日
- 再発が確認された日
  あるいは，増悪が確認された日
- 再発後の放射線治療歴の有無と最終照射日
- 再発後の化学療法歴の有無（無いことの確認）
- 再発転移の認められた臓器

C) 既往歴

② 身体所見

A) 身長

B) 体重

C) Performance Status

③ 乳がん病巣の評価のための画像診断

以下の項目を登録前 28 日以内(4 週間以内)に評価する

A) 胸部 CT, MRI もしくは胸部 X 線(必須)

B) 腹部 CT, MRI もしくは腹部超音波(必須)

C) 臨床的に骨転移が疑われる場合は骨シンチ

＊骨シンチで骨転移が疑われる場合は骨のレントゲン撮影, もしくは CT , MRI などで病変を評価する

D) 臨床的に脳転移が疑われる場合, 脳 CT または MRI

E) リンパ節や局所皮膚再発病巣の評価と記録

＊視診:指標を貼付した写真で経過観察することが望ましい。

＊触診:表在リンパ節, 皮膚転移等の直接病巣の大きさが計測可能なものは体外計測を行う。計測可能な場合は CT や超音波で評価することが望ましい。

④ 有害事象の調査

有害事象共通用語規準 v4.0 日本語訳 JCOG 版に従い, Grade3 以上の有害事象の有無を確認し, 記録する。

乳がん患者, および内分泌療法に伴うことの多い症状として特に下記の有害事象の有無を確認し, 記録する。

下記以外の症状に関しても, Grade3 以上の有害事象を認めた場合には, その他の有害事象として記録する。

- 便秘
- 下痢
- 口腔粘膜炎
- 悪心
- 嘔吐
- 倦怠感
- 疼痛
- 関節痛
- 不眠
- 膣分泌物
- 膣乾燥
- ほてり

⑤　治療の調査

併用薬(ビスフォスフォネート製剤，抗 RANKL 抗体使用)の有無

投与予定の内分泌治療薬，治療開始予定日，及び選択した理由

⑥　HRQoL の調査(詳細は 5.5 健康関連 quality of life の調査を参照)

HRQoL 評価のため，下記の調査票(尺度)を用いる。

HRQoL の評価は，登録までに終了していることが望ましいが，時間的な余裕がないなどの理由で調査が終了していない場合は，プロトコール治療が開始される前日までに患者の記入を終了しておくこと。

A)　社会的背景:学歴，就労状態，家計収入，パートナーの有無

B)　HRQoL の予期

C)　FACT (Functional Assessment of Cancer Therapy)- ES (Endocrine Symptom), B(Breast):がん臨研究で一般的に用いられる QOL 尺度 FACT-G(General, 日本語版 29 項目)の追加尺度として ES(内分泌関連症状，18 項目)，B(乳癌，9 項目)

(4) **プロトコール治療開始後，1ヶ月目**の調査計画

プロトコール治療開始日から1カ月目に以下の調査を実施する。

① HRQoLの調査（詳細は5.5 健康関連 quality of life の調査を参照）

以下の調査をプロトコール治療の開始から1カ月目に実施する。

＊調査は規定日の前後2週間内に実施することを許容する。

A) FACT- ES, Breast

B) Minimally important difference を同定するための推移の質問

(5) **プロトコール治療開始後の 3 ヶ月目**の調査計画

プロトコール治療開始日から 3 カ月目に以下の調査を実施する。

① 身体所見

＊調査日は規定日の前後 2 週間を許容する。

A) 体重

B) Performance Status

② 乳がん病巣の評価のための画像診断

＊登録前に実施した検査と同一の手法での評価が望ましい。

＊検査日は規定日の前後 2 週間に実施することを推奨する。

A) 胸部 CT，MRI もしくは胸部 X 線

B) 腹部 CT，MRI もしくは腹部超音波

C) 臨床的に骨転移が疑われる場合は骨シンチ

＊骨シンチで骨転移が疑われる場合は骨のレントゲン撮影，もしくは CT ，MRI などで病変を評価する

D) 臨床的に脳転移が疑われる場合，脳 CT または MRI

E) リンパ節や局所皮膚再発病巣の評価と記録

＊視診：指標を貼付した写真で経過観察することが望ましい。

＊触診：表在リンパ節，皮膚転移等の直接病巣の大きさが計測可能なものは体外計測を行う。計測可能な場合は CT や超音波で評価することが望ましい。

③ 有害事象の調査

過去3カ月に生じた Grade3 以上の有害事象の有無を確認し，記録する。

＊調査日は規定日の前後 2 週間を許容する。

調査内容は，5.4 (3)登録までに必要な調査の④と同様。

④ 治療状況の調査

A) プロトコール治療中止の有無，中止となった場合は中止の理由と中止日

B) 過去 3 カ月間のプロトコール治療のコンプライアンス。

C) 過去 3 カ月間のビスフォスフォネート製剤，抗 RANKL 抗体使用の有無。

D) 過去 3 ヶ月間に併用実施された治療（乳がん病巣に対する手術療法，乳がん病巣に対する放射線療法，mTOR 阻害剤以外の分子標的薬，化学療法，免疫療法，その他乳がんへの抗腫瘍活性が認識されている薬剤）の有無と実施理由。

⑤ HRQoL の調査 ｛5.4 (4)①と同一｝

(6) **プロトコール治療開始後，6ヶ月目**の調査計画

プロトコール治療開始日から6カ月目に以下の調査を実施する。

＊ただし，プロトコール治療開始後の3ヶ月目の調査で，プロトコール治療の中止，増悪が報告された場合は，6ヶ月目の調査は不要であり，(7)の調査に移行する。

① 身体所見

＊調査日は規定日の前後2週間を許容する。

A) 体重

B) Performance Status

② 乳がん病巣の評価のための画像診断

＊登録前に実施した検査と同一の手法での評価が望ましい。

＊検査日は規定日の前後2週間に実施することを推奨する。

A) 胸部CT，MRIもしくは胸部X線

B) 腹部CT，MRIもしくは腹部超音波

C) 臨床的に骨転移が疑われる場合は骨シンチ

＊骨シンチで骨転移が疑われる場合は骨のレントゲン撮影，もしくは CT ，MRI などで病変を評価する

D) 臨床的に脳転移が疑われる場合，脳CTまたはMRI

E) リンパ節や局所皮膚再発病巣の評価と記録

＊視診：指標を貼付した写真で経過観察することが望ましい。

＊触診：表在リンパ節，皮膚転移等の直接病巣の大きさが計測可能なものは体外計測を行う。計測可能な場合はCTや超音波で評価することが望ましい。

③ 有害事象の調査

前回の調査以後～調査日に生じたGrade3以上の有害事象の有無を確認し，記録する。

＊調査日は規定日の前後2週間を許容する。

調査内容は，5.4 (3)登録までに必要な調査の④と同様。

④ 治療状況の調査

A) プロトコール治療中止の有無，中止となった場合は中止の理由と中止日

B) 過去3カ月間のプロトコール治療のコンプライアンス。

C) 過去3カ月間のビスフォスフォネート製剤，抗RANKL抗体使用の有無。

D) 過去3ヶ月間に併用実施された治療（乳がん病巣に対する手術療法，乳がん病巣に対する放射線療法，mTOR阻害剤以外の分子標的薬，化学療法，免疫療法，その他乳がんへの抗腫瘍活性が認識されている薬剤）の有無と実施理由。

(7) **プロトコール治療開始後の 6 ヶ月以降，あるいはプロトコール治療中止後の調査計画**

① 調査時期

A) プロトコール治療開始後 6 ヶ月の時点で，プロトコール治療が継続して実施されていた場合は，プロトコール治療開始後 6 ヶ月の時点から1年毎

B) プロトコール治療開始後 6 ヶ月の時点で，プロトコール治療が中止となった場合には，プロトコール治療が中止となった日から1年毎

② 調査の内容

A) プロトコール治療の継続状況：プロトコール治療継続の有無，中止となった場合は中止日と中止となった理由{5.4 (7) ①の A)に該当する対象者のみ}

B) 増悪の調査：3.9 (2)で定義された増悪の有無。増悪が認められた場合は，増悪と判断された日。(すでに増悪が報告された対象者では不要)

C) 後治療の調査：化学療法実施の有無，実施された場合は，化学療法の実施日と種類。

D) 予後調査：最終生存確認日，死亡した場合は死亡日と死亡原因。

(8) 調査の中止

以下のいずれかに該当する場合には調査を中止する。

① 転院等の理由によりそれ以降の調査継続が不可能と判断された場合

② 同意を撤回した場合

③ 死亡した場合

④ 適格基準を満たさないことが判明した場合

⑤ その他，本研究の対象として継続不可能と判断された場合

5.5 健康関連 Quality of life の調査

（1） HRQoL 評価の背景

健康関連 quality of life （health-related quality of life: HRQoL）は，一般に疾病ならびに治療が健康や生活全般に及ぼす影響と定義される[18)]。HRQoL は通常，機能面，身体面，心理面，社会面といった基本ドメインから構成された多領域の概念であり，評価視点としては患者自身の主観性が重視されている。近年，個々の症状や兆候といった単領域の関心事項においても，医療者による客観的評価と患者の主観的評価には大きな乖離があることが明らかとなり，単領域の評価においても患者の主観性が重視されるようになった[19)]。これらを背景に，医療の分野では HRQoL や症状を含め，より包括的な主観的評価概念として patient-reported outcomes （PROs）の概念が確立された。また，PROs 概念の確立に随伴し，PROs を測定するための信頼性と妥当性が検証された様々な尺度が開発されている。2012 年，ASCO は "Recommendations for incorporating patient-reported outcomes into clinical comparative effectiveness research in adult oncology"を公表し，成人期のがん臨床比較試験，特に治癒の困難な転移がんの臨床試験において，患者の意思決定に役立つ情報を得るためには PROs の評価は必須であるとしている[20)]。本試験の対象となる転移乳がんでは，現代の治療をもっても治癒は困難であり，治療の目的は生存期間の延長，HRQoL の維持と改善，症状の緩和であるとされている[21)]。

（2） HRQoL 評価の目的

本研究の主たる目的は，一次内分泌療法低感受性乳がんに対する近年の内分泌療法の有効性と安全性を評価することである。QoL の維持と改善は，転移乳がんの治療目的の一つであり，有効性の評価のためには QoL の評価が必須である。

　通常 HRQoL は，調査票により得られたスコア値の差や変動を，統計解析することにより議論されることが多いが，本来得られたスコア値の差や変動に内在する臨床的な意義を解釈することが極めて重要である。近年，HRQoL スコアの解釈する指標として，minimally important difference（MID）が重視されている。MID とは臨床的に意味のある最小のスコア値の差を意味しており，がん臨床研究の中で MID を検証することは，臨床試験における HRQoL 評価全般の発展には不可欠の事項である。

以上より，本研究における HRQoL 評価の目的は以下とする。

① 臨床的に意味のある HRQoL の維持率を指標として，一次内分泌療法に良好な臨床効果の得られなかったエストロゲン受容体陽性 HER2 陰性の閉経後転移乳がん（一次内分泌療法低感受性乳がん）に対する，二次内分泌療法が HRQoL に及ぼす影響を明らかにする。

② 本研究対象者から経時的に得られた HRQoL スコアの変動に着目し，スコア値に内在する臨床的意義を，転移乳がんの内分泌療法に伴う minimally important difference（MID）として明らかにする。

(3) HRQoL 尺度

以下の尺度を用いる（Appendix F）。

なお登録時には，その後の HRQoL に及ぼす可能性のある，患者の社会的背景と QoL の予期に関する調査を行う。

① FACT (Functional Assessment of Cancer Therapy)- ES (Endocrine Symptom), B(Breast)[22-24]：がん臨床試験で一般的に用いられる QOL 尺度 FACT-G（General，日本語版 27 項目）の追加尺度として ES（内分泌関連症状，19 項目），B（乳癌，10 項目）

② 推移の質問：MID（Minimally important difference）の同定に必要な主観的アンカー情報として用いる。

(4) 調査スケジュール

調査項目	登録時[注1]	プロトコール治療開始から 1 カ月目[注2]	プロトコール治療開始から 3 カ月目[注2]
社会的背景	○		
QoL の予期	○		
FACT-G, B, ES	○	○	○
推移の質問		○	○
医療者への欠測理由の調査	調査予定時期を超え，当該対象患者から調査票の返送がない場合		

注 1：登録時の調査は，同意取得から登録までに終了することが望ましい。

時間的な余裕がない場合は，プロトコール治療を開始するまでに調査を終了すること。

注 2：規定日の前後 2 週間の調査を許容する

(5) プロトコール治療中止患者に対する HRQoL 調査

病勢の進行，合併症の発生や増悪，有害事象などによりプロトコール治療中止となった場合には，HRQoL の評価は中止とする。

(6) 調査方法

- ECOG PS は規定の調査項目として QoL 調査と同時期担当医が判定し，経過報告書に記載する。
- 患者の病状の悪化により患者本人が調査票に記入できない場合は CRC が調査票を読み上げて調査することも可とする。

(7) 欠測理由の調査

調査予定時期{5.5(4))を超え，当該対象患者から調査票の返送がない場合，データセンターは担当医に調査票の配布状況，対象者の健康状態など，欠測の理由を調査する。

欠測理由は以下に分類し，解析報告書，研究結果の公表（学会発表，論文など）に研究対象者のプロファイルとして記載する。

① 対象者の病状の悪化
② 対象者の死亡
③ 対象者の調査拒否
④ 対象者の記入・送付忘れ・紛失
⑤ 対象者の要因による，上記以外の理由
⑥ 担当者から対象者への調査票の配布忘れ

(8) HRQoL 調査予定患者数

本研究への全登録者を調査対象とする。

5.6 データの提出

本研究では症例登録票は FAX，それ以外の症例報告書は Electronic Data Capture（EDC）システムにより CSPOR データセンターへ提出する。

担当医師または臨床研究コーディネーター（CRC）等は，本研究に登録されたすべての患者を対象として，研究完了まで研究の進捗にあわせて CSPOR データセンターに症例報告書を提出する。CRC 等が症例報告書を記入する場合は，担当医師の確認を得る。

提出する症例報告書の種類，送付・提出の手段および時期を以下に示す。

No.	種類	送付手段，時期	提出手段，時期
1	症例登録票	参加施設へ予め郵送	登録時 FAX 送信<br>プロトコール治療開始<br>予定日前 2 週以内
2	プロトコール治療<br>開始報告書	EDC	EDC<br>プロトコール治療開始後 2 週以内に提出
3	経過報告書<br>（ex.有害事象，効果判定）	EDC	EDC<br>3，6ヶ月目の調査は，調査の規定日から2ヶ月以内に提出
4	経過報告書<br>（ex.後治療）	EDC	EDC<br>プロトコール治療の終了後1年毎の調査<br>調査の規定日から2ヶ月以内に提出
5	経過報告書<br>（ex.生存）	EDC	EDC<br>プロトコール治療の終了後1年毎の調査<br>調査の規定日から2ヶ月以内に提出
6	緊急有害事象連絡書	院内書式の使用も可<br>参加施設へ予め郵送	FAX 送信<br>発現を知ってから 72 時間以内

5.7 報告書への記載項目

(1) 症例登録票

① 記入日, 施設名, 担当医師名, 患者識別番号, 施設電話番号, 返信用 FAX 番号

② 年齢, 身長, 体重

③ ER および PgR の発現状況(J-score 分類で, 0%, 1-9%, 10%以上)

④ 適格基準(はい／いいえ 同意については同意取得日, PS は 0, 1, 2 を選択)

⑤ 除外基準(はい／いいえ 各項目に該当しないことを確認)

⑥ 予定治療, および開始予定日

(2) プロトコール治療開始報告書

① 病歴：(5.3.2 (1)の内, ホルモン受容体の発現, HER2 の発現状況を除く項目)

② 登録前画像検査の実施状況：5.3.2 (3)

③ 病巣所見：測定可能病変の有無, 標的病変の部位・測定結果, 非標的病変の記述

④ 有害事象

⑤ 併用薬

⑥ 開始した薬剤と選択理由, 開始した日

(3) 経過報告書：プロトコール治療の実施中

中止のない限り, プロトコール治療開始から, ３, ６ヶ月目。

３ヶ月目の調査でプロトコール治療の中止が報告された場合, ６ヶ月目の報告は不要。

① 体重, PS

② プロトコール治療開始後の画像検査の実施状況：5.3.4 (2)および 5.3.5 (2)

③ 病巣所見：標的病変の部位・測定結果, 非標的病変の記述

④ 有害事象

⑤ 併用薬, 併用治療と実施理由

⑥ プロトコール治療中止の有無, 中止となった場合は中止理由と中止日

⑦ プロトコール治療のコンプライアンス

⑧ 時点総合効果

(4) 経過報告書：後治療

① 後治療の調査：化学療法の実施日と種類

(5) 経過報告書：予後調査

① 増悪の有無と確認日

② 最終生存確認日, 死亡した場合は死亡日と死亡原因

(6) 緊急有害事象報告書

(Appendix E を参照)

5.8 データの取り扱い

CSPOR データセンターは別に定めるデータマネジメント計画（Standard Operating Procedure とマニュアル）に従って，提出されないデータの督促，提出されたデータの精査と問い合わせ，問い合わせ結果に基づくデータ修正，データベース管理を行う。CSPORデータセンターは入力されたデータをもとにモニタリング用の資料および統計解析用データセットを作成する。

5.9 予定症例登録数と研究期間

（1） 目標症例登録数

200 例以上（ただし，下記の登録期間内であれば，目標数到達後も登録を継続）

（2） 研究期間

研究期間：2015 年 11 月～2019 年 10 月までの 4 年間

内，登録期間：2015 年 11 月～2016 年 10 月までの 1 年間

内，観察期間：2016 年 11 月～最後の登録から 3 年間

5.10 解析・分析の方法

（1） 解析集団の定義

解析で用いる解析対象集団について以下のように定義する。

全適格例：「5.2 症例の登録」に従って登録された患者のうち，重複登録や誤登録を除いた集団を「全適格例」とする。

全治療例： 全適格例から，予定のプロトコール治療を 1 日一度でも施行された集団を「全治療例」とする。

（2） 評価項目

本研究における評価項目は，以下のように規定する

① Primary endpoint(主要評価項目）

- 臨床的有用率（clinical benefit rate: CBR）

② Secondary endpoint（副次的評価項目）

- 無増悪生存期間（progression free survival: PFS）
- 全生存期間（overall survival: OS）
- 治療成功期間（time to treatment failure: TTF）
- 化学療法開始までの期間（time to chemotherapy: TTC）
- 奏効率（response rate: RR）
- 健康関連 quality of life
- 有害事象 （Toxicity）

(3) 解析計画

① 主たる解析

二次内分泌療法全般とともに，コホートごとに Primary endpoint である CBR が 30%を超えるかどうかを 2 項分布に基づく正確な検定と対応する 90%信頼区間により検証する。ただし，解析対象集団は全治療例とし，有意水準は片側 5%とする。

② その他の解析

二次内分泌療法の効果予測因子としての意義を検証するため，一次内分泌療法への反応性，ER 発現強度，PgR 発現の有無に関して，CBR に関する交互作用の解析を，全登録例を対象として行う。各因子に関しては，以下の分類を用いる。

A) 一次内分泌療法への反応性

- Very low sensitive 群：術後内分泌療法開始後 2 年未満の再発，もしくは転移乳がんに対する一次治療に対して 3 ヶ月未満での増悪（p25, 4.2(2)の図 1 の very low に相当）
- Low sensitive 群：術後内分泌療法開始後 2-5 年の再発，もしくは転移乳がんに対する一次治療に対して 3 ヶ月-9 ヶ月での増悪（2.2.3 図 2 の low に相当）

B) ER 発現強度

- ER low 群：ER 弱陽性（J-score 分類が 1-9%）
- ER high 群：ER 陽性(J-score 分類が 10%以上）

C) PgR 発現の有無

- PgR negative 群：PgR 陰性（J-score 分類が陰性）
- PgR positive 群：PgR 陽性（J-score 分類は 1%以上）

Secondary endpoints もコホートごとに解析を行う。2値エンドポイントである response で定義される RR に関しては，点推定値と2項分布に基づく正確な 90%信頼区間を求める。また，time-to-event エンドポイントである PFS，OS，TTF，TTC については，Kaplan-Meier 法により生存曲線を推定し，各イベント時点の生存確率の 90%信頼区間を Greenwood の公式により求める。primary endpoint と同様，効果予測因子に関して交互作用の解析を行う。

有害事象の解析は，全治療例を対象とする。報告された有害事象に対し，種類別・グレード別の発現割合を計算する。

③ HRQoL の解析

合計スコアとして算出される，FACT-G，Breast，ES は総スコア，ならびにサブドメインスコアをスコアリングマニュアルに従い算出し，対象者全体の各調査時点における基本統計量を算出する。

また，尺度ごとに同定されている Minimally important difference (MID)を臨床的に意味のある QoL スコア低下の閾値とし，各測定ポイントにおいて登録時のスコアより閾値を上回るスコア低下症例を個別に同定し，QoL の維持率を算出する。

推移の質問により得られた情報は，内分泌療法期間中の MID 同定のための主観的アンカーとして用い，すでに確立されている MID との相違を検討する。

HRQoL の評価と解析は，本研究の主要評価項目である臨床的有用率に影響を及ぼさないと考えられるため，データの収集後（最後の登録症例における 3 ヶ月目の QoL 調査票の回収後）直ちに解析を実施する。解析結果は，学会発表，論文等により公表する。

6　研究対象者の選定方針

6.1　対象集団の定義

『一次内分泌療法に良好な臨床効果の得られなかった閉経後エストロゲン受容体陽性 HER2 陰性の転移乳がん患者』をどのように定義づけるかに関するコンセンサスは確立していないが，本研究では，

- 術後補助内分泌療法を継続中で，かつ開始から 5 年以内に再発した症例
- 転移乳がんの一次内分泌療法に対して，開始から 9 ヶ月以内に病勢進行した症例

と設定した。これらは 4.2 (2) の図 1 でいう，“very low”，“low”に相当する。

本研究では，前述した臨床経過の定義に沿う症例を，**『一次内分泌療法低感受性乳がん』**と呼ぶ。

6.2　選択基準

以下の条件をすべて満たす症例を，本研究の対象とする。

(1)　組織学的に乳がんと診断された[注1]エストロゲン受容体(Estrogen Receptor: ER)陽性[注2]の閉経後[注3]乳がん患者。

注 1:がんの組織亜形は問わない。

注 2:ER 陽性の判定は乳房の原発巣での判定を原則とし，ER 陽性の定義 3.6 に従う。ただし，原発巣での評価が困難な場合，転移巣の評価で ER 受容体陽性と判定された場合は，登録可能とする。また，原発巣と転移巣での評価に相違が認められた場合は，原発巣の評価結果をもって，ER 発現の評価とする。

注 3:閉経の条件は以下の少なくとも1つを満たすこと

①　60 歳以上

②　60 歳未満で無月経が 1 年以上(ただし子宮摘出を受けていないこと)

③　両側卵巣摘出を受けている

被験者の閉経の有無が定かでない場合は，FSH 及び血漿エストラジオール濃度が各施設基準で閉経後の範囲にあることを確認する。

(2)　転移乳がんの診断について，以下のいずれかに該当する。
測定可能病変の有無は問わない。

①　初診時に手術適応とならない遠隔転移を有する StageⅣ乳がん。
(3.3 臨床病期分類を参照)

②　治癒を目的とした乳がんの初期治療後(手術およびその前後の治療の後)に，遠隔転移にて増悪あるいは再発した乳がんである。
ただし，根治切除が可能な局所再発(術側の上方は鎖骨下縁，下方は肋骨弓，内側は胸骨正中線，外側は広背筋前縁に囲まれた胸壁を指す)は除く。

(3) 転移乳がんに対する，内分泌療法が予定されていること。

(4) ECOG の Performance Status (PS) が 0 もしくは 1 である注4。
（3.5 PS の評価を参照）
注 4: 骨転移に伴う活動性の低下のため PS2 と判定された症例は適格とする。

(5) 乳がんに対する過去の内分泌療法について，以下のいずれかに該当する。
なお，使用された内分泌療法薬の種類は問わない。

① 術後補助療法として継続して内分泌療法薬が投与され，かつ内分泌療法の開始から **5 年以内に再発した症例**。

② 転移乳がん注5に対する一次治療として内分泌療法が施行され，かつ内分泌療法の開始から **9 ヶ月以内に病勢が増悪した症例**。

注 5: 術後内分泌療法が 5 年間以上実施され，内分泌療法開始後 5 年目以後に転移した症例は含めない。また，術後内分泌療法が何らかの理由により 5 年以内に中止され，その後に転移再発した症例も含めない。

(6) 乳がんに対する過去の化学療法について，以下のいずれかに該当する。
① 投与されたことがない。
② 術前，あるいは術後補助療法として化学療法剤の投与が実施されている場合，最終投与日より 6 ヶ月(168 日，24 週)以上経過している。

(7) 乳がんに対する過去の放射線療法について，以下に該当する。
① 最終照射日から 14 日以上が経過している。

(8) 同意書(Appendix G)により，対象者本人から研究参加への同意が得られている。

6.3 除外基準
以下の1つでも該当する場合，本研究の対象としない。
(1) HER2 陽性乳がん （3.7 HER2 発現状況の判定を参照）

(2) 内分泌療法の適応とはならない症例。

(3) その他，医師が本研究への参加を不適切と判断した症例。

7　研究の科学的合理性の根拠

7.1　評価項目の設定根拠

がん治療の有効性と安全性を評価する単一の指標は確立されていないため，本研究では複数エンドポイントモデルとした。

統計解析における多重比較の問題から，本研究では一次内分泌療法低感受性乳がんに対する，近年の内分泌療法薬全般により得られる臨床的恩恵の評価指標として，臨床的有用率（Clinical benefit rate: CBR）を主要評価項目に設定した。

また，転移乳がんの治療の目標は，生命予後の改善，症状の緩和，HRQoL の維持と改善であることから，患者アウトカムとして全生存期間，有害事象，HRQoL を副次的評価項目に設定した。腫瘍関連アウトカムとしては，無増悪生存期間，治療成功期間，奏効率を副次的評価項目とした。

7.2　研究仮説の科学的合理性と根拠

閉経後乳がんに対する，術後補助内分泌療法薬としても，あるいは転移乳がんの一次内分泌治療薬としても，現在の第一選択薬はアロマターゼ阻害薬である。従って，本研究への登録例の大半が，先行治療としてアロマターゼ阻害剤が投与されていると想定される。アロマターゼ阻害剤による先行治療後の，二次内分泌療法に関する臨床試験成績としては，以下のような報告がある。

【タモキシフェン】

- 転移乳がんに対する一次内分泌療法として，タモキシフェンとアナストロゾールを比較した TARGET trial では，アナストロゾール群の二次内分泌療法として 137 例がタモキシフェンの投与を受けており，48.7%と CBR が得られている[25)]。
- 同 TARGET trial のスイスで実施されたサブスタディー（SAKK 21/95 sub-trial）でも，アナストロゾール後のタモキシフェンにより 50%の CBR が得られている[26)]。

【他のアロマターゼ阻害剤】

- 非ステロイド系アロマターゼ阻害剤（アナストロゾール，レトロゾール）の次治療として，ステロイド系エキサメスタンの治療効果を検討した報告は 9 件あり，CBR は 12-55%であった[27)]。

【フルベストラント】

- 非ステロイド系アロマターゼ阻害剤（アナストロゾール，レトロゾール）の次治療として，ステロイド系エキサメスタンとフルベストラント LD レジメン（初回 500mg を筋注後，day14，28 に 250mg，以後 28 日毎に 250mg）とを比較した EFECT Trial では，治療効果は同等であり，CBR はフルベストラントで 32.2%，エキサメスタンで 31.5%であった[28)]。
- 術後内分内分泌療法中の再発患者を対象としたフルベストラントの用量比較試験（250mg/month vs 500mg/month）では，CBR が 250mg/month 群で 39.6%，500mg/month 群で 45.6%と高く，全生存期間でも 500mg 群の優越性が示されている[29, 30)]。

【mTOR 阻害剤：エベロリムスと内分泌療法との併用】

- 非ステロイド系アロマターゼ阻害剤（アナストロゾール，レトロゾール）の次治療として，エキサメスタンと mTOR 阻害剤エベロリムス＋エキサメスタンを比較した BOLERO-2 試験では，エベロリムス併用の優越性が示され，CBR はエキサメスタン群で 59%，エベロリムス＋エキサメスタン群で 79.6%であった [15, 16)]。
- アロマターゼ阻害剤の次治療として，タモキシフェンと mTOR 阻害剤エベロリムス＋タモキシフェンを比較した TAMRAD 試験では，CBR はタモキシフェン群で 42.1%，エベロリムス＋タモキシフェン群で 61.1%と，エベロリムス併用の優越性が示された [17)]。

以上のように，試験間，薬剤によりばらつきがあるものの，アロマターゼ阻害剤の次治療でも 50%程度の CBR が得られている。殊に，近年の臨床研究でのフルベストラント 500mg，mTOR 阻害剤の併用により，高い CBR が得られる傾向にある [31)]。

本研究では，一次内分泌療法低感受性乳がんを対象としており，これまでの試験結果を必ずしもあてはめることができず，その治療効果は低く見積もる必要がある。その一方で，臨床的に許容される，最低限の治療効果が保証されなければならない。

本研究では，目的のひとつ{4.1 目的（1）}を検証するための仮説として，　4.3 研究仮説(1)に示したように，一次内分泌療法低感受性乳がんに対する，二次内分泌療法の CBR が 30%を下回らなければ治療選択肢の一つになるとした。本研究では，一次内分泌療法低感受性乳がんに対する二次内分泌療法全般の有効性を検証するとともに，以下のように薬剤種別の有効性も検証する{4.3 研究仮説 (2)}。

- コホート SERMs
  Selective Estrogen Receptor Modulators: SERMs であるタモキシフェン，トレミフェンが選択された場合。

- コホート AIs
  Aromatase inhibitor: AI であるアナストロゾール，レトロゾール，
  エキサメスタンが選択された場合。

- コホート SERD
  Selective Estrogen Receptor Downregulator: SERD である
  フルベストラントが選択された場合。

- コホート with mTORi
  内分泌療法薬に mTOR 阻害剤であるエベロリムスの併用が選択された場合。

7.3 目標症例数の設定根拠

本研究では，治療選択は医療者と患者の選好に基づいて実施する観察研究であるため，各コホートの比較を目的としない。本仮説を検証するため，各コホートで閾値臨床的有用率 30%，期待臨床的有用率 50%と設定し，正確な二項検定を想定してα＝0.05（片側），β＝0.2 の下での必要症例数は 43 例と推定され，10%程度の脱落を想定し，50 例を各コホートの目標症例数と設定した。

本研究は観察研究であり，各コホートに，必ずしも均等に分布するわけではないが，最高でも4群への分類[(4)薬剤選択]に対応したサンプルサイズを設定する必要がある。従って，最低 200 例を目標症例数に設定した。なお，登録期間内であれば，全登録数 200 例に達しても，4 群すべてで目標の 50 例に到達するまで登録を継続する。

8　患者の保護

本研究に関係するすべての研究者及び共同研究者はヘルシンキ宣言および文部科学省と厚生労働省が共同で策定した「人を対象とする医学系研究に関する倫理指針」（平成 26 年 12 月 22 日）を遵守して本研究を実施する。

9　インフォームドコンセントを受ける手続き等

担当医師は，登録に先立ち施設の倫理審査委員会で承認された説明文書を本研究の対象候補者に渡し，口頭で十分に説明する。説明後，研究の対象候補者が質問する機会と判断するのに十分な時間を与え，研究の対象候補者が研究の内容をよく理解したことを確認した上で研究への参加を依頼し，本人の自由意思による同意を同意書で取得する。

研究への参加に同意が得られた場合，研究対象者は同意書に同意日を記入の上，捺印または署名する。また，説明を行った担当医師および（研究協力者が補足的な説明を行った場合は）研究協力者が各々の説明日を記入の上，記名捺印または署名する。同意書は複写を 2 部作成し，1 部は患者本人に手渡し，1 部は施設で保管する。原本はカルテに保管する。

インフォームドコンセントにあたっては，研究対象者に以下の項目について説明する。

(1) この研究の名称と，研究実施までの手続きについて
(2) 研究機関の名称と研究責任者の氏名
(3) 研究の目的と意義
(4) 研究の方法と期間
(5) 研究の対象者として選定された理由
(6) 研究参加に伴う負担と危険性、利益
(7) 研究への参加後の，同意の撤回について
(8) 研究に参加されない場合，同意撤回後の治療について
(9) 研究の情報公開
(10) 研究資料の閲覧と入手について
(11) 個人情報の取り扱い
(12) 収集した情報の保管と廃棄の方法
(13) 研究の資金と利益相反について
(14) 研究の相談窓口
(15) 費用の負担と謝礼について
(16) 他の治療方法について
(17) 研究終了後の治療について
(18) 研究に関する新たな情報の提供について
(19) 健康被害に対する補償について
(20) 収集した情報の研究利用と譲渡について
(21) 収集した情報の第三者の閲覧について
(22) 施設の研究責任者・担当医の連絡先

10 個人情報の取り扱い

(1) プライバシーの保護

プライバシーおよび個人情報の保護のため，本研究実施に関わる者は，研究対象者のプライバシーおよび個人情報の保護に十分配慮する。本研究で得られた研究対象者のデータは本研究の目的以外には使用しない。なお，研究の結果を公表する際も研究対象者を特定できる情報は使用しない。

(2) 連結可能匿名化

登録患者の同定や照会は，登録時に発行される登録番号(連結可能匿名化)を用いて行い，患者名，イニシャル，生年月日など第三者が直接患者を識別できる情報は，CSPOR データセンターのデータベースに登録しない。

(3) 匿名化の時期と方法

匿名化は，症例の登録時に，CSPOR データセンターで実施する。

「症例登録票」に記載された患者識別番号，及び登録施設に対し，CSPOR データセンターは登録番号を発行する。データセンターは「症例登録確認通知書」に発行された登録番号を記載し，担当医に送付する。

以後，対象者のデータ収集(QoL 調査票を含め)，問い合わせで必要な対象者の識別には登録番号を用いる。対応表は，CSPOR データセンターで厳重に管理，保管する。

11 研究対象者に生じる負担並びに予想されるリスク及び利益

本研究で実施される治療は，日常臨床の範疇を超えるものではなく，また治療は医療者と患者の選好に基づき実施される。また，治療効果や副作用の評価のために実施される，画像評価の手法，頻度も日常臨床で実施されている範疇を超えるのもではない。したがって，研究参加に伴う研究対象者のリスクの増加や利益はないものと考えられる。

本研究では，調査票を用いた QoL 評価を実施するが，１回の調査時間は 15 分以内，調査回数は登録時，プロトコール治療開始から１，３ヶ月目の３回であり，研究対象者にはこれらの記載のための負担が生じる。本研究に用いる QoL 調査票は，回答者への心理的負担に関する内容妥当性が検証されており，対象者への過度の心理的負担はなく，侵襲は軽微と考えられる。

12 試料・情報の保管及び廃棄の方法

12.1 保管

被験者の同意に関する記録，報告書作成のための基礎データ（検査データ等），倫理委員会の承認書，医療機関において作成された記録文書については，研究責任者の管理の基，厳重に保管する。ただし，これらの資料の保管方法（保管場所，保管責任者）が，（共同）研究施設で定められている場合はこれに従う。保管にあたっては、個人情報の漏洩等のないよう，十分留意する。

保管期間は，研究の中止または終了後 5 年を経過した日までとする。

12.2 廃棄

情報の廃棄時には個人の情報の含まれた電子データの消去，紙媒体はシュレッダー処理するなどして，個人情報の漏洩のないよう十分留意する。

13 研究機関の長への報告内容及び方法

13.1 研究機関の長への報告内容

（共同）研究機関の研究責任者は，以下のような内容を，研究機関の長に報告する。

なお，各研究機関で研究機関の長への報告内容が定められている場合は，これに従う。

- 研究の進捗状況
- インフォームドコンセント手続きの実施状況
- 個人情報の管理状況
- 研究期間中の問題点の有無
- 問題点があった場合はその内容及びそれに対する対応等

13.2 報告の時期と方法

各研究機関で，研究機関長への報告時期と方法が定められている場合は，これに従う。

各研究機関で規定がない場合，研究責任者は 13.1 の内容を含んだ，

「研究実施状況等報告書」を作成し，研究機関長に報告する。

報告する時期は，以下とする。

- 毎年度末

- 研究終了報告

14 研究資金・利益相反

14.1 研究資金

本研究は，アストラゼネカ株式会社の Externally Sponsored Research (ESR)プログラムの内，Investigator Initiated-Sponsored Research (IISR)（医師主導研究）から（社）CSPOR-BC への資金提供による支援に基づき実施する（http://www.astrazeneca.com/Research/externally-sponsored-research）。

本研究の計画，実施機関/施設及び倫理委員会の承認，研究の実施，研究結果の分析・解釈・および公表，研究の透明性の確保に関する責務は，全て研究者が責任を持ち，アストラゼネカ株式会社はこれらの意思決定には関与しない。

学会，論文発表の際には，以上を明記する。

14.2 利益相反状態の開示

（1） 本研究の実行委員の利益相反状態の開示

以下に，本研究実行委員会の利益相反状態を開示する。

なお，状態の開示は日本乳癌学会より示された「乳癌臨床研究の利益相反状態開示に関する指針（2015 年 4 月 1 日更新）」および「乳癌臨床研究の利益相反状態開示に関する指針細則（2015 年 6 月 9 日）」に準じた（http://www.jbcs.gr.jp/AboutJBCS/rieki.html）。

研究者氏名と本研究での役割	該当の状況＊（2015 年 7 月 31 日現在）
平　成人（研究代表者）	2014 年　アストラゼネカ社より『アストラゼネカ研究サポート』として奨学寄附金
藤澤　知巳（研究代表者）	すべての項目に該当なし
荒木　和浩（実行委員）	すべての項目に該当なし
岩本　高行（実行委員）	すべての項目に該当なし
坂巻顕太郎（生物統計解析責任者）	すべての項目に該当なし

＊開示項目：役員・顧問職；株；特許使用料；講演料など；原稿料など；

（2） （共同）研究機関における研究責任者の利益相反状態の開示

本研究の倫理審査に際して，（共同）研究機関の倫理審査委員会の定める処により，研究責任者の利益相反状態の開示が必要な場合，研究責任者は自らの利益相反状態の開示を行う。 報告の様式は，研究機関の規定に従う。

15 研究に関する情報公開の方法

15.1 研究の概要及び結果の登録

本研究では，最初の研究対象者の組み入れまでに UMIN 臨床試験登録システム（UMIN Clinical Trials Registry, UMIN-CTR, http://www.umin.ac.jp/ctr/index-j.htm）に登録し，研究計画を情報公開する。また，研究計画書の変更及び研究の進捗に応じて適時更新し，研究を終了したときは，遅滞なく，研究の結果を登録する。

15.2 研究結果の公表

研究を終了したときは，遅滞なく，研究対象者等及びその関係者の人権又は研究者等 及 び そ の関係者の権利利益の保護のために必要な措置を講じた上で、研究の結果を 公表する。研究成果の発表は，本研究の実行委員会の提案をもとにCSPOR-BC運営委員会が決定する。本研究に関する発表は別途定める事前の発表計画に基づき適宜実施する。発表形式は，学会発表および医学雑誌への論文投稿とする。

16 研究対象者等及びその関係者からの相談等への対応

研究対象者等及びその関係者からの相談等への対応先として，以下を説明文書に記載する。

- 本研究代表者の氏名，所属，連絡先
- 研究機関の研究責任者の氏名，所属，連絡先
- その他，（共同）研究機関が指定する相談窓口がある場合は，その連絡先

17 代諾者からのインフォームドコンセントについて

本研究では代諾者を置かない。

18 研究対象者の経済的負担・謝礼

本研究の診療内容は日常臨床の範疇で行われる。従って，研究参加中の薬剤，検査を含む診療費はすべて研究に参加する患者の保険診療および自己負担によって支払われる。

本研究参加に対する研究対象者への謝礼等はない。

19 有害事象の評価と報告

有害事象とは，研究開始以降に研究対象者に発現したあらゆる好ましくない症状および徴候（臨床検査値の異常を含む）と定義し，治療との因果関係の有無を問わない。

有害事象が発現した場合，担当医師は速やかに必要な処置（検査，有害事象の治療，治療中止など）を行い，研究対象者の安全の確保に努める。

19.1 有害事象の評価

有害事象名とGradeの判定は，有害事象共通用語規準 v4.0日本語訳JCOG版に従う。有害事象のうち，各薬剤の添付文書に副作用として記載さているものを既知の有害事象，記載されていないものを未知の有害事象とする。

（1） 有害事象の評価対象期間

本研究での有害事象の評価期間は，登録時から評価の対象と定めたプロトコール治療の終了後，30 日までとする。

（2） 評価の対象とする有害事象

本研究で対象者に実施される治療・検査は日常臨床の範疇で実施される。従って，本研究への参加に伴う対象者の有害事象に関する危険性は，日常臨床と同様である。このため本研究では，grade2 以下の有害事象については，原則として収集しない。Grade3 以上の有害事象に関しては，担当医師は有害事象名，最悪 Grade，初回発現日を「経過報告書」（Appendix X）に記録する。Grade の判定および有害事象名は，CTCAE v4.0 に従う。

（3） 治療関連死の取り扱い

治療関連死の場合，原因となった有害事象を Grade 5 とする。なお，治療関連死の場合は緊急報告｛19.2 (1)｝を行う。緊急報告を含む事後の検討により原因となった有害事象の最終的な Grade が決定される。

19.2 有害事象の報告

下記に該当する報告義務のある有害事象が生じた場合，研究責任医師は事務局へ
報告を行う。なお，薬剤との因果関係が否定できない重篤な有害事象（ICH E2A で定めるところの重篤）が認められた場合には，医薬品医療機器等法第 68 条の 10（副作用の報告）医薬品等安全に沿って，研究責任医師の責任において適切に行う。

（1） 緊急報告義務のある有害事象

以下のいずれかに該当する有害事象は，「緊急有害事象連絡書」（Appendix X）により緊急報告する。これらの有害事象は「重大な有害事象」とする。

① 評価の対象と定めた内分泌療法中のすべての死亡

② 評価の対象と定めた内分泌療法の最終投与後 30 日以内，かつ次治療開始前のすべての死亡。

「30 日」とは治療日を Day 0 とし，その翌日から数えて 30 日を指す。この期間内におきた死亡については，評価の対象と定めた内分泌療法治療との因果関係の有無は問わず，すべてを緊急報告の対象とする。

ただし，上記期間外（評価の対象と定めた内分泌療法の最終投与後 30 日以降，または次治療開始後）におきた死亡であっても，評価の対象と定めた
内分泌療法との関連性が疑われる場合は緊急報告の対象とする。

③ 次治療開始前までに認められた，未知の Grade 4 の非血液毒性

（2） 通常報告義務のある有害事象

緊急報告義務のある有害事象{19.2 (1)}にあてはまらない，Grade3 もしくは Grade4 に相当る有害事象。

（3） 研究責任医師の報告義務と報告手順

① 緊急報告

緊急報告の対象となる有害事象{19.2 (1)}が発生した場合，担当医師は速やかに研究責任医師に伝える。研究責任医師が対応できない場合は担当医師が研究責任医師の責務を代行する。

緊急報告の対象となる有害事象が観察された場合には，24 時間以内に
事務局へ口頭で報告するとともに，研究責任医師は有害事象発生を知ってから **72 時間以内**に「緊急有害事象連絡書」（Appendix X）（院内書式にて作成も可）に所定事項を記入し CSPOR データセンターへ FAX 送信（03-5298-8536）またはメールで送信（info@csp.or.jp）する。

さらに，研究責任医師はより詳しい情報を記述した症例報告（A4 自由書式）を別紙として作成し，有害事象発生を知ってから **15 日以内**に CSPOR データセンターへ FAX 送信する。

② 通常報告

研究責任医師は当該有害事象の発生時期に対応する「経過報告書」（Appendix X）に所定事項を記録し，経過報告の提出時期に CSPOR データセンターへ送付する。

19.3 事務局の責務

（1） 登録停止と施設への緊急通知の必要性の有無の判断

研究責任医師から報告を受けた事務局は，報告内容の緊急性，重要性，影響の程度等について研究代表者あるいはその代行者の判断を仰ぎ，必要に応じて登録の一時停止（CSPOR データセンターと全参加施設へ連絡）や参加施設への周知事項の緊急連絡等の対策を講ずる。

また，医薬品医療機器等法の副作用の報告の実施を報告施設の研究責任医師に強く促す。

(2) 独立データモニタリング委員会への報告

研究代表者は，施設から緊急報告もしくは通常報告された有害事象が，「報告義務のある有害事象」に該当すると判断した場合，**有害事象の発生を知り得てから15日以内に独立データモニタリング委員会に文書で報告**し，同時に当該有害事象に対する研究代表者の見解と有害事象に対する対応の妥当性についての審査を依頼する。

19.4 独立データモニタリング委員会での検討

独立データモニタリング委員会は報告内容を審査し，症例の取り扱いや登録継続の可否を含む今後の対応について研究代表者に文書で勧告する。

20 健康被害に対する補償

本研究参加中の研究対象者に有害事象が発現した場合，担当医師は速やかに必要な対応（検査，治療，研究中止など）を行い，研究対象者の安全の確保に努める。その際，保険診療の範囲内での最善の医療を提供するものとする。本研究においては，健康被害への金銭による補償は行わない。

21 研究実施終了後における医療の提供に関する対応

本研究におけるプロトコール治療終了後の治療は規定しない。
また，本研究で実施される医療行為は日常臨床の範疇で実施されるため，研究実施終了後における医療の提供に関する特別な対応は行わない。担当医は，転移乳がんの治療目標である生存期間の延長，症状の緩和，QoL の維持改善を目標として，最善の医療を提供する。

22 研究対象者の健康状態に係わる情報，および研究結果の取り扱い

担当医師は，研究対象者が研究に参加している間に，本研究への継続参加について研究対象者の意思に影響を与える可能性のある情報，あるいは研究の結果が得られた場合は，直ちに当該情報を記載した説明資料を提供し，これに基づき，以下について研究対象者に説明する。

- 当該情報について
- 本研究への継続参加については自由であること

説明文書・同意書には，説明を行った担当医師が，その日付を記入の上記名捺印または署名し，研究対象者は情報受領日を記入の上記名捺印または署名する。なお，研究協力者が補足的な説明を行った場合には，研究協力者もその日付を記入の上記名捺印または署名する。当該資料の写しは研究対象者に提供する。

担当医師は，本研究に継続して参加するか否かについての意思を研究対象者に確認し，説明文書・同意書の原本に意思確認日およびその確認結果を記入し，保存する。担当医師は，説明文書・同意書を改訂し，必要に応じ施設の倫理審査委員会の承認を得た後，研究対象者に改訂された説明文書・同意書を用いて改めて説明し，本研究への参加の継続について研究対象者から自由意思による同意を文書により得る。その際，担当医師は，当該同意書に説明日を記入の上記名捺印または署名し，研究対象者も同意日を記入の上記名捺印または署名する。同意文書の写しは研究対象者に提供し，原本を保存する。

23 研究業務の委託，当該業務内容及び委託先の監督方法

23.1 データメネジメントの委託先

CSPOR データセンター
NPO 日本臨床研究支援ユニット(J-CRSU)内
J-CRSU データセンター
代表（データセンター長）
大橋靖雄　　　　（中央大学理工学部人間総合理工学科 ）
〒113-0034 東京都文京区湯島 1-10-5 湯島 D&A ビル 1F
TEL：03-3254-8029
FAX：03-5298-8536
E-mail：trial-bc@cspor-bc.or.jp

23.2 業務の内容

- 症例登録業務
- 進捗管理業務
- データマネジメント業務

23.3 監督の方法

本研究の実行委員は，25.1 に記載したモニタリングに併せ， CSPOR データセンターが 23.2 の業務を適切に実施できているかどうかを監督，指導する。

24 試料・情報の将来利用について

本研究で研究対象者から取得された情報について，同意を受ける時点では特定されない将来の研究のために用いられる可能性はない。また，他の研究機関に提供する可能性もない。

将来，本研究で研究対象者から取得された情報を，本研究とは異なる目的で研究利用する場合，あるいは他の研究機関に提供しようとする場合は，改めて研究計画書を作成し，倫理審査委員会の承認の基に実施する。

25 モニタリング及び監査

25.1 モニタリング

研究が安全に，かつ本研究実施計画書に従って実施されているか，データが正確に収集されているかを確認する目的でモニタリングを実施する。

モニタリングは中央モニタリング（in-house monitoring）とし，CSPOR データセンターに収集された症例報告書等を対象に，電子化されたデータの処理結果を参考として，実行委員とCSPORデータセンターが協力して行う。施設訪問によるモニタリングは予定しない。

25.2 監査

本研究は，「人を対象とする医学系研究に関する倫理指針」（平成 26 年 12 月 22 日公布）で示され

た「高度な侵襲を伴う介入研究」に相当しないため，監査は予定しない。

26 倫理審査委員会での承認

26.1 研究への参加開始時の承認

本研究への参加を開始する際には，各施設は必要な書類を提出し，施設の倫理審査委員会で本研究への参加を承認されなければならない。施設の倫理審査委員会の承認が得られた場合，承認文書のコピーを事務局へ送付する。承認文書原本は施設で保管し，コピーは事務局が保管する。

26.2 倫理審査委員会承認の年次更新

本研究実施計画書および患者への説明文書に関する，各施設の倫理審査委員会の審査・承認の年次更新については，各施設の規定に従う。

27 研究計画の遵守，変更

27.1 研究の終了，中止，中断

（1） 研究の終了

すべての施設において，研究期間終了までの追跡が終了した時点を本研究終了とする。各施設の研究終了時には，研究責任医師は速やかに研究終了報告書を研究実施施設の長ならびに研究代表者に提出する。

（2） 研究の中止，中断

① 独立データモニタリング委員会は，必要に応じ研究継続の妥当性を検討する。同委員会は，研究の継続が適切でないと判断した場合には，研究代表者に研究の中止あるいは中断を勧告する。

研究代表者が勧告に従い研究の中止を決定した場合には，可及的速やかに中止とその理由，参加者に対する対応方法を研究責任医師に伝達する。

研究責任医師は経緯を研究実施施設の長に文書で報告すると同時に，当該施設の倫理審査委員会に報告し，研究代表者と当該施設の倫理審査委員会の指示に従い，研究への参加者に対して適切な対応をとる。

② 倫理審査委員会により中止の勧告または指示があった場合，研究代表者および研究責任医師は以下に従い適切な対応をとる。

A） 倫理審査委員会から研究代表者に対して研究中止の勧告または指示があった場合，研究代表者は研究の中止について検討し，中止を決定した場合には，可及的速やかに中止とその理由，研究に参加中の対象者に対する対応方法を研究責任医師に伝達する。

研究責任医師は経緯を研究実施施設の長に文書で報告すると同時に，当該施設の倫理審査委員会に報告し，研究代表者と当該施設の倫理審査委員会の指示に

従い，研究に参加中の対象者に対して適切な対応をとる。

B） 各施設の倫理審査委員会から研究責任医師に対して研究中止の勧告または指示があった場合，研究責任医師は速やかに研究代表者に報告する。報告をうけた研究代表者は独立データモニタリング委員会に報告し，独立データモニタリング委員会は研究継続の妥当性を検討する。研究責任医師は，当該施設の倫理審査委員会からの勧告または指示に基づき研究の中止または中断を決定した時は，速やかに研究実施施設の長にその理由とともに文書で報告する。

③ 研究責任医師は，以下の事項に該当する場合は研究実施継続の可否を検討する。

A） 研究対象者のリクルートが困難で予定研究対象者数の達成が，困難であると判断したとき。

B） 予定する研究対象者数または研究予定終了日に達する前に研究の目的が達成されたとき。

C） 独立データモニタリング委員会，もしくは倫理審査委員会により研究計画等の変更の指示があり，これを受入れることが困難と判断されたとき。

なお，独立データモニタリング委員会とは，(社)CSPOR-BC が組織する(社)CSPOR-BC 独立データモニタリング委員会を指す。

27.2 研究実施計画書の遵守

本研究を行う研究者は，参加者の安全と人権を損なわない限りにおいて本研究実施計画書を遵守する。

27.3 研究実施計画書からの逸脱

(1) 担当医師は，研究代表者の事前の合意および当該施設の倫理審査委員会の事前の審査に基づく研究実施施設の長の承認を得る前に，研究実施計画書からの逸脱あるいは変更を行ってはならない。

(2) 担当医師は，緊急回避等のやむを得ない理由があると判断した場合は，研究代表者との事前の合意および当該施設の倫理審査委員会の事前の承認を得る前に，研究実施計画書からの逸脱あるいは変更を行うことができる。その際には，担当医師は，逸脱または変更の内容および理由ならびに研究実施計画書等の改訂が必要であればその案を速やかに，研究代表者および当該施設の倫理審査委員会に提出し，研究代表者，当該施設の倫理審査委員会および研究実施施設の長の承認を得るものとする。

(3) 担当医師は，研究実施計画書からの逸脱があった場合は逸脱事項をその理由とともに全て記録する。研究責任医師は逸脱事項を研究代表者に報告し，その写しを保存しなければならない。

27.4 研究実施計画書の変更

(1) 研究実施計画書の変更の区分

試験審査委員会，運営委員会および倫理審査委員会の承認後の研究実施計画書の変更を，改正・改訂の 2 種類に分けて取り扱う。定義と取り扱いは以下の通り。

① 改正（Amendment）

研究の参加者の危険（risk）を増大させる可能性のある，もしくは研究の主要評価項目に関連する研究実施計画書の部分的変更。独立データモニタリング委員会および倫理審査委員会の審査承認を要する。カバーページに独立データモニタリング委員会の承認日を記載する。

② 改訂（Revision）

研究の参加者の危険を増大させる可能性がなく，かつ研究の主要評価項目にも関連しない研究実施計画書の変更。

独立データモニタリング委員会の審査は不要だが，研究代表者の承認と独立データモニタリング委員会への報告を要する。各施設の倫理審査委員会の審査・承認については，各施設の取り決めに従う。

カバーページに研究代表者の承認日を記載する。

(2) 研究実施計画書の改正/改訂時の施設倫理委員会承認

研究中に独立データモニタリング委員会の承認を得て本研究実施計画書もしくは患者への説明文書の改正がなされた場合は，改正された研究実施計画書および説明文書が各施設の倫理審査委員会で承認されなければならない。

内容変更が改正ではなく改訂の場合に，各施設の倫理審査委員会の審査・承認を要するか否かは，各施設の取り決めに従う。

改正に対する施設倫理審査委員会の承認が得られた場合，各施設の研究責任医師は倫理審査委員会の承認文書のコピーを事務局へ送付する。倫理審査委員会承認文書の原本は研究責任医師が保管し，コピーは事務局が保管する。

28 知的財産権の帰属

本研究の研究結果によって生じる特許，その他の知的財産に関する権利は(社)CSPOR-BC に帰属する。

29 参考文献

1) 日本乳癌学会編. 臨床・病理 乳癌取り扱い規約(第 17 版). 金原出版, 2012.
2) 乳 が ん HER2 検 査 病 理 部 会 . HER2 検 査 ガ イ ド 乳 癌 編 第 四 版 . http://pathology.or.jp/news/pdf/HER2-150213.pdf
3) Eisenhauer EA, Therasse P, Bogaerts J, Schwartz LH, Sargent D, Ford R, Dancey J, Arbuck S, Gwyther S, Mooney M, Rubinstein L, Shankar L, Dodd L, Kaplan R, Lacombe D, Verweij J. New response evaluation criteria in solid tumours: revised RECIST guideline (version 1.1). Eur J Cancer. 2009; 45(2):228-47.
4) 国 立 が ん 研 究 セ ン タ ー が ん 対 策 情 報 セ ン タ ー . が ん 情 報 サ ー ビ ス . http://gdbganjohojp/graph_db/index?lang=ja 2001.
5) Bonadonna G, Hortobagyi GN, Valagussa P. Textbook of breast cancer: a clinical guide to therapy, CRC Press, 2006.
6) Yamamoto N, Watanabe T, Katsumata N, Omuro Y, Ando M, Fukuda H, et al. Construction and validation of a practical prognostic index for patients with metastatic breast cancer. Journal of clinical oncology : official journal of the American Society of Clinical Oncology 1998; 16(7): 2401-8.
7) Watanabe T. Evidence produced in Japan: tegafur-based preparations for postoperative chemotherapy in breast cancer. Breast cancer (Tokyo, Japan) 2013; 20(4): 302-9.
8) 日本乳癌学会. 科学的根拠に基づく乳癌診療ガイドライン 1 薬物療法(2015 年版). 金原出版 2015.
9) Cardoso F, Costa A, Norton L, Senkus E, Aapro M, André F, et al. ESO-ESMO 2nd international consensus guidelines for advanced breast cancer (ABC2). Annals of Oncology 2014: mdu385.
10) Chlebowski RT. Strategies to overcome endocrine therapy resistance in hormone receptor-positive advanced breast cancer. Clinical Investigation 2014; 4(1): 19-33.
11) DeFriend DJ, Anderson E, Bell J, Wilks DP, West CM, Mansel RE, Howell A. Effects of 4-hydroxytamoxifen and a novel pure antioestrogen (ICI 182780) on the clonogenic growth of human breast cancer cells in vitro. Br J Cancer. 1994;70(2):204-11.
12) Dauvois S, White R, Parker MG. The antiestrogen ICI 182780 disrupts estrogen receptor nucleocytoplasmic shuttling. J Cell Sci. 1993;106 (Pt 4):1377-88.
13) DeFriend DJ, Howell A, Nicholson RI, Anderson E, Dowsett M, Mansel RE, Blamey RW, Bundred NJ, Robertson JF, Saunders C, et al. Investigation of a new pure antiestrogen (ICI 182780) in women with primary breast cancer. Cancer Res. 1994;54(2):408-14.
14) Robertson JF, Llombart-Cussac A, Feltl D, et al. Fulvestrant 500 mg versus anastrozole as first-line treatment for advanced breast cancer: Overall survival from the phase II "first" study. 2014 San Antonio Breast Cancer Symposium; Abstract S6-04.
15) Baselga J, Campone M, Piccart M, Burris HA 3rd, Rugo HS, Sahmoud T, Noguchi S, Gnant M, Pritchard KI, Lebrun F, Beck JT, Ito Y, Yardley D, Deleu I, Perez A, Bachelot T, Vittori L, Xu Z, Mukhopadhyay P, Lebwohl D, Hortobagyi GN. Everolimus in postmenopausal hormone-receptor-positive advanced breast cancer. N Engl J Med. 2012;366(6):520-9.
16) Piccart M, Hortobagyi GN, Campone M, Pritchard KI, Lebrun F, Ito Y, Noguchi S, Perez A, Rugo HS,

Deleu I, Burris HA 3rd, Provencher L, Neven P, Gnant M, Shtivelband M, Wu C, Fan J, Feng W, Taran T, Baselga J. Everolimus plus exemestane for hormone-receptor-positive, human epidermal growth factor receptor-2-negative advanced breast cancer: overall survival results from BOLERO-2. Ann Oncol. 2014;25(12):2357-62.

17） Bachelot T, Bourgier C, Cropet C, Ray-Coquard I, Ferrero JM, Freyer G, Abadie-Lacourtoisie S, Eymard JC, Debled M, Spaëth D, Legouffe E, Allouache D, El Kouri C, Pujade-Lauraine E. Randomized phase II trial of everolimus in combination with tamoxifen in patients with hormone receptor-positive, human epidermal growth factor receptor 2-negative metastatic breast cancer with prior exposure to aromatase inhibitors: a GINECO study. J Clin Oncol. 2012;30(22):2718.

18） Cella DF. Measuring quality of life in palliative care. Semin Oncol 1995; 22(2 Suppl 3): 73-81.

19） Shimozuma K, Ohashi Y, Takeuchi A, et al. Feasibility and validity of the Patient Neurotoxicity Questionnaire during taxane chemotherapy in a phase III randomized trial in patients with breast cancer: N-SAS BC 02. Support Care Cancer 2009; 17(12): 1483-1491.

20） Basch E, Abernethy AP, Mullins CD, et al. Recommendations for incorporating patient-reported outcomes into clinical comparative effectiveness research in adult oncology. J Clin Oncol 2012; 30(34): 4249-4255.

21） Stockler M, Wilcken NR, Ghersi D, et al. Systematic reviews of chemotherapy and endocrine therapy in metastatic breast cancer. Cancer Treat Rev 2000; 26(3):151-168.

22） Fallowfield LJ, Leaity SK, Howell A, Benson S, Cella D: Assessment of quality of life in women undergoing hormonal therapy for breast cancer: validation of an endocrine symptom subscale for the FACT-B. Breast Cancer Res Treat. 1999;55:189-99.

23） Cella DF, Tulsky DS, Gray G, Sarafian B, Linn E, Bonomi A, et al.: The Functional Assessment of Cancer Therapy scale: development and validation of the general measure. J Clin Oncol. 1993;11:570-9.

24） Brady MJ, Cella DF, Mo F, Bonomi AE, Tulsky DS, Lloyd SR, et al.: Reliability and validity of the Functional Assessment of Cancer Therapy-Breast quality-of-life instrument. J Clin Oncol. 1997;15:974-86.

25） Thürlimann B, Robertson JF, Nabholtz JM, Buzdar A, Bonneterre J; Arimidex Study Group. Efficacy of tamoxifen following anastrozole (‘Arimidex’) compared with anastrozole following tamoxifen as first-line treatment for advanced breast cancer in postmenopausal women. Eur J Cancer. 2003; 39(16):2310-7.

26） Thürlimann B, Hess D, Köberle D, Senn I, Ballabeni P, Pagani O, Perey L, Aebi S, Rochlitz C, Goldhirsch A. Anastrozole (‘Arimidex’) versus tamoxifen as first-line therapy in postmenopausal women with advanced breast cancer: results of the double-blind cross-over SAKK trial 21/95—a sub-study of the TARGET (Tamoxifen or ‘Arimidex’ Randomized Group Efficacy and Tolerability) trial. Breast Cancer Res Treat. 2004; 85(3):247-54.

27） Beresford M, Tumur I, Chakrabarti J, Barden J, Rao N, Makris A. A qualitative systematic review of the evidence base for non-cross-resistance between steroidal and non-steroidal aromatase inhibitors in metastatic breast cancer. Clin Oncol (R Coll Radiol). 2011; 23(3):209-15.

28） Chia S, Gradishar W, Mauriac L, Bines J, Amant F, Federico M, Fein L, Romieu G, Buzdar A, Robertson JF, Brufsky A, Possinger K, Rennie P, Sapunar F, Lowe E, Piccart M. Double-blind, randomized placebo controlled trial of fulvestrant compared with exemestane after prior nonsteroidal aromatase inhibitor therapy in postmenopausal women with hormone receptor-positive, advanced breast cancer: results from EFECT. J Clin Oncol. 2008; 26(10):1664-70.

29） Di Leo A, Jerusalem G, Petruzelka L, Torres R, Bondarenko IN, Khasanov R, Verhoeven D, Pedrini JL, Smirnova I, Lichinitser MR, Pendergrass K, Garnett S, Lindemann JP, Sapunar F, Martin M. Results of the CONFIRM phase III trial comparing fulvestrant 250 mg with fulvestrant 500 mg in postmenopausal women with estrogen receptor-positive advanced breast cancer. J Clin Oncol. 2010; 28(30):4594-600.

30） Di Leo A, Jerusalem G, Petruzelka L, Torres R, Bondarenko IN, Khasanov R, Verhoeven D, Pedrini JL, Smirnova I, Lichinitser MR, Pendergrass K, Malorni L, Garnett S, Rukazenkov Y, Martin M. Final overall survival: fulvestrant 500 mg vs 250 mg in the randomized CONFIRM trial. J Natl Cancer Inst. 2014;106(1):djt337.

31） Massarweh S1, Romond E, Black EP, Van Meter E, Shelton B, Kadamyan-Melkumian V, Stevens M, Elledge R. A phase II study of combined fulvestrant and everolimus in patients with metastatic estrogen receptor (ER)-positive breast cancer after aromatase inhibitor (AI) failure. Breast Cancer Res Treat 2014 Jan;143(2):325-32.

30 添付書類（Appendix）

A） 文部科学省及び厚生労働省より平成 26 年 12 月 22 に公開された「人を対象とする医学系研究に関する倫理指針」

B） 症例登録票

C） 症例登録確認通知書

D） 経過報告書

E） :緊急有害事象報告書

F） QoL 調査票

G） 同意書
